# Morphogenetic theory of mental and cognitive disorders: the role of neurotrophic and guidance molecules

**DOI:** 10.3389/fnmol.2024.1361764

**Published:** 2024-04-03

**Authors:** Alexandra Primak, Kirill Bozov, Kseniya Rubina, Stalik Dzhauari, Elena Neyfeld, Maria Illarionova, Ekaterina Semina, Dmitriy Sheleg, Vsevolod Tkachuk, Maxim Karagyaur

**Affiliations:** ^1^Faculty of Medicine, Lomonosov Moscow State University, Moscow, Russia; ^2^Federal State Budgetary Educational Institution of the Higher Education “A.I. Yevdokimov Moscow State University of Medicine and Dentistry” of the Ministry of Healthcare of the Russian Federation, Moscow, Russia; ^3^Institute for Regenerative Medicine, Medical Research and Education Center, Lomonosov Moscow State University, Moscow, Russia

**Keywords:** brain morphogenesis, mental and cognitive disorders, schizophrenia, depression, autism, neurotrophines, guidance molecules

## Abstract

Mental illness and cognitive disorders represent a serious problem for the modern society. Many studies indicate that mental disorders are polygenic and that impaired brain development may lay the ground for their manifestation. Neural tissue development is a complex and multistage process that involves a large number of distant and contact molecules. In this review, we have considered the key steps of brain morphogenesis, and the major molecule families involved in these process. The review provides many indications of the important contribution of the brain development process and correct functioning of certain genes to human mental health. To our knowledge, this comprehensive review is one of the first in this field. We suppose that this review may be useful to novice researchers and clinicians wishing to navigate the field.

## 1 Introduction

Psychiatric and cognitive disorders is a global issue for modern society. According to data from the World Health Organization for the year 2022, such disorders occur in 1 out of 8 people (World Health Organization, 2022). There are multiple theories laying the grounds for the development of cognitive and psychiatric disorders; however, most of them pinpoint an imbalance between excitatory and inhibitory structures in the brain as the primary reason (Walsh et al., 2008). Such an imbalance can be caused by dysfunction of the nervous system (deficiency in an enzymatic system responsible for neurotransmitter production, disturbance of neurotransmitter secretion and reuptake, defects in the structure and function of neurotransmitter receptors, channelopathies) as well as organic damage or developmental abnormalities in the brain. Furthermore, as supported by numerous genetic and twin studies, heredity is considered an underlying cause for a variety of psychiatric and cognitive disorders such as schizophrenia, endogenous depression, affective disorders and autism (Kallmann, 1946; Gardner and Stephens, 1949; Detera-Wadleigh et al., 1987; Egeland et al., 1987; Gassó et al., 2015).

The human brain, serving as the biological substrate for consciousness and cognitive functions, is extraordinarily intricate. It typically consists of around 100 billion neurons, alongside a multitude of neuroglial cells that provide support to these neurons. Each neuron establishes multiple connections with up to 10,000 other neurons, transmitting signals through approximately 1,000 trillion synapses (Stiles and Jernigan, 2010).

The proper functioning of the brain is ensured by correct morphological and structural development of different brain parts as well as precise synaptic connections of individual neurons. The development of such a complex structure relies on the sophisticated orchestration of multiple cellular and molecular mechanisms, since their disruption or imbalance can have devastating consequences and result in a vast array of neurodevelopmental and cognitive disorders. A prime example of this principle in action is schizophrenia. In several studies, the authors reported a perturbation of spatial neuron positioning, reduced neuron density and density of neural connection in the hippocampus, prefrontal cortex, and anterior cingulate gyrus (Grafton et al., 1992; Tamminga et al., 1992). In autism spectrum disorders deviations in brain development have also been reported, typically manifesting as uneven and disproportional brain growth, enlarged white matter relative to gray matter, and a delayed gray matter formation (Torun et al., 2015; Lukito et al., 2020). Of note, autosomal recessive primary microcephaly is associated with abnormalities in the survival and proliferation of embryonic neural progenitors (Stiles and Jernigan, 2010). Aberrations in embryonic corticogenesis is related to neurodevelopmental disorders such as lissencephaly, megalencephaly, schizophrenia, Rett Syndrome, intellectual disorders, Down syndrome, and meningeal heterotopias (Costa et al., 2007; Meyerink et al., 2020). All of those mentioned above ascertain the core importance of correct embryonic brain structure development for its normal functioning and mental health in the adult individuum (Walsh et al., 2008; Stiles and Jernigan, 2010; Gassó et al., 2015; Ishii et al., 2016; Lukito et al., 2020). Comparing to other theories of the innate nature of mental disorders, the neurodevelopmental theory of the origin of psychiatric and cognitive disorders is significantly underestimated. Since comprehensive reviews on the subject are still lacking, here we aim to fill in the gap and congregate the existing data on the potential mechanisms of neurodevelopmental abnormalities as well as on the key neurotrophic factors and guidance molecules related to these pathological conditions. Detailed research (including the stage of prenatal development) may allow for potential mental disorder prediction as well as provide a solid ground for new strategies, such as gene therapy, to guide future treatment-prevention approaches (Sasi et al., 2017).

## 2 Key stages of neurodevelopment, guidance molecules, and secreted factors

Human brain development starts in the third week of gestation and continues until late adolescence. It involves neurogenesis, neuronal migration, establishment of neuronal connection, axonal growth and myelination. Disruption of any of these courses is considered to be responsible for a wide spectrum of pathologies (Johnson et al., 2009; Corroenne et al., 2022). Neurodevelopment is affected by gene expression as well as by environmental conditions encompassing the presence and variety of information stimuli (Miguel et al., 2019).

### 2.1 Early stages of human brain development

To shed light on the pathogenesis of psychiatric and cognitive disorders, we will briefly consider the underlying neurodevelopmental mechanisms in the brain with a focus on the individual molecules.

Embryonic nervous system development starts with the differentiation of epiblast cells into neural progenitor cells (neuroectodermal cells) as a result of complex molecular signaling that involves multiple gene expression. Sonic Hedgehog (SHH) and antagonists of Bone Morphogenetic Protein 4 (BMP4): chordin, noggin, and follistatin, which are produced by notochord cells, are among the most important signaling molecules (Greene and Copp, 2009; Stiles and Jernigan, 2010; Sears et al., 2022). By the end of gastrulation, the neural progenitor cells are located along the rostral-caudal midline of the upper part of the three-layered embryo forming a structure referred to as a neural plate. The neuroectoderm ridges formed along the sides of the neural plate (E21) rise folding inward and then fuse, starting at the neural plate center and continuing in both, the rostral and caudal parts, forming a hollow neural tube. By the 26th–27th day, the neural tube closes along the entire length. This stage of embryonic development, from the neural plate formation until the neural tube is complete, is known as neurulation (Greene and Copp, 2009; Stiles and Jernigan, 2010).

Neuroepithelial cell proliferation within the walls of the rostral end of the neural tube and the internal pressure of cerebrospinal fluid lead to the formation of three primary brain vesicles: the precursor of the forebrain (prosencephalon—the anterior vesicle), midbrain (mesencephalon—the middle vesicle), and hindbrain (rhombencephalon—the posterior vesicle) (Fame et al., 2020). The rostral-lateral parts of the forebrain grow faster than the others, leading to the formation of two outgrowths (diverticula) in the rostral part—the so-called telencephalon, which further develop into the cerebral hemispheres. The caudal part of the forebrain develops into the diencephalon, which gives rise to the intermediate brain (epithalamus, metathalamus, thalamus, and hypothalamus). The mesencephalon gives rise to the midbrain, and the rhombencephalon divides into two segments: the metencephalon (which gives rise to the pons and cerebellum) and the myelencephalon (which gives rise to the medulla oblongata) (Greene and Copp, 2009; Stiles and Jernigan, 2010; Fame et al., 2020).

Proliferation of the neuroblasts lining the neural tube and their radial outward migration from the central part ultimately results in the architectural complexity and multilayered structure of the neural tube. Different regions of the neural tube exhibit peculiarities reflected in the formation of specific brain and spinal cord segments (Delhaye-Bouchaud, 2001; Barry et al., 2013). Particularly, in the region of the brain vesicles the process results in the formation of the cortex, basal ganglia, and white matter as discussed below in more detail (Delhaye-Bouchaud, 2001; Barry et al., 2013).

### 2.2 Migration of neurons in the developing brain

**Figure 1:**
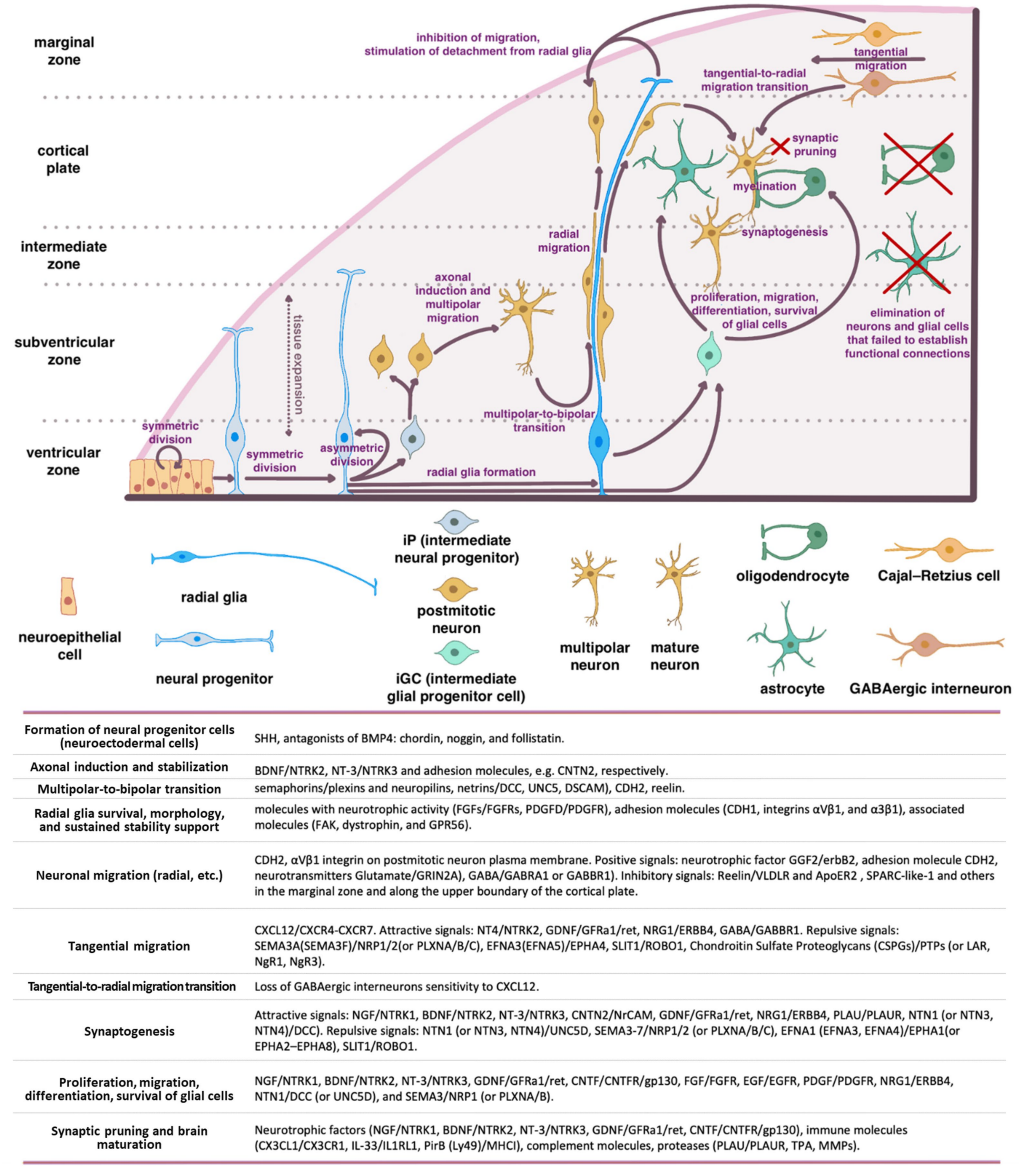
Schematic representation of the main stages of brain morphogenesis with the indication of key regulatory molecules.

Neuronal positioning is crucial for the formation of distinct brain regions such as the cerebral cortex, hippocampus, and cerebellum. In humans, from the end of gastrulation and until E42, the population of neural progenitor cells, located in the ventricular zone (VZ) of neural tube, undergo mitotic divisions, producing identical daughter cells (“symmetric division”) (Figure 1). However, starting from E42, the type of cell division in neural progenitors progressively shifts toward an asymmetric one, resulting in one neural progenitor cell and one neuron. Most of the postmitotic neurons leave the VZ and migrate into the developing cortex. However, a certain population of neurons maintain their position and will later develop into the basal ganglia. At the same time, the neural progenitors remain in the proliferation zone and proceed with the asymmetric divisions (Wodarz and Huttner, 2003; Stiles and Jernigan, 2010).

A series of coordinated migrations take place in the developing cortex when neurons migrate from the site of origin to their final destination to form complex dendritic and axonal networks there. Three main types of migration have been described: radial (from the center of the neural tube), tangential (along the surface of the developing brain), and multipolar.

The basis for radial migration are so-called radial glia cells, which established a strong mechanical connection in the early stages of corticogenesis, both with the pial (outer) surface of the cortex and with the basal layer in the ventricular zone (proliferation zone). Migrating cortical neurons physically associate with bipolar radial glia and migrate along with their ascent to the top of the cortex early in corticogenesis. As the cortex grows and thickens, glial cells can stretch for millimeters, guiding the migration of neurons even along the migratory paths with inhibitory signals and layers of the previously migrated neurons (Wodarz and Huttner, 2003; Stiles and Jernigan, 2010).

The first migrating neurons form the preplate—primordial plexiform layer above the ventricular zone, which restricts the outward neuron migration and helps organize the layers of the developing cortex. Subsequently, neurons migrate within the preplate to form four or six layers of the mature cortex (depending on its type) (Rice and Curran, 2001; Cooper, 2008). The second wave of migrating neurons splits the preplate into two separate layers marginal zone (MZ) and subplate (SP). Both the MZ and SP disappear at the end of fetal period (Stiles and Jernigan, 2010).

The neurons that form deeper cortical layers (V–VI) migrate earlier, while those forming the superficial layer (II) migrate later resulting in the “inside-out” pattern of development. Experiments on neural progenitor cell transplantation demonstrated that this migration is strongly affected by the microenvironment, which encompasses a complex interplay of cells, molecules, and blood vessels that surround and provide support to them (Lathia et al., 2007; Kazanis et al., 2008; National Cancer Institute, 2024). Although, the regulatory mechanisms remain unclear, the radial glia to a large extent determines the migratory paths of the neurons and the speed of migration (Rice and Curran, 2001; Cooper, 2008). This form of migration is particularly crucial for pyramidal glutamatergic neurons that populate the appropriate layers of the cerebral cortex (Luhmann et al., 2015).

According to the current concepts of embryonic development, radial migration of postmitotic neurons is normally preceded by their multipolar migration in the subventricular and intermediate zones of the developing brain (Tabata and Nakajima, 2003). Migrating multipolar neurons undergo axonal induction that makes them to protrude an axon that is stabilized by intercellular adhesion molecules (e.g., CNTN2).

BDNF/NTRK2 and NT-3/NTRK3 are among the most important and well-known neurotrophic factors that induce the axonal growth in multipolar neurons. The shift in neuron migration from multipolar to radial one is a critical step (termed a multipolar-to-bipolar transition), which is orchestrated by guidance molecules such as semaphorins (receptors—plexins and neuropilins) and netrins (receptors—DCC, UNC5, DSCAM), as well as by CDH2 and Reelin (Cooper, 2014).

In this intricate process, molecules with neurotrophic activity (including FGFs/FGFRs and PDGFD/PDGFR), adhesion molecules (CDH1, integrins αVβ1, and α3β1), and associated molecules such as focal adhesion kinase (FAK), dystrophin, and GPR56, significantly contribute to supporting radial glia survival, morphology, and sustained stability (Penisson et al., 2019). During radial migration (relevant for other types of neuron migration), neurons are exposed to gradients of positive and negative signals, ensuring their precise positioning in the developing cortex. Among the key positive signals are: neurotrophic factor GGF2 (glial growth factor-2, a soluble form of neuregulin1)/erbB2, adhesion molecule CDH2 (N-cadherin) expressed on the surface of radial glial cells, and neurotransmitters Glutamate (signals via GRIN2A), GABA (signals via GABRA1 or GABBR1) (Anton et al., 1997; Luhmann et al., 2015). Experimental findings provide compelling evidence that CDH2 and αVβ1 integrin expression on the plasma membrane of postmitotic neurons are essentially important for their migration (Meyerink et al., 2020; Rashid and Olson, 2023). Soluble proteins, including Reelin, SPARC-like-1, and others distributed within the marginal zone and along the upper boundary of the cortical plate, provide inhibitory signals that restrict the radial neuron migration (Chai and Frotscher, 2016).

Cajal–Retzius cells (CR) that have colonized the superficial layer of the developing cortex (so-called marginal zone) produce Reelin, that triggers the detachment of radial migrating neurons from radial glia, inhibits migrating neurons from exiting beyond the cortex, and instructs them to take up proper positions within the forming cortical plate. To date, two receptors for Reelin on the surface of migrating neurons have been identified: very low-density lipoprotein receptor (VLDLR) and apolipoprotein E receptor 2 (ApoER2) (Dlugosz and Nimpf, 2018).

SPARC-like-1 operates in a similar manner, inducing the detachment of migrating neurons from the radial glial cell processes at the upper boundary of the cortical plate. The source of SPARC-like-1 is believed to be the radial glial cells themselves, with its expression and secretion being particularly prominent in the apical segment of radial glial cell processes. As of today, a receptor for SPARC-like-1 on the surface of neurons remains undefined (Dlugosz and Nimpf, 2018).

Cajal–Retzius cells (CR) primarily originates from the cortical hem, ventral pallium, and pallial septum. Unlike neurons migrating from the VZ, the primary type of CR migration is tangential, traversing long distances along the surface of the cortex. This type of migration enables CR to be the initial cell population of the superficial marginal zone in the developing cortex. CR orchestrate the subsequent colonization of the cortical plate by postmitotic neurons undergoing radial migration (Paxinos and Marín, 2015). Modern concepts of brain morphogenesis consider CR cells as a transient population within the cerebral cortex. Once they have served their purpose, these cells undergo programmed cell death (Elorriaga et al., 2023).

The tangential migration is also recognized as a predominant mode of migration for GABAergic interneurons (INs). These neurons hold a crucial role in synchronizing the activity of excitatory glutamatergic pyramidal neurons in the cerebral cortex and organizing the transmitted information (Llorca and Deogracias, 2022). Any misplacement or dysfunction of these interneurons may potentially contribute to the development of certain psychiatric disorders.

The rodent cerebral cortex comprises three primary types of interneurons (INs): parvalbumin-positive (PV+), somatostatin-positive (SST+), and 5HTR3a-positive neurons. PV+ INs establish connections with the soma, proximal dendrites, and proximal axon segments of pyramidal neurons. SST+ INs target the distal dendrites of pyramidal neurons, interact with basket-like PV+ INs within cortical layer IV, and form reciprocal connections with layer IV Purkinje cells (Naka et al., 2019). Additionally, 5HTR3a+ INs predominantly populate the surface layers of the neocortex, exerting inhibitory effects on other INs and thereby mediating the disinhibition of pyramidal neurons (Pfeffer et al., 2013; Jiang et al., 2015; Favuzzi et al., 2019).

Cortical INs originate from distinct compartments within the ventral subpallium: the medial ganglionic eminence (MGE) and preoptic area (POA) give rise to PV+ and SST+ INs. In contrast, the caudal ganglionic eminence (CGE) produces 5HTR3a+ INs, while the lateral ganglionic eminence (LGE) serves as the primary source of GABAergic projection neurons for the striatum (Llorca and Deogracias, 2022).

IN precursors initially emerge from neural progenitors located in the ventricular and subventricular zones of the subpallium. Subsequently, they migrate to their designated layers within the developing brain, such as the marginal zone (MZ), cortical plate, or they may remain within the subventricular zone and undergo tangential migration. Notably, PV+ and SST+ INs primarily migrate within the MZ, and their migration trajectory is determined by their origin and cellular identity. During migration through various regions of the developing cortex, INs encounter molecular cues that determine their fate, phenotype, and electrophysiological characteristics—a process often referred to as spatial and temporal patterning (Butt et al., 2007; Jovanovic and Thomson, 2011; Allaway et al., 2021). Once they arrive at their designated cortical regions, they undergo radial migration to integrate into the developing neural circuits (Llorca and Deogracias, 2022).

Tangential migration is defined by a diverse array of signaling pathways distinct from the radial migration. Of note, the CXCL12/CXCR4-CXCR7 signaling axis plays a pivotal role in guiding the tangential migration of GABAergic interneurons (Llorca and Deogracias, 2022). Loss of sensitivity to CXCL12 triggers a transition from tangential to radial migration, resulting in their entry into the developing cortical plate and the establishment of connections with pyramidal neurons (Marín, 2013; Luhmann et al., 2015). In addition to the CXCL12/CXCR4-CXCR7 axis, a wide spectrum of neurotrophic and guidance signals are involved in tangential migration. These signals encompass both attractive cues, such as BDNF/NTRK2, NT4/NTRK2, GDNF/GFRa1/ret, HGF/c-Met, VEGFA/FLT1(FLT4, FLK1, NRP1, NRP2), NRG1(NRG3)/ERBB4, GABA/GABBR1, dopamine/D1, and repulsive signals including SEMA3A (or SEMA3F)/NRP1/2 (or PLXNA/B/C), EFNA3 (or EFNA5)/EPHA4, SLIT1(SLIT2)/ROBO1, FLRT2(FLRT3)/Unc5B (Unc5D), chondroitin sulfate proteoglycans (CSPGs)/PTPs (or LAR, NgR1, NgR3), dopamine/D2 (Marín, 2013; Fleitas et al., 2021; Llorca and Deogracias, 2022). The urokinase plasminogen activator (uPA)/urokinase plasminogen activator receptor (uPAR) system plays a significant role in the migration of interneurons (INs). This system is involved in various processes, including extracellular matrix degradation, cell migration, growth cone guidance, and angiogenesis (Semina et al., 2016, 2017; Toudji et al., 2023).

As INs mature and reach their designated cortical regions, their tangential migration halts, and a switch from tangential to radial migration occurs. Loss of sensitivity to CXCL12 coupled with increased sensitivity to GABA (Bortone and Polleux, 2009), along with activation of the NRG3/ERBB4 signaling axis are considered to be the main mechanisms launching tangential-to-radial switch (Llorca and Deogracias, 2022; Toudji et al., 2023). Additionally, microvessels and radial glia have been described as guiding tracks for the radially migrating INs in some studies (Yokota et al., 2007; Léger et al., 2020). Upon reaching the target cortical layer, INs establish connections with pyramidal neurons and other INs. The crucial stages of interneuron origin and development, as well as the main classes of molecules involved in these processes, are shown in the Supplementary Figure S1.

### 2.3 Establishing the interneuronal connections

In tandem with proliferation and migration of neural progenitor and their derivatives, the connections between the developing brain structures are established. This facilitates the coordination of brain structures activity and integrates the brain into a unified center for information processing, the center for generating motor and mental activity.

Two of the most important conducting pathways in the brain are the dopaminergic thalamocortical (TCT) and glutamatergic corticothalamic (CTT) tracts (Gasiorowska et al., 2021) because, by transmitting sensorimotor information, they connect the neocortex to sensory organs and target organs. In humans, the development of these pathways occurs between the 20th and 26th weeks of gestation (Kostović and Jovanov-Milosević, 2006). Subplate neurons play a pivotal role in shaping these pathways. Initially, they receive afferent fibers from thalamic nuclei (TCT) and act as guides for the growth of CTT fibers. As TCT (thalamus > neurons L4–L5) and CTT (neurons L5–L6 > thalamus) mature and stabilize, subplate neurons reduce their connections and gradually undergo apoptosis. Disturbances in the activity of subplate neurons or their premature demise can lead to abnormalities in the TCT or CTT formation, potentially causing the neurological and psychiatric disorders (Rubenstein et al., 2020).

Disruptions in dopaminergic pathways in the brain, particularly the mesolimbic (MLT) and mesocortical (MCT) tracts, play a fundamental role in the development of various psychiatric disorders, including schizophrenia, attention deficit hyperactivity disorder (ADHD), and addictions. These tracts establish connections between the ventral tegmental area (VTA) and the nuclei of the limbic system (such as the nucleus accumbens (NAcc), amygdala, and olfactory bulb) as well as with neurons in the prefrontal cortex (PFC) (Prakash and Wurst, 2006). NAcc, in turn, is considered a part of the ventral striatum, where the information from the PFC and the limbic system (including the hippocampus and amygdala) is integrated. It is widely believed that the NAcc is responsible for motivating the individuals to achieve specific goals and for seeking new behavioral patterns related to reward. The distinct functions within the NAcc are attributed to its core and shell regions. Experimental studies demonstrated that afferent fibers from the hippocampus and PFC terminate in the NAcc’s shell and core, respectively, exerting reciprocal effects on synaptic plasticity in these parts of the brain (Goto and Grace, 2008). Therefore, an imbalance in afferent signals to the NAcc from the PFC and the limbic system can bring about various psychiatric disorders. For example, a decrease in afferent signals from the PFC can lead to a long-term potentiation in the NAcc’s shell, triggering synaptic remodeling and the activation of reward-related behavioral patterns, potentially contributing to the development of addictions (Goto and Grace, 2008). Furthermore, hyperactivation of the hippocampus, often due to dysfunction or loss of parvalbumin GABAergic interneurons, and the subsequent transmission of excitatory signals from the hippocampus to the NAcc, amygdala, and PFC, are considered to be the key factors of both: positive and negative symptoms emerging in schizophrenia (Goto and Grace, 2008).

While the exact mechanisms underlying the formation and maturation of interneuronal connections remain incompletely understood, it is well-established that the precision and functionality of these connections are regulated by a plethora of neurotrophic and guidance molecules. These guidance molecules comprise both attractive cues, such as NGF/NTRK1, BDNF/NTRK2, NT-3/NTRK3, CNTN2/NrCAM, GDNF/GFRa1/ret, NRG1/ERBB4, PLAU/PLAUR, NTN1 (or NTN3, NTN4)/DCC and repulsive ones including NTN1 (or NTN3, NTN4)/UNC5D, SEMA3-7/NRP1/2 (or PLXNA/B/C), EFNA1 (EFNA3, EFNA4)/EPHA1(or EPHA2–EPHA8), SLIT1/ROBO1 (Niederkofler et al., 2010).

These signaling molecules also determine the development of the corpus callosum, a pivotal structure that provides the functional integration of the cerebral hemispheres. Disruptions in the corpus callosum development can give rise to seizures, as well as intellectual, coordination and psychiatric disorders (Niederkofler et al., 2010).

Radially migrating INs establish GABAergic connections with pyramidal neurons and other INs. The signaling axes of NRG3/ERBB4, BDNF/NTRK2, and NT4/NTRK2 are considered to play a key role in this process, as their disruption or malfunctioning increases excitability and oscillatory activity of pyramidal cells, as well as impaires the synchrony of oscillations between distinct cortical areas. In mutant mice, these functional disorders manifest as heightened locomotor activity, aberrant emotional responses, compromised working memory, altered social behavior, and impaired cognitive functions—symptoms specific to certain mental disorders (Curley and Lewis, 2012; Lewis et al., 2012; Del Pino et al., 2013). Interneurons (INs) that fail to establish functional connections undergo cell death (Llorca and Deogracias, 2022).

### 2.4 Development of brain glia and myelination of nerve fibers

Neurons need protection, support, and trophic supply, while nerve fiber need proper electrical isolation (myelin sheath) for efficient and rapid transmission of signals within and beyond the brain. In the brain, these vital functions are fulfilled by two distinct types of glial cells: oligodendrocytes and astrocytes.

Intermediate glial progenitor cells (iGCs), which are precursors of astrocytes and oligodendrocytes, are generated through the division of neural stem cells or radial glial cells located in the subventricular zone of the forebrain. These iGCs subsequently migrate to various regions including the white matter, cortex, striatum, and hippocampus, where they undergo differentiation into astrocytes and oligodendrocytes (Stiles and Jernigan, 2010). Notably, recent studies revealed the presence of glial progenitors in the marginal zone (MZ) of the developing cortex, potentially contributing to the upper cortical layers (Costa et al., 2007). Upon reaching their respective destinations, these glial progenitors establish connections with neurons and undergo differentiation to oligodendrocytes or astrocytes (Weng et al., 2019). Oligodendrocytes are responsible for myelinating the dendrites and axons, thereby facilitating the action potential conduction and providing neurotrophic support. Astrocytes, in turn, offer trophic support to neurons, participate in establishing the blood-brain barrier (BBB), and contribute to the formation and operation of functional synapses. The proliferation, migration, differentiation, and survival of glial cells are regulated by a set of key molecules, including NGF/NTRK1, BDNF/NTRK2, NT-3/NTRK3, GDNF/GFRa1/ret, CNTF/CNTFR/gp130, FGF/FGFR, EGF/EGFR, PDGF/PDGFR, NRG1/ERBB4, NTN1/DCC (or UNC5D), and SEMA3/NRP1 (or PLXNA/B) (Klämbt, 2009; Pöyhönen et al., 2019). Impairments in proliferation, migration of glial cells, or their dysfunction alter information transmission in the brain and are frequently linked to the onset of cognitive and psychiatric disorders (Zambon et al., 2017; Zhou et al., 2019; Ishikawa et al., 2020).

### 2.5 Maturation of the nervous system and elimination of non-functional connections

An essential stage in brain development is eliminating the neurons and glial cells that failed to establish functional connections with each other and did not integrate into neural networks (Rakic and Zecevic, 2000; Buss and Oppenheim, 2004). Additionally, synaptic pruning of non-functional (or excessive) synaptic connections occurs. This phenomenon arises from the competition of the established cells for limited resources, mostly for neurotrophic factors. Insufficient availability of these factors results in the demise of neurons and glial cells (Huang and Reichardt, 2001). Neurotrophic factors such as NGF/NTRK1, BDNF/NTRK2, NT-3/NTRK3, GDNF/GFRa1/ret, and CNTF/CNTFR/gp130, along with immune molecules like CX3CL1/CX3CR1, IL-33/IL1RL1, PirB (Ly49)/MHCI, and complement molecules, as well as proteases including PLAU/PLAUR, TPA, and MMPs, all play pivotal roles in brain maturation and synaptic pruning (Levi-Montalcini, 1964; Oppenheim et al., 1989; Faust et al., 2021). Microglial cells and astrocytes are currently recognized as the principal agents responsible for the natural process of synaptic pruning (Faust et al., 2021). Physiological death of non-functional neurons and cell populations (such as SP and MZ cells, including CR cells) that regulate migration and wiring predominantly takes place during the prenatal period.

An imbalance in the expression of these neurotrophic factors or disruptions in their signaling pathways results in excessive neuronal or glial cell death or, conversely, survival, which can lead to the onset of cognitive and psychiatric disorders (Walsh et al., 2008; Stiles and Jernigan, 2010; Bathina and Das, 2015; Angoa-Pérez et al., 2017; Zambon et al., 2017; Zhou et al., 2019; Ishikawa et al., 2020).

In the postnatal period, brain development persists with processes such as proliferation and migration of glial precursors, axon myelination, elimination of non-functional glial cells (those not connected to neurons), and synaptic pruning (Hughes, 2021). The list of key molecules governing these processes is detailed above (Levi-Montalcini, 1964; Oppenheim et al., 1989; Faust et al., 2021).

Therefore, the process of shaping the brain structures and their integration into a cohesive entity is intricate and multi-phased. Proliferation, migration, regulated cell death of neural precursors, glial cells, and transient cell populations, as well as the establishment of interneuronal connections, and myelination of nerve fibers are all orchestrated by an ensemble of molecular and cellular signals to ensure the correct development and maturation of the brain. At the same time the functions of individual brain regions are determined already at early stages of development. All together this provides a remarkable level of reliability in formation of functional and appropriately interconnected structures within the central nervous system, however, any dysfunction or malfunction of key coordinators in this process can result in disruptions to nervous system development and function (Bishop et al., 2002; Walsh et al., 2008; Stiles and Jernigan, 2010; Gassó et al., 2015).

A comprehensive study of these molecular families and the intricacies of their intracellular signaling is beyond the scope of the present review, given that the previously published literature is widely available (Levi-Montalcini, 1964; Oppenheim et al., 1989; Klämbt, 2009; Niederkofler et al., 2010; Pöyhönen et al., 2019; Faust et al., 2021). Our primary focus here is on data reflecting the dysfunction of key coordinator molecules and regulators of brain formation (neurotrophic and guidance molecules) in the development of mental and cognitive disorders.

## 3 Growth factors and guidance molecules implicating the impaired brain development and onset of cognitive and psychiatric disorders

The morphogenetic theory of mental disorders was first proposed by the Scottish psychiatrist Clouston (1891), and recent studies have only expanded the list of proteins and genes, whose dysfunction can lead to inappropriate formation of brain structures, the loss or aberrant inter-neuron connections ultimately manifesting in psychiatric and cognitive disorders.

### 3.1 Shh/Ptch signaling pathway

The Shh/Ptch signaling pathway is one of the first involved in the formation of the neuroectoderm, which gives rise to the nervous system. Therefore, loss-of-function mutations of the *Shh* gene, malfunction of its receptors or associated signaling cascades can lead to severe nervous system malformations that are often incompatible with life. These abnormalities include holoprosencephaly, cyclopia, the reduced number or absence of ventral cell types in the spinal cord, and anomalies of midline structures (Chiang et al., 1996).

Severe developmental defects leading to prenatal embryonic death are also associated with dysfunction of the BMP4 antagonist chordin (*Chrd* gene), resulting in abnormal development of the embryo in the ventrodorsal direction, ventralization of its tissues, and disruption of neuroectoderm formation (Bachiller et al., 2003; Troilo et al., 2014). As can be seen from the data described above, null mutations in *Shh*/*Ptch* or *Chrd* genes are lethal during the prenatal or early postnatal period. However, in some cases, non-lethal mutations such as VAR_023804 (R6T), rs760920236-C (S15W), rs28936675-T (G31R), and others in the *SHH* gene (The UniProt Consortium, 2023a), and rs145871696-G (T457S) in the *CHRD* gene (The UniProt Consortium, 2023b) downregulate the activity of these proteins, disrupt the formation of neuroectoderm, and may lay the basis for the onset of mental and cognitive disorders (holoprosencephaly, schizencephaly, autism, and others) (Nanni et al., 1999; Roessler et al., 2009). The genomic variants discussed in this review are summarized in Supplementary Table S1.

### 3.2 Neurotrophins and their receptors

Neurotrophins comprise a family of neurotrophic growth factors closely related to nerve growth factor (NGF). Within mammals, this family consists of four proteins: NGF, brain-derived neurotrophic factor (BDNF), neurotrophin-3 (NT-3), and neurotrophin-4/5 (NT-4/5). Neurotrophins are key molecules for sustaining proliferation and survival of neural progenitors and glial cells, as well as for the formation and maturation of inter-neuron connections, have also been shown to be involved in the manifestation of mental disorders (Mitre et al., 2017). For instance, in a number of patients with schizophrenia, a decrease in BDNF protein and mRNA has been detected in the hippocampus, prefrontal cortex, anterior cingulate cortex, and superior temporal gyrus (Hashimoto et al., 2005; Issa et al., 2010; Ray et al., 2011, 2014; Ray et al., 2014; Thompson et al., 2014), as well as the reduction of NTRK2 and NTRK3 (neurotrophin receptors) mRNA in the dorsolateral prefrontal cortex (Weickert et al., 2005). A lack of neurotrophins in embryonic tissues is observed during pregnancy in women with severe mental disorders (Freedman et al., 1992, 1994; Bersani et al., 2000), which reduces the proliferation and survival of neural progenitors during brain morphogenesis and can underlie the onset of mental illness or cognitive dysfunction. Moreover, studies have established a correlation between the incidence of single nucleotide polymorphisms (SNPs) in the genes *NGF, BDNF*, *NTRK1*, *NTRK2* and the susceptibility to paranoid schizophrenia (Arévalo et al., 2004, 2010; Lin et al., 2013; Kranz et al., 2015).

BDNF (brain-derived neurotrophic factor, *BDNF* gene), a classical neurotrophin, is recognized to be one of the key molecules affecting brain volume (Freedman et al., 1994). BDNF is expressed by both neurons and glial cells and, as mentioned above, is a molecule with broad neuroprotective activity. It controls the proliferation, survival, migration, and apoptosis of neural progenitors and mature neurons (including sensory and cholinergic neurons in the basal forebrain, mesolimbic dopaminergic neurons, hippocampal neurons, granular neurons of the cerebellum, etc.). BDNF is also involved in neurogenesis, synaptogenesis, and stabilization of the inter-neuron connections in the brain (Segal et al., 1992; Freedman et al., 1994; Sasi et al., 2017; Ping et al., 2022).

Impairments in BDNF expression and maturation may inflict the manifestation of cognitive and mental disorders at different levels: via the dysfunction of the brain dopaminergic system caused by dopaminergic neuron death, or via alterations in the plasticity/stability of inter-neuron networks. At the same time, *BDNF* gene is not included in the list of loci associated with the onset of schizophrenia (Pardiñas et al., 2018), which is due to the fact that the data on BDNF and predisposition to schizophrenia are inconsistent.

In 2020, Chinese scientists Ping et al. (2022) analyzed the association of the *BDNF* gene SNPs [rs11030101-A (intr), rs2030324-A (intr), and rs6265-C (V64V)] with predisposition to schizophrenia and found no significant correlation. However, when patients were subdivided using the positive and negative syndrome scale for schizophrenia (PANSS) scale, an association of rs1103010101-A with negative symptoms of schizophrenia and rs6265-C with attention deficit was determined. Analysis of *BDNF* gene haplotypes revealed that the rs11030101/rs2030324/rs6265 AAC haplotype was more common in patients with schizophrenia than in healthy individuals, and that negative symptoms were more pronounced in rs11030101-A homozygous patients.

One of the most studied BDNF polymorphisms, rs6265-T (V66M), which impairs activity-mediated BDNF secretion (Tsai, 2018), has been shown to be associated with a decrease in bilateral hippocampal volume (independent of patient’s sex and age) compared to the V66V homozygotes from the control group (Tate et al., 2012, 2015). Val/Met heterozygous individuals also showed the reduced lateral convexity of frontal cortex gray matter volumes (Tate et al., 2015). A study by Kranz et al. (2015) identified another missense variant rs769727156-T (G198D) in the BDNF gene in patients with schizophrenia-related psychosis (American population); however, its functional significance in predisposition to psychiatric and cognitive disorders remains to be established.

A number of other BDNF polymorphisms have been correlated with the manifestation of major depressive disorder—rs12273539-T (intr), rs11030103-G (intr), rs6265-T (V66M), rs28722151-G (intr), rs41282918-T (3′UTR), and rs11030101-T (intr) (in a Mexican American population) (Licinio et al., 2009), and Alzheimer’s disease (in a Japanese population)—rs56164415 (C270T) (Kunugi et al., 2001). In a Chinese population, the occurrence of the dinucleotide polymorphism (GT)_n_ in *BDNF* gene was found to correlate with early manifestation of schizophrenia and sensitivity to chlorpromazine treatment (Xu et al., 2008).

Due to the complexity of the nervous system structure and species-specific peculiarities of intracellular signaling, the functional significance of the BDNF polymorphisms and their putative mechanisms underlying the predisposition to mental disorders are yet to be unveiled (Gören, 2016).

The defects in the overall neurotrophic system (neurotrophic factors and their receptors) functioning may result from the altered signaling downstream the corresponding receptors. Polymorphisms and mutations in these receptor genes, impairing their expression or signaling activity may cause the dysregulated signaling. Overall, these defects may ultimately lead to neurological and psychiatric disorders. For example, in a Polish population the polymorphisms of the BDNF receptor—TrkB (*NTRK2* gene): rs10868235-T (intr) and rs1387923-G (3′UTR) was found to be associated with a higher risk of schizophrenia in men; rs1387923-A (3′UTR)—with a lower risk of schizophrenia, and the polymorphism rs1565445-A (intr)—with an increased risk of suicide in schizophrenia (Suchanek-Raif et al., 2020). It is worth noting that many of the described TrkB SNPs are associated with the predisposition to suicidal activity; therefore, it is suggested that *NTRK2* polymorphisms should be screened for if there was a history of suicidal ideation. The predisposition to schizophrenia (and other mental disorders) may be determined by a combination of several SNPs within several genes (so-called haplotypes), and their influence may differ between the sexes. For example, the ATAAT haplotype (5 SNP *NTRK2*) is associated with a reduced risk of schizophrenia in men, and the GTAGCC haplotype [5 SNP *NTRK2* and 1 rs6265-C (V64V) SNP of the *BDNF* gene] is associated with a reduced risk of it in women (Suchanek-Raif et al., 2020). Combinations of mutations in the ligand [*BDNF*, rs6265-T (V66M)] and receptor [*NTRK2*, rs1387923-G (3′UTR)] and rs2769605-T [(3′ downstream)] genes can significantly increase the risk of paranoid schizophrenia (in a Chinese population), even though the separate presence of such polymorphisms is not associated with this risk (Tsai, 2004, 2007; Hwang et al., 2006).

NGF (nerve growth factor, *NGF* gene), the first neurotrophin discovered (Levi-Montalcini, 1987), has been shown to stimulate the survival of sensory, sympathetic, cholinergic and mesencephalic dopaminergic neurons (Mobley et al., 1986; Gibbs, 1994; Hanaoka et al., 1994; Calamandrei and Alleva, 1995; Kang and Schumann, 1996) in the basal forebrain, hippocampus, cerebellum (Thoenen et al., 1987; Whittemore and Seiger, 1987), etc. The highest level of NGF expression is observed in hippocampus, prefrontal cortex (Large et al., 1986; Yan et al., 1997) and pituitary (Goedert et al., 1986; Hefti et al., 1986). Together with BDNF, NGF influences brain development by controlling cell death, fiber growth direction, dendrite morphology, and synapse formation (Thoenen, 1995). Moreover, NGF can induce the onset of mental and cognitive disorders by regulating the dopamine D2-receptor activity (Fiorentini et al., 2002), as well as by impairing oligodendrocyte formation/maturation and myelination (Chan et al., 2004). A number of *NGF* single-nucleotide genomic variants may be associated with the predisposition to cognitive and psychiatric disorders. Thus, it was shown that SNP variant rs6330-A (A35V) (Licinio et al., 2009; Zakharyan et al., 2014), as well as the haplotype rs12760036-C (intr)/rs4839435-A (intr) (in the Korean population) (Park et al., 2011) increase the risk of schizophrenia onset. The variant rs4565713-G (intr) is associated with susceptibility to schizophrenia manifestations in Russian and Tatar populations (Gareeva et al., 2015), and autism in an American population (Chen et al., 2006; Lu et al., 2013). *NGF* variants rs2856813-G (intr), rs6678788-T (intr), rs4529705-A (intr), rs6537860-A (intr), rs4332358-T (intr), and rs3811014-G (promoter) are associated with primary affective disorders (PAFDs) in women in the American population (Cui et al., 2011). *NGF* diplotype GG-TC in rs2856813 (intr) and rs6678788 (intr) has the strongest association with PAFDs in women in the same study (Cui et al., 2011), and the haplotype rs2254527-rs667878788-rs12760036 CCA (all introns) is associated with lower susceptibility to antidepressant therapy in major depressive disorder in a Chinese population (Yeh et al., 2015).

Abnormalities in NGF signaling caused by disruption of the structure of its receptors [TrkA (*NTRK1* gene) and p75 (*NGFR* gene)] or the signaling pathways involved, may also impair brain morphogenesis and lay the groundwork for the onset of mental and cognitive disorders. For example, some of the *Ntrk1* genomic variants [rs6336-T (H568Y) and rs4661063-A (intr)] were found to be associated with changes in the microstructure, such as the decreased myelination and impaired fasiculation of nerve fibers of the white matter of the cerebral cortex in rats (Su et al., 2021). The role of *NTRK1* in mental disorders is indirectly supported by the data that antipsychotics can reduce the level of TrkA autophosphorylation in the rat hippocampus (Terry et al., 2010), but the mechanism of the pro-psychotic activity of *NTRK1* remains to be established. Several lines of evidence show that increased TrkA activation may be associated with the manifestation of psychiatric disorders. For instance, the frequency of rs6336-T (H568Y) variant, which creates an additional potential site of TrkA autophosphorylation, correlates with the predisposition to schizophrenia in American and European populations (van Schijndel et al., 2009, 2011), although rs6336-T (H568Y) variant was identified as a protective factor in a number of experimental cohorts (van Schijndel et al., 2011). On the other hand, reduced TrkA-mediated signaling may also be a risk factor for psychiatric diseases. In particular, the rs556840308-A (G4S) NTRK1 variant (Kranz et al., 2015), that impairs TrkA exposure to the membrane, has been identified in patients with schizophrenia in an American population. Mutations in adaptor proteins mediating signal transduction from Trk receptors may also underlie the pathogenesis of psychiatric diseases. Another example is the rs748531715-C (H1085R) mutation in the *KIDINS220* gene, which encodes a scaffold protein and a substrate for Trk receptors, was shown to be associated with severe paranoid hallucinations and schizophrenia manifestation in adulthood (Encinas et al., 2000; Lasky-Su et al., 2008).

Another receptor for neurotrophins is the low-affinity nerve growth factor receptor p75NTR (*NGFR* gene) (Casaccia-Bonnefil et al., 1999; Bradshaw et al., 2015). This receptor predominantly binds the proforms of neurotrophins (as its affinity for mature neurotrophins is substantially lower) and has the capability to initiate apoptotic cascades in neural cells.

Recent evidence suggests that impaired signaling of neurotrophins through p75NTR may be associated with the predisposition to psychiatric and cognitive disorders. For example, Zhao et al. (2022) showed that *NGFR* SNPs rs2072446-T (S205L) and rs11466162-A (3′UTR) were associated with the incidence of schizophrenia, and *Ngfr* knockdown in mice was associated with schizophrenia-like social behavior abnormalities. A study of *NGFR* SNPs has revealed that rs11466155-T (G265G) and rs2072446-T (S205L) are associated with an increased risk of schizophrenia in an Armenian population. While rs734194-G (3′UTR) appeared to be a protective factor, SNPs rs734194, rs11466155 and rs2072446 had no significant effect on schizophrenia incidence (Park et al., 2011). At the same time, rs2072446-T (S205L) of the *NGFR* gene was also associated with an increased risk of Alzheimer’s disease and β-amyloid deposition (He et al., 2022). A Spanish population was shown to be amendable to addiction due to rs534561-G (intr) (Fernàndez-Castillo et al., 2013).

Neurotrophin-3 (NT-3, *NTF3* gene) has a similar structure as the other neurotrophins (NGF, BDNF) but has a different expression profile in the brain (Ernfors et al., 1990; Maisonpierre et al., 1990a). It is actively expressed in all brain regions of mouse embryos at postnatal day 4 (Thompson et al., 2014), including the hippocampus and neocortex. NT-3 plays an important role in brain maturation (Maisonpierre et al., 1990b; Nimgaonkar et al., 1995), but during adulthood its expression level decreases (Hattori and Nanko, 1995) and in the adult brain it is predominantly expressed in the hippocampus. Along with NGF and BNDF, NT-3 ensures the survival of dopaminergic neurons (Gall et al., 1992), sensitive neurons of the neural crest, and placodes. It is crucial for the survival of sympathetic and sensitive neurons, which are later supported by NGF and BDNF (Fariñas et al., 1994). Nt-3 is involved in the processes of neurogenesis, hippocampal plasticity, learning and memory (Shimazu et al., 2006). An increase in NT-3 levels in the serum of schizophrenia patients with predominantly negative symptoms has previously been reported but the underlying mechanism remains unclear (Arabska et al., 2018). Some polymorphisms of the *NTF3* gene turned out to be associated with the incidence of psychiatric and cognitive disorders, although they appear to have different significance in different populations. For example, a dinucleotide polymorphism (CA23) in the A3/147 bp *NTF3* promoter region was found to be associated with schizophrenia in a Japanese population (Nanko et al., 1994). However, these data have not been confirmed in other studies in American and European Caucasian populations (Dawson et al., 1995; Nimgaonkar et al., 1995; Jŏnsson et al., 1997; Virgos et al., 2001)—statistically significant correlations with schizophrenia predisposition have only been observed in females (Virgos et al., 2001). A statistically significant association between the frequency of *NTF3* gene SNP rs1805149-A (G76E) and the incidence of schizophrenia was found only in patients with the early manifestation (earlier than 25 years-old) and patients with a significant duration of the disease (more than 10 years) (Hattori and Nanko, 1995). For *NTF3* rs6489630-T (3′ downstream) a statistically significant association was established with lower intelligence scores in children with ADHD (Cho et al., 2010) and the predisposition to gambling disorder (haplotype with rs7956189-G [3′ downstream)] was found (Solé-Morata et al., 2022). Despite the importance of NT-3 in brain morphogenesis, the association of genomic variants of *NTF3* with predisposition to mental and cognitive disorders only in limited and narrow selections, which leaves the role of *NTF3* SNPs in psychiatric diseases in question.

As for the NT-3 TrkC receptor (*NTRK3* gene), some of its genomic variants were identified in patients with mental disorders. In particular, *NTRK3* SNPs rs12595249-C (intr), rs744994-T (promoter), and rs998636-G (promoter) was found to be associated with the predisposition to drug addiction in a Spanish population (Fernàndez-Castillo et al., 2013), C/T heterozygote in rs7180942 (intr) SNP—with the predisposition to eating disorders (Mercader et al., 2008), rs8037291-G (intr) variant—with the occurrence of ADHD (Smith et al., 2014) and rs1946698-C (intr)—with the predisposition to schizophrenia in a Russian population (Lu et al., 2013). However, functional significance of these SNPs in psychiatric and cognitive disorders remains to be elucidated.

Neurotrophin-4 (NT-4, *NTF4* gene) is expressed predominantly in dopaminergic, GABAergic neurons of the ventral midbrain and hippocampal neurons; it is also found in the thalamus, hypothalamus, medulla oblongata, cerebellum and pontine (Holtzman and Mobley, 1994). NT-4 promotes proliferation, differentiation, and survival of neural crest and placode sensory neurons (Ip et al., 1992), motoneurons, neurons of the basal forebrain and locus coeruleus (Friedman et al., 1993), and stimulates synaptic activity in hippocampal cultures *in vitro* (Friedman et al., 2002). Similarly to BDNF, TrkB and p75NTR function as receptors for NT-4. Since NT-4 participates in the processes of synaptogenesis, neuronal survival and neural plasticity, it may take part in the pathogenesis of psychiatric and cognitive disorders, like other neurotrophins. However, this assumption has not yet been experimentally confirmed—only an increase in NT-4 serum concentration was established in bipolar disorder (Loch et al., 2015), but not in schizophrenia (Skibinska et al., 2019). Kranz et al. (2015) also detected a genomic variant rs746640305-A (D162Y) in the *NTF4* gene in one of 48 patients with schizophrenia-related psychosis (American population); however, its functional significance was not experimentally verified. To date, there are no data on the association of certain genomic variants of *NTF4* with the predisposition to mental or cognitive disorders.

### 3.3 Glial neurotrophic factors and their receptors

Glial cell line-derived neurotrophic factor (GDNF, *GDNF* gene) is an ancestor to the family of glial neurotrophic factors, which includes neurturin (NRTN), artemin (ARTN), and persephin (PSPN) along with GDNF (Airaksinen and Saarma, 2002). GDNF is involved in the development, maintenance, survival, and function of dopaminergic and motor neurons in the mammalian nervous system (Lin et al., 1993; Airaksinen and Saarma, 2002). The *GDNF* gene is localized in the 5p12—p13.1 locus associated with schizophrenia (Moises et al., 2002; Bespalova et al., 2005).

Given its broad functional in the nervous system, GDNF has been considered to potentially contribute to the pathogenesis of mental diseases, including schizophrenia, known for dopaminergic system dysfunction. There is indirect evidence for such a link: particularly, application of drugs that cause schizophrenia-like symptoms elevates GDNF expression. For example, phencyclidine increases the GDNF expression, as well as of its receptors, GFRα1 and RET (Semba et al., 2004). Amphetamine administration elevates the endogenous GDNF expression in the nigrostriatal tract (Morrow et al., 2011; Valian et al., 2017), increases the concentration of dopamine in synapses, and elevates the schizophrenia susceptibility (Morrow et al., 2011; Li et al., 2014; Valian et al., 2017). It has also been demonstrated that a 2–3-fold increase in endogenous GDNF expression triggers schizophrenia-like conditions (including the expression of relevant genes and behavioral responses) and also imbalance the dopamine content between the prefrontal cortex and striatum (Ross et al., 2008; Liao et al., 2012; Wearne and Cornish, 2018).

At the same time, only a small number of studies have been devoted to investigating the relationship between the occurrence of GDNF genomic variants and the predisposition to psychiatric disorders (Lee et al., 2001; Ma et al., 2013), with no reliable data obtained to definitively confirm such correlations. For example, no reliable associations were found between the frequency of trinucleotide repeats (AGG)_n_ (and a number of GDNF missense mutations) and the incidence of psychiatric disorders in a Japanese population. In contrast, a polymorphism containing 15 or more AGG repeats was identified as a protective factor in an Italian population (Michelato et al., 2004). Studies in English and Chinese populations also revealed no functional significance of GDNF polymorphisms: (AGG)_n_, rs2910709-C/G/T (3′ downstream), rs2973050-C/G/T (intr), rs884344-C (intr), rs2910702-A/T (intr), rs2216710-T (intr), rs3812047-G/T (intr), etc. in the development of mental disorders (Kotyuk et al., 2013).

GDNF signaling is mediated by the heterotetrameric receptor complex, consisting of GFRα1 (GDNF family receptor alpha-1) and RET (Receptor Tyrosine Kinase) (Airaksinen and Saarma, 2002). Abnormalities in the structure and signaling of GFRα1 (*GFRA1* gene) or RET (*RET* gene), caused by genomic variants or mutations within these genes, may also underlie various mental and cognitive disorders. For example, an association was determined between the incidence of rs11197557-T (intr) SNP in the *GFRA1* gene and predisposition to schizophrenia (Souza et al., 2010). Interestingly, this polymorphism is localized in the locus of chromosome 10, which is associated with schizophrenia predisposition.

Impaired expression of artemin (*ARTN* gene) results in altered nervous system development (*Artn* knockout) in a murine model (Honma et al., 2002) or in the increased excitability of striatal dopamine neurons in humans (Elitt et al., 2006, 2008). rs11242417-G (intr) polymorphism in the artemin receptor GFRα3 gene (*GFRA3*) turned out to be associated with the schizophrenia predisposition (Souza et al., 2010).

No genomic variants for RET (the signal-transducing subunit of heterotetrameric neurotrophin receptor from the GDNF family) have been identified in association with the susceptibility to psychiatric disorders. At the same time, a complete deletion of RET activity coinciding with schizophrenia manifestation in a European population have been elucidated (Glessner et al., 2010).

### 3.4 Cytokines and their receptors. IL-6

Ciliary neurotrophic factor (CNTF, *CNTF* gene), a member of the neuropoietic cytokine family, is expressed in the brain and spinal cord (Pasquin et al., 2015). CNTF supports the survival and proliferation of hippocampal neurons, medial septal neurons, and appears to be involved in development of the central nervous system (Sakai et al., 1997).

The role of CNTF in the pathogenesis of psychiatric disorders has not been elucidated. Most studies indicate that there is no correlation between the incidence of psychiatric disorders and the prevalence of known CNTF genomic variants (Sakai et al., 1997; Tanaka et al., 1998; Benkovits et al., 2016). For rs1800169 (FS63Stop) a correlation with the incidence of schizoaffective disorder and a better response to iloperidone (antipsychotic) therapy was reported (Lavedan et al., 2008; Okahisa et al., 2010). A number of studies demonstrate that impaired CNTF signaling caused by mutations in the genes of the receptor complex CNTFR*gp130 (*CNTFR* and *IL6ST* genes, respectively) may contribute to mental disorders. Moreover, some SNPs in the *CNTFR* gene have been associated with the predisposition to drug addiction [rs7036351-T (promoter), Spanish population (Fernàndez-Castillo et al., 2013)], gambling addiction rs3763614-G (intr) (Solé-Morata et al., 2022), and ADHD rs7036351-C (promoter), rs3763613-G (intr) in a Spanish population (Ribasés et al., 2008), rs10758268-T (intr) and rs7044318-T (intr) in European and Israely populations (Smith et al., 2014).

The signaling subunit of the receptor complex for neuropoietic cytokines gp130 (*IL6ST* gene), provides signaling upon binding of the cytokines CNTF, LIF, OSM and IL-6 to the corresponding receptor subunit. This signaling cascade supports proliferation of neural progenitors of the hippocampus, forebrain and spinal cord and prevents their premature differentiation (Kummer et al., 2021). Increased *IL6ST* expression is associated with neuroinflammation and impaired brain development, which can contribute to the manifestation of autism (Google Patents, 2007). Point mutations (rs1580809257-A (N404Y), rs1381682599-G (A517P), rs1580801731-A (P498L), VAR_086953, VAR_086954, VAR_086955) (Schwerd et al., 2017; Shahin et al., 2019; The UniProt Consortium, 2023c) are also known to block gp130-dependent signaling of the following cytokines: IL-6, IL-11, IL-27, OSM and LIF, or, conversely, to induce constitutive activation of gp130 (VAR_086950) (Materna-Kiryluk et al., 2021), resulting in immunodeficiency and impaired brain tissue development and causing autism and mental retardation [e.g., Hyper-IgE recurrent infection syndrome 4 (HIES)] (Crama et al., 2004; Shahin et al., 2019).

One of the gp130-dependent neuropoietic cytokines IL-6 (*IL6* gene) plays an important role in the maintenance of neural progenitors and differentiation of neurons in the central and peripheral nervous system. Under certain conditions, IL-6 exhibits neuroprotective activity towards dopaminergic and cholinergic neurons, regulates neuronal electrical activity and sleep (Tartter et al., 2015; Kummer et al., 2021). However, increased IL-6 expression inhibits neurogenesis in the hippocampus (Tartter et al., 2015), can activate neuroinflammation and lead to autoimmune brain damage (Tartter et al., 2015; Kummer et al., 2021). Excessive IL-6 can also cause depression, presumably via activating the hypothalamic-pituitary-adrenal axis or via altering the neurotransmitter metabolism (Ting et al., 2020). Due to its ability to be both a cytokine and a neuroprotective molecule, IL-6 is one of the possible intersections between the developmental and inflammatory hypothesis of psychiatric disorders onset.

Two genomic variants of *IL6* gene are most frequently associated with mental and cognitive disorders: rs1800795 and rs1800796. Thus, the *IL6* rs1800795-C (promoter) genomic variant is associated with the predisposition to depression onset in Australian (Tartter et al., 2015) and Spanish populations (Udina et al., 2013), and also correlates with increased sensitivity to pain and stress perception in a Hungarian population of depressed patients (Kovacs et al., 2016). In contrast, the *IL6* rs1800795-G (promoter) genomic variant promotes neuroprotection and preservation of hippocampus (Baune et al., 2012). Homozygous carriers of the rs1800796-C (promoter) variant in a Chinese population are amendable to depression and chronic schizophrenia (Lu et al., 2023).

IL-6 signaling disorders caused by mutations in the ligand-binding subunit of IL6R (*IL6R* gene) may also be the cause of psychiatric and cognitive disorders. For example, *IL6R* genomic variants rs2228145-C (D358A) and rs4537545-T (intr) are associated with the risk of psychosis/schizophrenia and severe depression in a European population (Kapelski et al., 2015; Khandaker et al., 2018). The *IL6R* rs57569414 (intr) polymorphism has been shown to be associated with the depression severity and therapy resistance in a Spanish population (Draganov et al., 2019).

As described above, cell-to-cell and cell-to-matrix interactions as well as guidance signals also play an important role in brain tissue morphogenesis. There is a body of data suggesting that impaired expression or dysfunction of these types of molecules can lead to dystopia of neuronal cells, aberrant connections and, as a consequence, can launch the pathogenesis of mental disorders.

### 3.5 Growth factors and their receptors

One of the key cell-to-cell interaction axes for brain maturation is the neuregulin/ErbB signaling cascade (genes *NRG1*-*NRG4* and *ERBB2*-*ERBB4*, respectively), which mediates the interaction of neurons with glial cells, regulates migration of GABA-producing neurons, synapse formation and myelination, development and maintenance of the function of the hippocampus, cerebellum, and right anterior cingulate (Moradkhani et al., 2023). Experimental disruption of this axis reduced the density of neuron dendritic spines and disrupted the glutamatergic signaling in the murine brain (Barros et al., 2009). The *NRG1* rs35753505-C (promoter) variant has been shown to be associated with the schizophrenia predisposition in Iranian and Indian populations (Rajasekaran et al., 2020; Moradkhani et al., 2023). The variant rs6994992-T (promoter), which induces the expression of a special NRG1 isoform, correlates with an increased risk of psychosis, and occurrence of “more unusual thoughts in T-carriers than in C-carriers (C/T and C/C) during conflict-related interactions” (Kéri et al., 2009). Genomic variants rs1937970-A (intr) and rs677221-G (intr) in the *NRG3* gene are associated with an increased incidence of schizophrenia in a Han Chinese population (Wang et al., 2008), and a number of *NRG3* polymorphisms, such as rs10883934-C (intr), rs1896506-A (intr) and others, correlate with the predisposition to nicotine addiction in an American population (Turner et al., 2014).

ERBB2-4 receptor genes, epidermal growth factor family receptors, mediate the perception of signals from Neuregulins 1–4 and regulate the processes of cell proliferation, survival, migration, and differentiation (mainly in tissues of ectodermal origin) both in ontogeny and in the adult organism. Alterations in ERBB expression or signaling are associated with malformations and congenital malignant neoplasms of organs and tissues (The UniProt Consortium, 2023d,e,f). Prats et al. (2022) linked the disruption of the Neuregulin/ERBB axis genes to the defects in white matter formation in the brain of European individuals, manifested in schizophrenia-spectrum and autism-spectrum disorders. The *ERBB4* gene has been found to be associated with predisposition to schizophrenia: rs707284-G (intr), rs839523-G (intr), and rs7598440-A (intr)—in a Jewish population (Silberberg et al., 2006), rs2289086-T (intr)—in a Chinese Han population (Lu et al., 2010), and rs3748962-G (V1065V)—in an African American population (Nicodemus et al., 2006).

### 3.6 Guidance receptors and their ligands

Ephrins are related to the family of guidance molecules involved in axon growth. Ephrins and their receptors are membrane-anchored molecules, and their interaction triggers signaling in both interacting cells (Taylor et al., 2017). Type A ephrins (*EFNA1*-*EFNA5* genes) and type B ephrins (*EFNB1*–*EFNB3* genes) have homologous extracellular domains but differ in the way they are anchored to the plasma membrane: EFNAs are anchored via a GPI-moiety, while EFNBs are transmembrane proteins (Blits-Huizinga et al., 2004). Binding of ephrins to their receptors activates PI3K/Akt signaling cascade and Src family kinases (for example, Grb4) in ligand (EFNA1-EFNA5, EFNB1-EFNB3)-expressing cells (so called, reverse signaling) (Yang et al., 2018). Ephrin receptors are tyrosine kinases (Blits-Huizinga et al., 2004) and include two subfamilies: type A (*EPHA1*–*EPHA8* and *EPHA10* genes) and type B (*EPHB1*–*EPHB4* and *EPHB6* genes) (Taylor et al., 2017). In the cells, expressing ephrin receptors, interaction with an ephrin (forward signaling) triggers activation of Ras/ERK, PI3K/Akt and JAK/STAT3 as well as Src family kinases signaling cascades.

Ephrins and their receptors are expressed in the brain at all stages of nervous system development (Cramer and Miko, 2016). They prevent cell intermingling within the developing brain (Taylor et al., 2017), regulate the radial neuron and tangential interneuron migration (Cramer and Miko, 2016), axonal outgrowth and pathfinding, topographic mapping, axon fasciculation, synapse maturation and vascular formation in the developing nervous system (Yang et al., 2018).

Dysregulation of ephrin or their receptor expression, as well as the downstream signal transduction molecules can potentially disrupt the architectonics of the developing brain. For instance, it has been shown that missense mutations in the *EFNB1* gene (VAR_023127 (P27R), rs104894803-A (W37Stop), rs104894801-T (P54L), etc.) are associated with Craniofrontonasal syndrome (characterized by encephalocele, mental retardation) (The UniProt Consortium, 2023g), while mutations in the ephrin receptor genes: *EPHA4* [rs155356868456-T (A748T)], *EPHA5* [rs200932017-C (D348G)], and *EPHB4* [rs1584666961-T (W130Stop), rs1562973541-CA (V211Missing), rs1484547615-A (G375Stop), etc.] are associated with the atypical cerebral palsy (The UniProt Consortium, 2023h,i,j), astrocytoma (The UniProt Consortium, 2023i), and Capillary malformation-arteriovenous malformation 2 syndrome (capillary malformation, hydrocephalus, headaches, epilepsy) (The UniProt Consortium, 2023j), respectively.

The ephrin *EFNB2* gene, being located at chromosomal locus 13q33, is associated with schizophrenia (Zhang et al., 2010; Su et al., 2016) and is a potential candidate gene implicating its contribution to mental disorders. Zhang et al. (2010) have demonstrated that the *EFNB2* genomic variant rs9520087-T (3′UTR) and haplotype rs9520087/rs11069646(intr)/rs8000078(intr)-TTC are significantly associated with the predisposition to schizophrenia in a Chinese Han population. Analysis of the *EPHB1* rs11918092 (promoter) and *EPHB2* rs9520087 (3’UTR) genomic variants in Chinese Zhuang and Han populations reveal no statistically significant association with schizophrenia susceptibility. However, an association of rs11918092 variant with the severity of positive symptoms of schizophrenia was found in a Han Chinese population, while the occurrence of rs9520087 variant correlated with the severity of negative symptoms of schizophrenia in a Zhuang Chinese population (Su et al., 2016). A study of monozygotic twin pairs with schizophrenia (a Canadian population) revealed a 25 kbp deletion at loci 3p11.2–3p11.1 that overlapped with the ephrin receptor gene *EPHA3*, but it was not determined, whether the *EPHA3* gene disruption was a direct cause of schizophrenia (Castellani et al., 2014). Two studies addressed the association of genomic variants rs140725416-C (F151S), rs150028142-A (D375N), rs145366861-A (D577N), rs149160192-T (R637C), rs143309901-T (R905C), and rs56186270-T (T981M) in the *EPHB1* gene (Kushima et al., 2012) and rs727229-G (intr) in the *EPHA6* gene (Ikeda et al., 2010) with the predisposition to schizophrenia in a Japanese population yielding no significant relation. However, the *Epha6* gene is one of those genes with an increased expression in the prefrontal cortex of mice treated with risperidone (belonging to neuroleptics) (Ikeda et al., 2010).

Semaphorins are another family of guidance molecules involved in axon pathfining, neural tissue development, and regeneration. About 30 semaphorins are known, represented by 8 classes, 5 of which found in vertebrates. In the human nervous system, there are secreted (gene *SEMA3*), transmembrane (genes *SEMA4*, *SEMA5* and *SEMA6*) and GPI-anchored (gene *SEMA7*) semaphorins. In the nervous system, semaphorins act as repellents or attractants for growing neurites (depending on the proteoglycans co-expressed on the cell surface). Semaphorins regulate neurite pruning (Uesaka et al., 2014), synapse formation (Koropouli and Kolodkin, 2014), and take part in stabilization of the perineuronal network (Pérez et al., 2021).

Neuropilin (*NRP1* and *NRP2* genes) and plexin (*PLXNA1*–*A4*, *PLXNB1*–*B3*, *PLXNC1*, *PLXND1* genes) protein families are receptors for semaphorins (Wannemacher et al., 2011). The signaling cascades triggered (RhoA/ROCK) or inhibited (ras/ERK, PI3K/AKT, Src kinases) by semaphorin-receptor interaction and the effects observed (cytoskeletal rearrangements, decreased/increased apoptosis and cell migration) are determined by the type of semaphorin-receptor pair, cell type, and context (Law and Lee, 2012; Butti et al., 2018; Jiao et al., 2021). Reverse signaling has been shown for the transmembrane forms of semaphorins (SEMA4, SEMA5 and SEMA6) (Perez-Branguli et al., 2016; Gurrapu et al., 2019).

There is sufficient evidence that the expression level of semaphorins and their receptors in different brain regions is altered in cognitive and psychiatric disorders (Gilabert-Juan et al., 2015; Carulli et al., 2021). A number of animal models support the assumption that semaphorins and their receptors may have an impact on the susceptibility to psychiatric and cognitive disorders. Thus, *PlxnA2*−/− mice exhibit symptoms of Schizophrenia, *Sema3F*−/− mice exhibit symptoms of anxiety and autism, and *Sema5A*−/− mice exhibit symptoms of autism (Carulli et al., 2021). Moreover, there are genomic variants of semaphorins and their receptors associated with the predisposition to psychiatric and cognitive disorders, including, autism [*SEMA6B* rs1977288029-G (Y385H) (The UniProt Consortium, 2023k), *PLXNA3* rs149367480-A (E72K) (The UniProt Consortium, 2023m)], schizophrenia [*PLXNA2*: rs752016-T (intr) and rs1327175-C (intr) in an American population; *PLXNB3*: rs2266879-A (V598I), rs6643791-C (E1156D) and rs146832392-A (V1596E) in a German population], childhood-onset schizophrenia [*PLXNA3* rs200042650-A (R616Q) (The UniProt Consortium, 2023m)], and intellectual disability [*PLXNA1* rs576960383-A (P2Q) (The UniProt Consortium, 2023l), etc.]. More detailed information on the known genomic variants of semaphorines and plexines is provided in the Supplementary Table S1.

Netrins (*NET1*, *NET3*, *NET4*, *NTNG1*, *NTNG2* genes) and SLIT proteins (*SLIT1*–*3* genes) are also key guidance factors for sprouting neurites in the developing brain that contribute to the proper wiring of its divisions (Gonda et al., 2020; Yamagishi et al., 2021). Netrin receptors include the following protein families: UNC5 (Unc-5 Netrin Receptor, *UNC5A*–*D* genes) and DCC (Deleted In Colorectal Carcinoma, *DCC* gene). Homodimerization of DCCs by a netrin molecule triggers Ras/ERK, PI3K/Akt, PLCγ and RAC1/PAK1 signaling cascades, which stimulate cell survival, promote cell adhesion to matrix, migration and neurite sprouting (via actin assembly) (Zhang et al., 2021). Heterodimerization of DCC-UNC5 by netrin molecules triggers the RhoA/ROCK1/2 signaling pathway, which destabilizes the cytoskeleton in the axon growth cone that in its turn, leads to neurite retraction and/or synapse disruption (Mulherkar and Tolias, 2020). ROBO (Roundabout, *ROBO1-4* genes) are the receptors for SLIT proteins. Upon ligand binding, ROBOs activate the RhoA/ROCK1/2 signaling pathway and inhibit β-catenin activation, resulting in cell de-adhesion, neurite retraction (Gonda et al., 2020).

A number of studies have confirmed the importance of netrins, SLIT proteins and their receptors in neural progenitor migration, establishment of inter-neuronal connections, as well as in the maintenance of cognitive functions of the brain.

Further, it was shown that missense mutations rs1567750186-C (C601R) and rs1567749982 (I518missing) in the *NTN1* gene are associated with the Mirror movements 4 condition (The UniProt Consortium, 2023n). Genomic variants rs1589440982-C (C81Y), rs1589441229-G (W107G) and others in the *NTNG2* gene are associated with the NEDBBASH condition (Neurodevelopmental disorder with behavioral abnormalities, absent speech, and hypotonia) (The UniProt Consortium, 2023o). SNPs rs754914260-T (R275Stop), rs7970445519-A (S126Stop) and rs1180126622-T (R215Stop) in the *DCC* gene (netrin receptor) are associated with the Mirror movements 1 condition (The UniProt Consortium, 2023p). Mutations in the *ROBO1* (SLIT protein receptor) gene are associated with the Tetralogy of Fallot condition [increased risk of anxiety, depressive, bipolar, and sleep disorders) (rs1017845770-A (R119Stop)] (Hsu et al., 2021; The UniProt Consortium, 2023q) and intellectual disability (rs765740846-A (D1042V), rs1704874919-T (P1267Q), rs778040289-A (R1420L)) (The UniProt Consortium, 2023q).

In addition to the described neurotrophic and guidance molecules, a huge number of other molecules were shown to contribute to the correct development and the brain tissue architecture. These include but are not limited to intercellular adhesion molecules (IgCAMs, cadherins, selectins), matrix proteins (collagens, laminins, fibronectin, chondroitin sulfate proteoglycans, reelin, tenascin, etc.), and their receptors (integrins, syndecans, discoidin domain receptors, IgCAMs, leukocyte common antigen related receptors, Nogo receptors, LDLRs, etc.), as well as cytoskeletal proteins (doublecortin, tubulins, f-actin, filamin A, molecular motors, etc.), molecules mediating intracellular signal transduction (G-proteins, small GTPases, protein kinases, phospholipases, etc.) or cell-cycle progression regulators (e.g., Cyclin-dependent kinase 5) (Chae et al., 1997; Gilmore et al., 1998). Some of these proteins provide guidance for their migrating neuronal progenitors and sprouting neurites; the others control the adequate and functional connections being formed between individual neurons and brain parts or provide for appropriate functioning of the molecular systems involved (Kwon et al., 2000; Dent et al., 2011; Ana and Elisa, 2019). However, the detailed overview of these groups beyond the scope of this article. However, literature data suggest that critical mutations in these genes, or their impaired spatiotemporal or quantitative expression, can disrupt default migration of neural progenitors and growth cone guidance, and thus providing the mechanistic underpinning for the onset of mental disorder.

Thus, we have previously demonstrated that overexpression of a guidance molecule Plaur (urokinase-type plasminogen activator receptor) in radially migrating neurons in the developing cortex of E15.5 murine embryos, enhances their migration into the cortical plate compared to the control (Shmakova et al., 2021). On the contrary, Plaur inactivation impairs the migration of neurons (e.g., GABA interneurons) and axonal growth (Powell et al., 2003). It has also been shown that a number of single nucleotide substitutions in these genes can be associated with predisposition to psychiatric and cognitive disorders: VAR_087507 (H150Y) in the *CDH2* gene is associated with the ADHD8 (Halperin et al., 2021), and rs2013111940-T (V162D), rs1599017933-T (D353N), rs1599011050-T (D597N) and the others within the same gene are associated with ACOGS (Agenesis of corpus callosum, cardiac, ocular, and genital syndrome) (Reis et al., 2020). Missense-mutations rs1753211537-A (E39Stop), rs759794990-A (S147I), and rs375346212-A (R839Stop) in the *PCDH12* gene were associated with the Diencephalic-mesencephalic junction dysplasia syndrome 1 (Nicolas et al., 2017; The UniProt Consortium, 2023s), while mutations in the *CDH13* gene: rs11647188-G (intr), rs6565113-C (intr) and rs11150556-C (intr) - turned out to be linked to ADHD (Lasky-Su et al., 2008; Salatino-Oliveira et al., 2015) and autism spectrum disorders (Sanders et al., 2011, 2015).

The accumulated data suggest a significant contribution of adhesion and guidance molecules to the process of brain development, its structural and functional homeostasis, which largely determines the state and stability of an individual’s mental health. Severe alterations in brain architectonics inflicted by dysfunction of adhesion and guidance molecules may provide the solid foundation for mental and cognitive disorders. Detailed information on the known genomic variants of adhesion and guidance molecules associated with psychiatric disorders is provided in Supplementary Table S1.

## 4 Conclusion

The processes of brain development and maturation involve a whole roster of cellular participants, whose activity is orchestrated by a large number of soluble molecules and various cell–cell contacts. Dysfunction of these signals may alter the morphogenesis of brain divisions and their wiring, which in turn may be a predisposing factor for the onset of mental and cognitive disorders. The complexity of brain architecture and development makes many variants of brain malformations and wiring disorders possible, which can manifest in a range of mental and cognitive disorders.

These disorders stem from dysregulated expression of key morphogenic factors and mutations (point or chromosomal) within their coding and/or regulatory regions. Some genomic abnormalities and rearrangements are so deleterious that their mere presence almost inevitably impairs neurodevelopment and leads to mental illness. However, most genomic variants manifested in the altered concentration or activity of the individual signaling molecules usually do not entail such severe consequences. The impact of a mutant gene on the pathogenesis of mental disease is determined by its uniqueness, the possibility of its functional substitution (Agerman and Ernfors, 2003), the presence of concomitant mutations (Benzel et al., 2007), environmental conditions, and individual characteristics of the organism (including population peculiarities) (Kong et al., 2009).

The majority of genomic variants associated with psychiatric and cognitive disorders are typically identified in the noncoding regions of genes. This might be accounted for the role these noncoding regions play in regulating the expression of key morphogenic genes. Otherwise, changes in the certain splice variants of coding and non-coding RNAs (their formation or activity) may also be the reason. Non-coding RNAs, capable of integrating signaling cascades, controlling gene expression, or mediating intercellular communication may also play a significant role in brain morphogenesis and provide the material basis for its normal or aberrant mental activity. The nuanced study represents a challenge for further explorations.

Most mental and cognitive disorders exhibit a complex nature and possess an underlying genetic background. However, the likelihood and onset age of their manifestation, as well as the severity of the disease, are heavily affected by environmental or non-genetic factors such as stress, environmental conditions, substance abuse, medications, and more. Understanding the pathogenetic mechanisms of mental illness, including the genes involved, provide an ability to predict its future manifestation and opportunity to intervene in its onset or progression. This intervention can involve mitigating the impact of non-genetic factors or implementing prophylactic therapy, such as antidepressants or anxiolytics, tailored to the individual situation (Sokolov, 1998). Furthermore, this understanding facilitates the identification of new pharmacological targets and promising therapeutic approaches, such as small molecules, growth factors, or non-coding RNAs. For instance, antidepressants that enhance neurotrophic factor production or anxiolytics that suppress neuroinflammation can be prescribed, combining the effects of symptomatic and pathogenetic therapies (Nibuya et al., 1995; Zheng et al., 2023). However, elucidating the contribution and functional significance of specific genes and their variants in neural tissue development warrants further investigation using cellular or animal models, which is now becoming increasingly accessible due to the possibilities of modern genetic and cellular technologies (Białoń and Wąsik, 2022; Dougnon and Matsui, 2022; Karagyaur et al., 2022).

## Author contributions

AP: Visualization, Writing – original draft. KB: Visualization, Writing – original draft. KR: Writing – review & editing. SD: Visualization, Writing – original draft. EN: Conceptualization, Project administration, Supervision, Writing – review & editing. MI: Writing – original draft. ES: Writing – review & editing. DS: Writing – original draft. VT: Conceptualization, Project administration, Supervision, Writing – review & editing. MK: Conceptualization, Funding acquisition, Project administration, Supervision, Visualization, Writing – original draft, Writing – review & editing.

## References

[ref1] Agerman K. Ernfors P. (2003). Differential influence of BDNF and NT3 on the expression of calcium binding proteins and neuropeptide Y *in vivo*. Neuroreport 14, 2183–2187. doi: 10.1097/00001756-200312020-00010, PMID: 14625444

[ref2] Airaksinen M. S. Saarma M. (2002). The GDNF family: signalling, biological functions and therapeutic value. Nat. Rev. Neurosci. 3, 383–394. doi: 10.1038/nrn812, PMID: 11988777

[ref3] Allaway K. C. Gabitto M. I. Wapinski O. Saldi G. Wang C. Y. Bandler R. C.. (2021). Genetic and epigenetic coordination of cortical interneuron development. Nature 597, 693–697. doi: 10.1038/s41586-021-03933-1, PMID: 34552240 PMC9316417

[ref4] Ana V. A. Elisa T. (2019). “Cell-cell and cell-matrix interactions during axons guidance” in The neurons—dendrites and axons. eds. Abreu G. E. A. Aguilar M. E. H. (United Kingdom: IntechOpen), 128.

[ref5] Angoa-Pérez M. Anneken J. H. Kuhn D. M. (2017). The role of brain-derived neurotrophic factor in the pathophysiology of psychiatric and neurological disorders. J. Psychiatry Psychiatr. Disord. 1, 252–269. doi: 10.26502/jppd.2572-519X0024 35098038 PMC8793768

[ref6] Anton E. S. Marchionni M. A. Lee K. F. Rakic P. (1997). Role of GGF/neuregulin signaling in interactions between migrating neurons and radial glia in the developing cerebral cortex. Development 124, 3501–3510. doi: 10.1242/dev.124.18.3501, PMID: 9342043

[ref7] Arabska J. Łucka A. Strzelecki D. Wysokiński A. (2018). In schizophrenia serum level of neurotrophin-3 (NT-3) is increased only if depressive symptoms are present. Neurosci. Lett. 684, 152–155. doi: 10.1016/j.neulet.2018.08.005, PMID: 30098383

[ref8] Arévalo J. C. Wu S. H. Takahashi T. Zhang H. Yu T. Yano H.. (2010). The ARMS/Kidins220 scaffold protein modulates synaptic transmission. Mol. Cell. Neurosci. 45, 92–100. doi: 10.1016/j.mcn.2010.06.002, PMID: 20547223 PMC2923264

[ref9] Arévalo J. C. Yano H. Teng K. K. Chao M. V. (2004). A unique pathway for sustained neurotrophin signaling through an ankyrin-rich membrane-spanning protein. EMBO J. 23, 2358–2368. doi: 10.1038/sj.emboj.7600253, PMID: 15167895 PMC423292

[ref10] Bachiller D. Klingensmith J. Shneyder N. Tran U. Anderson R. Rossant J.. (2003). The role of chordin/Bmp signals in mammalian pharyngeal development and DiGeorge syndrome. Development 130, 3567–3578. doi: 10.1242/dev.00581, PMID: 12810603

[ref11] Barros C. S. Calabrese B. Chamero P. Roberts A. J. Korzus E. Lloyd K.. (2009). Impaired maturation of dendritic spines without disorganization of cortical cell layers in mice lacking NRG1/ErbB signaling in the central nervous system. Proc. Natl. Acad. Sci. U.S.A. 106, 4507–4512. doi: 10.1073/pnas.0900355106, PMID: 19240213 PMC2657442

[ref12] Barry D. S. Pakan J. M. O’Keeffe G. W. McDermott K. W. (2013). The spatial and temporal arrangement of the radial glial scaffold suggests a role in axon tract formation in the developing spinal cord. J. Anat. 222, 203–213. doi: 10.1111/joa.12006, PMID: 23121514 PMC3632225

[ref13] Bathina S. Das U. N. (2015). Brain-derived neurotrophic factor and its clinical implications. Arch. Med. Sci. 11, 1164–1178. doi: 10.5114/aoms.2015.56342, PMID: 26788077 PMC4697050

[ref14] Baune B. T. Konrad C. Grotegerd D. Suslow T. Birosova E. Ohrmann P.. (2012). Interleukin-6 gene (IL-6): a possible role in brain morphology in the healthy adult brain. J. Neuroinflammation 9:125. doi: 10.1186/1742-2094-9-125, PMID: 22695063 PMC3464888

[ref15] Benkovits J. Magyarosi S. Pulay A. J. Makkos Z. Egerhazi A. Balogh N.. (2016). Investigation of CNTF, COMT, DDR1, DISC1, DRD2, DRD3, and DTNBP1 candidate genes in schizophrenia: results from the Hungarian SCHIZOBANK Consortium. Neuropsychopharmacol. Hung. 18, 181–187. PMID: 28259861

[ref16] Benzel I. Bansal A. Browning B. L. Galwey N. W. Maycox P. R. McGinnis R.. (2007). Interactions among genes in the ErbB-neuregulin signalling network are associated with increased susceptibility to schizophrenia. Behav. Brain Funct. 3:31. doi: 10.1186/1744-9081-3-31, PMID: 17598910 PMC1934910

[ref17] Bersani G. Iannitelli A. Fiore M. Angelucci F. Aloe L. (2000). Data and hypotheses on the role of nerve growth factor and other neurotrophins in psychiatric disorders. Med. Hypotheses 55, 199–207. doi: 10.1054/mehy.1999.1044, PMID: 10985909

[ref18] Bespalova I. N. Angelo G. W. Durner M. Smith C. J. Siever L. J. Buxbaum J. D.. (2005). Fine mapping of the 5p13 locus linked to schizophrenia and schizotypal personality disorder in a Puerto Rican family. Psychiatr. Genet. 15, 205–210. doi: 10.1097/00041444-200509000-00012, PMID: 16094256

[ref19] Białoń M. Wąsik A. (2022). Advantages and limitations of animal schizophrenia models. Int. J. Mol. Sci. 23:5968. doi: 10.3390/ijms23115968, PMID: 35682647 PMC9181262

[ref20] Bishop K. M. Rubenstein J. L. O’Leary D. D. (2002). Distinct actions of Emx1, Emx2, and Pax6 in regulating the specification of areas in the developing neocortex. J. Neurosci. 22, 7627–7638. doi: 10.1523/JNEUROSCI.22-17-07627.2002, PMID: 12196586 PMC6757966

[ref21] Blits-Huizinga C. T. Nelersa C. M. Malhotra A. Liebl D. J. (2004). Ephrins and their receptors: binding versus biology. IUBMB Life 56, 257–265. doi: 10.1080/15216540412331270076, PMID: 15370889

[ref22] Bortone D. Polleux F. (2009). KCC2 expression promotes the termination of cortical interneuron migration in a voltage-sensitive calcium-dependent manner. Neuron 62, 53–71. doi: 10.1016/j.neuron.2009.01.034, PMID: 19376067 PMC3314167

[ref23] Bradshaw R. A. Pundavela J. Biarc J. Chalkley R. J. Burlingame A. L. Hondermarck H. (2015). NGF and ProNGF: regulation of neuronal and neoplastic responses through receptor signaling. Adv. Biol. Regul. 58, 16–27. doi: 10.1016/j.jbior.2014.11.003, PMID: 25491371 PMC4426037

[ref24] Buss R. R. Oppenheim R. W. (2004). Role of programmed cell death in normal neuronal development and function. Anat. Sci. Int. 79, 191–197. doi: 10.1111/j.1447-073x.2004.00088.x, PMID: 15633457

[ref25] Butt S. J. B. Cobos I. Golden J. Kessaris N. Pachnis V. Anderson S. (2007). Transcriptional regulation of cortical interneuron development. J. Neurosci. 27, 11847–11850. doi: 10.1523/JNEUROSCI.3525-07.2007, PMID: 17978022 PMC6673372

[ref26] Butti R. Kumar T. V. Nimma R. Kundu G. C. (2018). Impact of semaphorin expression on prognostic characteristics in breast cancer. Breast Cancer 10, 79–88. doi: 10.2147/BCTT.S135753, PMID: 29910635 PMC5987790

[ref27] Calamandrei G., and, Alleva E. (1995). Neuronal growth factors, neurotrophins and memory deficiency. Behav. Brain Res. 66, 129–132. doi: 10.1016/0166-4328(94)00133-Z, PMID: 7755882

[ref28] Carulli D. de Winter F. Verhaagen J. (2021). Semaphorins in adult nervous system plasticity and disease. Front. Synaptic Neurosci. 13:672891. doi: 10.3389/fnsyn.2021.672891, PMID: 34045951 PMC8148045

[ref29] Casaccia-Bonnefil P. Gu C. Chao M. V. (1999). Neurotrophins in cell survival/death decisions. Adv. Exp. Med. Biol. 1999, 275–282. doi: 10.1007/978-1-4615-4685-6_22 10635036

[ref30] Castellani C. A. Awamleh Z. Melka M. G. O’Reilly R. L. Singh S. M. (2014). Copy number variation distribution in six monozygotic twin pairs discordant for schizophrenia. Twin Res. Hum. Genet. 17, 108–120. doi: 10.1017/thg.2014.6, PMID: 24556202

[ref31] Chae T. Kwon Y. T. Bronson R. Dikkes P. Li E. Tsai L. H. (1997). Mice lacking p35, a neuronal specific activator of Cdk5, display cortical lamination defects, seizures, and adult lethality. Neuron 18, 29–42. doi: 10.1016/S0896-6273(01)80044-1, PMID: 9010203

[ref32] Chai X. Frotscher M. (2016). How does reelin signaling regulate the neuronal cytoskeleton during migration? Neurogenesis 3:e1242455. doi: 10.1080/23262133.2016.1242455, PMID: 28265585 PMC5328581

[ref33] Chan J. R. Watkins T. A. Cosgaya J. M. Zhang C. Chen L. Reichardt L. F.. (2004). NGF controls axonal receptivity to myelination by Schwann cells or oligodendrocytes. Neuron 43, 183–191. doi: 10.1016/j.neuron.2004.06.024, PMID: 15260955 PMC2758239

[ref34] Chen G. K. Kono N. Geschwind D. H. Cantor R. M. (2006). Quantitative trait locus analysis of nonverbal communication in autism spectrum disorder. Mol. Psychiatry 11, 214–220. doi: 10.1038/sj.mp.4001753, PMID: 16189504

[ref35] Chiang C. Litingtung Y. Lee E. Young K. E. Corden J. L. Westphal H.. (1996). Cyclopia and defective axial patterning in mice lacking sonic hedgehog gene function. Nature 383, 407–413. doi: 10.1038/383407a0, PMID: 8837770

[ref36] Cho S. C. Kim H. W. Kim B. N. Kim J. W. Shin M. S. Cho D. Y.. (2010). Neurotrophin-3 gene, intelligence, and selective attention deficit in a Korean sample with attention-deficit/hyperactivity disorder. Prog. Neuro-Psychopharmacol. Biol. Psychiatry 34, 1065–1069. doi: 10.1016/j.pnpbp.2010.05.026 20576502

[ref37] Clouston T. S. (1891). The neuroses of development: being the Morison lectures for 1890. Edinb. Med. J. 36, 785–801.

[ref38] Cooper J. A. (2008). A mechanism for inside-out lamination in the neocortex. Trends Neurosci. 31, 113–119. doi: 10.1016/j.tins.2007.12.003, PMID: 18255163

[ref39] Cooper J. A. (2014). Molecules and mechanisms that regulate multipolar migration in the intermediate zone. Front. Cell. Neurosci. 8:386. doi: 10.3389/fncel.2014.00386, PMID: 25452716 PMC4231986

[ref40] Corroenne R. Arthuis C. Kasprian G. Mahallati H. Ville Y. Millischer Bellaiche A. E.. (2022). Diffusion tensor imaging of fetal brain: principles, potential and limitations of promising technique. Ultrasound Obstet. Gynecol. 60, 470–476. doi: 10.1002/uog.24935, PMID: 35561129

[ref41] Costa M. R. Kessaris N. Richardson W. D. Götz M. Hedin-Pereira C. (2007). The marginal zone/layer I as a novel niche for neurogenesis and gliogenesis in developing cerebral cortex. J. Neurosci. 27, 11376–11388. doi: 10.1523/JNEUROSCI.2418-07.2007, PMID: 17942732 PMC6673031

[ref42] Crama N. Toolens A. M. van der Meer J. W. Cruysberg J. R. (2004). Giant chalazia in the hyperimmunoglobulinemia E (hyper-IgE) syndrome. Eur. J. Ophthalmol. 14, 258–260. doi: 10.1177/112067210401400311, PMID: 15206652

[ref43] Cramer K. S. Miko I. J. (2016). Eph-ephrin signaling in nervous system development. F1000Res 5:F1000 Faculty Rev-413. doi: 10.12688/f1000research.7417.1, PMID: 27092247 PMC4821289

[ref44] Cui D. Zhang H. Yang B. Z. Listman J. B. Li D. Price L. H.. (2011). Variation in NGFB is associated with primary affective disorders in women. Am. J. Med. Genet. B 156B, 401–412. doi: 10.1002/ajmg.b.31175, PMID: 21294249 PMC3108453

[ref45] Curley A. A. Lewis D. A. (2012). Cortical basket cell dysfunction in schizophrenia. J. Physiol. 590, 715–724. doi: 10.1113/jphysiol.2011.224659, PMID: 22219337 PMC3381305

[ref46] Dawson E. Powell J. F. Sham P. C. Nöthen M. Crocq M.-A. Propping P.. (1995). An association study of a neurotrophin-3 (NT-3) gene polymorphism with schizophrenia. Acta Psychiatr. Scand. 92, 425–428. doi: 10.1111/j.1600-0447.1995.tb09607.x, PMID: 8837968

[ref47] Del Pino I. García-Frigola C. Dehorter N. Brotons-Mas J. R. Alvarez-Salvado E. Martínez de Lagrán M.. (2013). Erbb4 deletion from fast-spiking interneurons causes schizophrenia-like phenotypes. Neuron 79, 1152–1168. doi: 10.1016/j.neuron.2013.07.010, PMID: 24050403

[ref48] Delhaye-Bouchaud N. (2001). Development of the central nervous system in mammals. Neurophysiol. Clin. 31, 63–82. doi: 10.1016/s0987-7053(01)00249-0 11433675

[ref49] Dent E. W. Gupton S. L. Gertler F. B. (2011). The growth cone cytoskeleton in axon outgrowth and guidance. Cold Spring Harb. Perspect. Biol. 3:a001800. doi: 10.1101/cshperspect.a001800, PMID: 21106647 PMC3039926

[ref50] Detera-Wadleigh S. D. Berrettini W. H. Goldin L. R. Boorman D. Anderson S. Gershon E. S. (1987). Close linkage of c-Harvey-ras-1 and the insulin gene to affective disorder is ruled out in three North American pedigrees. Nature 325, 806–808. doi: 10.1038/325806a0, PMID: 3547139

[ref51] Dlugosz P. Nimpf J. (2018). The reelin receptors apolipoprotein E receptor 2 (ApoER2) and VLDL receptor. Int. J. Mol. Sci. 19:3090. doi: 10.3390/ijms19103090, PMID: 30304853 PMC6213145

[ref52] Dougnon G. Matsui H. (2022). Modelling autism spectrum disorder (ASD) and attention-deficit/hyperactivity disorder (ADHD) using mice and zebrafish. Int. J. Mol. Sci. 23:7550. doi: 10.3390/ijms23147550, PMID: 35886894 PMC9319972

[ref53] Draganov M. Arranz M. J. Salazar J. de Diego-Adeliño J. Gallego-Fabrega C. Jubero M.. (2019). Association study of polymorphisms within inflammatory genes and methylation status in treatment response in major depression. Eur. Psychiatry 60, 7–13. doi: 10.1016/j.eurpsy.2019.05.003, PMID: 31100612

[ref54] Egeland J. A. Gerhard D. S. Pauls D. L. Sussex J. N. Kidd K. K. Allen C. R.. (1987). Bipolar affective disorders linked to DNA markers on chromosome 11. Nature 325, 783–787. doi: 10.1038/325783a0, PMID: 2881209

[ref55] Elitt C. M. Malin S. A. Koerber H. R. Davis B. M. Albers K. M. (2008). Overexpression of artemin in the tongue increases expression of TRPV1 and TRPA1 in trigeminal afferents and causes oral sensitivity to capsaicin and mustard oil. Brain Res. 1230, 80–90. doi: 10.1016/j.brainres.2008.06.119, PMID: 18652806 PMC2570744

[ref56] Elitt C. M. McIlwrath S. L. Lawson J. J. Malin S. A. Molliver D. C. Cornuet P. K.. (2006). Artemin overexpression in skin enhances expression of TRPV1 and TRPA1 in cutaneous sensory neurons and leads to behavioral sensitivity to heat and cold. J. Neurosci. 26, 8578–8587. doi: 10.1523/JNEUROSCI.2185-06.2006, PMID: 16914684 PMC6674358

[ref57] Elorriaga V. Pierani A. Causeret F. (2023). Cajal–Retzius cells: recent advances in identity and function. Curr. Opin. Neurobiol. 79:102686. doi: 10.1016/j.conb.2023.102686, PMID: 36774666

[ref58] Encinas M. Iglesias M. Liu Y. Wang H. Muhaisen A. Ceña V.. (2000). Sequential treatment of SH-SY5Y cells with retinoic acid and brain-derived neurotrophic factor gives rise to fully differentiated, neurotrophic factor-dependent, human neuron-like cells. J. Neurochem. 75, 991–1003. doi: 10.1046/j.1471-4159.2000.0750991.x, PMID: 10936180

[ref59] Ernfors P. Ibáñez C. F. Ebendal T. Olson L. Persson H. (1990). Molecular cloning and neurotrophic activities of a protein with structural similarities to nerve growth factor: developmental and topographical expression in the brain. Proc. Natl. Acad. Sci. U.S.A. 87, 5454–5458. doi: 10.1073/pnas.87.14.5454, PMID: 2164684 PMC54343

[ref60] Fame R. M. Cortés-Campos C. Sive H. L. (2020). Brain ventricular system and cerebrospinal fluid development and function: light at the end of the tube: a primer with latest insights. BioEssays 42:e1900186. doi: 10.1002/bies.201900186, PMID: 32078177

[ref61] Fariñas I. Jones K. R. Backus C. Wang X. Y. Reichardt L. F. (1994). Severe sensory and sympathetic deficits in mice lacking neurotrophin-3. Nature 369, 658–661. doi: 10.1038/369658a0, PMID: 8208292

[ref62] Faust T. E. Gunner G. Schafer D. P. (2021). Mechanisms governing activity-dependent synaptic pruning in the developing mammalian CNS. Nat. Rev. Neurosci. 22, 657–673. doi: 10.1038/s41583-021-00507-y, PMID: 34545240 PMC8541743

[ref63] Favuzzi E. Deogracias R. Marques-Smith A. Maeso P. Jezequel J. Exposito-Alonso D.. (2019). Distinct molecular programs regulate synapse specificity in cortical inhibitory circuits. Science 363, 413–417. doi: 10.1126/science.aau8977, PMID: 30679375

[ref64] Fernàndez-Castillo N. Roncero C. Grau-Lopez L. Barral C. Prat G. Rodriguez-Cintas L.. (2013). Association study of 37 genes related to serotonin and dopamine neurotransmission and neurotrophic factors in cocaine dependence. Genes Brain Behav. 12, 39–46. doi: 10.1111/gbb.12013, PMID: 23241418

[ref65] Fiorentini C. Guerra N. Facchetti M. Finardi A. Tiberio L. Schiaffonati L.. (2002). Nerve growth factor regulates dopamine D(2) receptor expression in prolactinoma cell lines via p75(NGFR)-mediated activation of nuclear factor-kappaB. Mol. Endocrinol. 16, 353–366. doi: 10.1210/mend.16.2.0773 11818506

[ref66] Fleitas C. Marfull-Oromí P. Chauhan D. del Toro D. Peguera B. Zammou B.. (2021). FLRT2 and FLRT3 cooperate in maintaining the tangential migratory streams of cortical interneurons during development. J. Neurosci. 41, 7350–7362. doi: 10.1523/JNEUROSCI.0380-20.2021, PMID: 34301831 PMC8412983

[ref67] Freedman R. Strömberg I. Nordström A. L. Seiger A. Olson L. Bygdeman M.. (1994). Neuronal development in embryonic brain tissue derived from schizophrenic women and grafted to animal hosts. Schizophr. Res. 13, 259–270. doi: 10.1016/0920-9964(94)90051-5, PMID: 7841140

[ref68] Freedman R. Strömberg I. Seiger A. Olson L. Nordström A. L. Wiesel F. A.. (1992). Initial studies of embryonic transplants of human hippocampus and cerebral cortex derived from schizophrenic women. Biol. Psychiatry 32, 1148–1163. doi: 10.1016/0006-3223(92)90194-5, PMID: 1362085

[ref69] Friedman W. J. Ibáñez C. F. Hallböök F. Persson H. Cain L. D. Dreyfus C. F.. (1993). Differential actions of neurotrophins in the locus coeruleus and basal forebrain. Exp. Neurol. 119, 72–78. doi: 10.1006/exnr.1993.1007, PMID: 8432352

[ref70] Friedman W. J. Ibáñez C. F. Hallböök F. Persson H. Cain L. D. Dreyfus C. F.. (2002). Physiological and morphological plasticity induced by chronic treatment with NT-3 or NT-4/5 in hippocampal slice cultures. Eur. J. Neurosci. 16, 1939–1948. doi: 10.1046/j.1460-9568.2002.02259.x, PMID: 12453058

[ref71] Gall C. M. Gold S. J. Isackson P. J. Seroogy K. B. (1992). Brain-derived neurotrophic factor and neurotrophin-3 mRNAs are expressed in ventral midbrain regions containing dopaminergic neurons. Mol. Cell. Neurosci. 3, 56–63. doi: 10.1016/1044-7431(92)90009-Q, PMID: 19912846

[ref72] Gardner E. J. Stephens F. E. (1949). Schizophrenia in monozygotic twins. J. Hered. 40, 165–167. doi: 10.1093/oxfordjournals.jhered.a106022, PMID: 18145584

[ref73] Gareeva A. E. Traks T. Koks S. Khusnutdinova E. K. (2015). The role of neurotrophins and neurexins genes in the risk of paranoid schizophrenia in Russians and Tatars. Genetika 51, 799–811. doi: 10.7868/S0016675815060065 PMID: 26410934

[ref74] Gasiorowska A. Wydrych M. Drapich P. Zadrozny M. Steczkowska M. Niewiadomski W.. (2021). The biology and pathobiology of glutamatergic, cholinergic, and dopaminergic signaling in the aging brain. Front. Aging Neurosci. 13:654931. doi: 10.3389/fnagi.2021.654931, PMID: 34326765 PMC8315271

[ref75] Gassó P. Ortiz A. E. Mas S. Morer A. Calvo A. Bargalló N.. (2015). Association between genetic variants related to glutamatergic, dopaminergic and neurodevelopment pathways and white matter microstructure in child and adolescent patients with obsessive-compulsive disorder. J. Affect. Disord. 186, 284–292. doi: 10.1016/j.jad.2015.07.035, PMID: 26254621

[ref76] Gibbs R. B. (1994). Estrogen and nerve growth factor-related systems in brain. Effects on basal forebrain cholinergic neurons and implications for learning and memory processes and aging. Ann. N. Y. Acad. Sci. 743, 165–199. doi: 10.1111/j.1749-6632.1994.tb55792.x 7802412

[ref77] Gilabert-Juan J. Sáez A. R. Lopez-Campos G. Sebastiá-Ortega N. González-Martínez R. Costa J.. (2015). Semaphorin and plexin gene expression is altered in the prefrontal cortex of schizophrenia patients with and without auditory hallucinations. Psychiatry Res. 229, 850–857. doi: 10.1016/j.psychres.2015.07.074, PMID: 26243375

[ref78] Gilmore E. C. Ohshima T. Goffinet A. M. Kulkarni A. B. Herrup K. (1998). Cyclin-dependent kinase 5-deficient mice demonstrate novel developmental arrest in cerebral cortex. J. Neurosci. 18, 6370–6377. doi: 10.1523/JNEUROSCI.18-16-06370.1998, PMID: 9698328 PMC6793186

[ref79] Glessner J. T. Reilly M. P. Kim C. E. Takahashi N. Albano A. Hou C.. (2010). Strong synaptic transmission impact by copy number variations in schizophrenia. Proc. Natl. Acad. Sci. U.S.A. 107, 10584–10589. doi: 10.1073/pnas.1000274107, PMID: 20489179 PMC2890845

[ref80] Goedert M. Fine A. Hunt S. P. Ullrich A. (1986). Nerve growth factor mRNA in peripheral and central rat tissues and in the human central nervous system: lesion effects in the rat brain and levels in Alzheimer’s disease. Brain Res. 387, 85–92. doi: 10.1016/0169-328x(86)90023-9, PMID: 3742235

[ref81] Gonda Y. Namba T. Hanashima C. (2020). Beyond axon guidance: roles of slit-robo signaling in neocortical formation. Front. Cell Dev. Biol. 8:607415. doi: 10.3389/fcell.2020.607415, PMID: 33425915 PMC7785817

[ref82] Google Patents (2007). Method and kit for diagnosing autism using gene expression profiling. Available at: https://patents.google.com/patent/US8962307B2/en (Accessed October 25, 2023).

[ref83] Gören J. L. (2016). Brain-derived neurotrophic factor and schizophrenia. Ment. Health Clin. 6, 285–288. doi: 10.9740/mhc.2016.11.285, PMID: 29955483 PMC6007539

[ref84] Goto Y. Grace A. A. (2008). Limbic and cortical information processing in the nucleus accumbens. Trends Neurosci. 31, 552–558. doi: 10.1016/j.tins.2008.08.002, PMID: 18786735 PMC2884964

[ref85] Grafton S. T. Mazziotta J. C. Presty S. Friston K. J. Frackowiak R. S. Phelps M. E. (1992). Functional anatomy of human procedural learning determined with regional cerebral blood flow and PET. J. Neurosci. 12, 2542–2548. doi: 10.1523/JNEUROSCI.12-07-02542.1992, PMID: 1613546 PMC6575851

[ref86] Greene N. D. Copp A. J. (2009). Development of the vertebrate central nervous system: formation of the neural tube. Prenat. Diagn. 29, 303–311. doi: 10.1002/pd.2206, PMID: 19206138

[ref87] Gurrapu S. Franzolin G. Fard D. Accardo M. Medico E. Sarotto I.. (2019). Reverse signaling by semaphorin 4C elicits SMAD1/5-and ID1/3-dependent invasive reprogramming in cancer cells. Sci. Signal. 12:eaav2041. doi: 10.1126/scisignal.aav2041 31431542

[ref88] Halperin D. Stavsky A. Kadir R. Drabkin M. Wormser O. Yogev Y.. (2021). CDH2 mutation affecting N-cadherin function causes attention-deficit hyperactivity disorder in humans and mice. Nat. Commun. 12:6187. doi: 10.1038/s41467-021-26426-1 34702855 PMC8548587

[ref89] Hanaoka Y. Ohi T. Furukawa S. Furukawa Y. Hayashi K. Matsukura S. (1994). The therapeutic effects of 4-methylcatechol, a stimulator of endogenous nerve growth factor synthesis, on experimental diabetic neuropathy in rats. J. Neurosci. 122, 28–32. doi: 10.1016/0022-510x(94)90048-5 8195800

[ref90] Hashimoto T. Bergen S. E. Nguyen Q. L. Xu B. Monteggia L. M. Pierri J. N.. (2005). Relationship of brain-derived neurotrophic factor and its receptor TrkB to altered inhibitory prefrontal circuitry in schizophrenia. J. Neurosci. 25, 372–383. doi: 10.1523/JNEUROSCI.4035-04.2005, PMID: 15647480 PMC6725470

[ref91] Hattori M. Nanko S. (1995). Association of neurotrophin-3 gene variant with severe forms of schizophrenia. Biochem. Biophys. Res. Commun. 209, 513–518. doi: 10.1006/bbrc.1995.1531, PMID: 7733919

[ref92] He C. Y. Wang Z. T. Shen Y. Y. Shi A. Y. Li H. Y. Chen D. W.. (2022). Association of rs2072446 in the NGFR gene with the risk of Alzheimer's disease and amyloid-β deposition in the brain. CNS Neurosci. Ther. 28, 2218–2229. doi: 10.1111/cns.13965, PMID: 36074475 PMC9627368

[ref93] Hefti F. Hartikka J. Salvatierra A. Weiner W. J. Mash D. C. (1986). Localization of nerve growth factor receptors in cholinergic neurons of the human basal forebrain. Neurosci. Lett. 69, 37–41. doi: 10.1016/0304-3940(86)90410-6, PMID: 3018635

[ref94] Holtzman D. M. Mobley W. C. (1994). Neurotrophic factors and neurologic disease. West. J. Med. 161, 246–254. doi: 10.3390/ijms24043866 7975562 PMC1011405

[ref95] Honma Y. Araki T. Gianino S. Bruce A. Heuckeroth R. Johnson E.. (2002). Artemin is a vascular-derived neurotropic factor for developing sympathetic neurons. Neuron 35, 267–282. doi: 10.1016/S0896-6273(02)00774-2, PMID: 12160745

[ref96] Hsu W. F. Chien W. C. Chung C. H. Lee P. C. Wang D. S. Huang S. W.. (2021). Association between tetralogy of Fallot and psychiatric disorders: a nationwide cohort study. J. Clin. Psychiatry 82:19m13126. doi: 10.4088/JCP.19m13126 33988933

[ref97] Huang E. J. Reichardt L. F. (2001). Neurotrophins: roles in neuronal development and function. Annu. Rev. Neurosci. 24, 677–736. doi: 10.1146/annurev.neuro.24.1.677, PMID: 11520916 PMC2758233

[ref98] Hughes A. N. (2021). Glial cells promote myelin formation and elimination. Front. Cell Dev. Biol. 9:661486. doi: 10.3389/fcell.2021.661486, PMID: 34046407 PMC8144722

[ref99] Hwang J. P. Tsai S. J. Hong C. J. Yang C. H. Lirng J. F. Yang Y. M.. (2006). The Val66Met polymorphism of the brain-derived neurotrophic-factor gene is associated with geriatric depression. Neurobiol. Aging 27, 1834–1837. doi: 10.1016/j.neurobiolaging.2005.10.013, PMID: 16343697

[ref100] Ikeda M. Tomita Y. Mouri A. Koga M. Okochi T. Yoshimura R.. (2010). Identification of novel candidate genes for treatment response to risperidone and susceptibility for schizophrenia: integrated analysis among pharmacogenomics, mouse expression, and genetic case-control association approaches. Biol. Psychiatry 67, 263–269. doi: 10.1016/j.biopsych.2009.08.030, PMID: 19850283

[ref101] Ip N. Y. Ibáñez C. F. Nye S. H. McClain J. Jones P. F. Gies D. R.. (1992). Mammalian neurotrophin-4: structure, chromosomal localization, tissue distribution, and receptor specificity. Proc. Natl. Acad. Sci. U.S.A. 89, 3060–3064. doi: 10.1073/pnas.89.7.3060, PMID: 1313578 PMC48803

[ref102] Ishii K. Kubo K. I. Nakajima K. (2016). Reelin and neuropsychiatric disorders. Front. Cell. Neurosci. 10:229. doi: 10.3389/fncel.2016.00229, PMID: 27803648 PMC5067484

[ref103] Ishikawa M. Omachi Y. Sato N. Nakagawa E. (2020). Bipolar disorder in megalencephalic leukoencephalopathy with subcortical cysts: a case report. BMC Psychiatry 20:349. doi: 10.1186/s12888-020-02750-6 32620087 PMC7333431

[ref104] Issa G. Wilson C. Terry A. V. Jr. Pillai A. (2010). An inverse relationship between cortisol and BDNF levels in schizophrenia: data from human postmortem and animal studies. Neurobiol. Dis. 39, 327–333. doi: 10.1016/j.nbd.2010.04.017, PMID: 20451611

[ref105] Jiang X. Shen S. Cadwell C. R. Berens P. Sinz F. Ecker A. S.. (2015). Principles of connectivity among morphologically defined cell types in adult neocortex. Science 350:aac9462. doi: 10.1126/science.aac9462 26612957 PMC4809866

[ref106] Jiao B. Liu S. Tan X. Lu P. Wang D. Xu H. (2021). Class-3 semaphorins: potent multifunctional modulators for angiogenesis-associated diseases. Biomed. Pharmacother. 137:111329. doi: 10.1016/j.biopha.2021.111329, PMID: 33545660

[ref107] Johnson S. B. Blum R. W. Giedd J. N. (2009). Adolescent maturity and the brain: the promise and pitfalls of neuroscience research in adolescent health policy. J. Adolesc. Health 45, 216–221. doi: 10.1016/j.jadohealth.2009.05.016, PMID: 19699416 PMC2892678

[ref108] Jŏnsson E. Brené S. Zhang X. R. Nimgaonkar V. L. Tylec A. Schalling M.. (1997). Schizophrenia and neurotrophin-3 alleles. Acta Psychiatr. Scand. 95, 414–419. doi: 10.1111/j.1600-0447.1997.tb09654.x, PMID: 9197906

[ref109] Jovanovic J. N. Thomson A. M. (2011). Development of cortical GABAergic innervation. Front. Cell. Neurosci. 5:14. doi: 10.3389/fncel.2011.00014, PMID: 21808605 PMC3139172

[ref110] Kallmann F. J. (1946). The genetic theory of schizophrenia; an analysis of 691 schizophrenic twin index families. Am. J. Psychiatry 103, 309–322. doi: 10.1176/ajp.103.3.309, PMID: 20277893

[ref111] Kang H., and, Schumann E. M. (1996). A requirement for local protein synthesis in neurotrophin-induced hippocampal synaptic plasticity. Science 273, 1402–1406. doi: 10.1126/science.273.5280.1402, PMID: 8703078

[ref112] Kapelski P. Skibinska M. Maciukiewicz M. Wilkosc M. Frydecka D. Groszewska A.. (2015). Association study of functional polymorphisms in interleukins and interleukin receptors genes: IL1A, IL1B, IL1RN, IL6, IL6R, IL10, IL10RA and TGFB1 in schizophrenia in polish population. Schizophr. Res. 169, 1–9. doi: 10.1016/j.schres.2015.10.008, PMID: 26481614

[ref113] Karagyaur M. Primak A. Efimenko A. Skryabina M. Tkachuk V. (2022). The power of gene technologies: 1001 ways to create a cell model. Cells 11:3235. doi: 10.3390/cells11203235, PMID: 36291103 PMC9599997

[ref114] Kazanis I. Lathia J. Moss L. Ffrench-Constant C. (2008). “The neural stem cell microenvironment” in StemBook (Cambridge, MA: Harvard Stem Cell Institute) Available at: https://www.stembook.org/node/490 20614585

[ref115] Kéri S. Kiss I. Seres I. Kelemen O. A. (2009). Polymorphism of the neuregulin 1 gene (SNP8NRG243177/rs6994992) affects reactivity to expressed emotion in schizophrenia. Am. J. Med. Genet. B 150B, 418–420. doi: 10.1002/ajmg.b.30812, PMID: 18543275

[ref116] Khandaker G. M. Zammit S. Burgess S. Lewis G. Jones P. B. (2018). Association between a functional interleukin 6 receptor genetic variant and risk of depression and psychosis in a population-based birth cohort. Brain Behav. Immun. 69, 264–272. doi: 10.1016/j.bbi.2017.11.020, PMID: 29197507 PMC5871397

[ref117] Klämbt C. (2009). Modes and regulation of glial migration in vertebrates and invertebrates. Nat. Rev. Neurosci. 10, 769–779. doi: 10.1038/nrn2720, PMID: 19773781

[ref118] Kong A. Steinthorsdottir V. Masson G. Thorleifsson G. Sulem P. Besenbacher S.. (2009). Parental origin of sequence variants associated with complex diseases. Nature 462, 868–874. doi: 10.1038/nature08625, PMID: 20016592 PMC3746295

[ref119] Koropouli E. Kolodkin A. L. (2014). Semaphorins and the dynamic regulation of synapse assembly, refinement, and function. Curr. Opin. Neurobiol. 27, 1–7. doi: 10.1016/j.conb.2014.02.005, PMID: 24598309 PMC4122587

[ref120] Kostović I. Jovanov-Milosević N. (2006). The development of cerebral connections during the first 20–45 weeks’ gestation. Semin. Fetal Neonatal Med. 11, 415–422. doi: 10.1016/j.siny.2006.07.001, PMID: 16962836

[ref121] Kotyuk E. Keszler G. Nemeth N. Ronai Z. Sasvari-Szekely M. Szekely A. (2013). Glial cell line-derived neurotrophic factor (GDNF) as a novel candidate gene of anxiety. PLoS One 8:e80613. doi: 10.1371/journal.pone.0080613, PMID: 24324616 PMC3855631

[ref122] Kovacs D. Eszlari N. Petschner P. Pap D. Vas S. Kovacs P.. (2016). Interleukin-6 promoter polymorphism interacts with pain and life stress influencing depression phenotypes. J. Neural Transm. 123, 541–548. doi: 10.1007/s00702-016-1506-9, PMID: 26821321 PMC4846685

[ref123] Kranz T. M. Goetz R. R. Walsh-Messinger J. Goetz D. Antonius D. Dolgalev I.. (2015). Rare variants in the neurotrophin signaling pathway implicated in schizophrenia risk. Schizophr. Res. 168, 421–428. doi: 10.1016/j.schres.2015.07.002, PMID: 26215504 PMC4591185

[ref124] Kummer K. K. Zeidler M. Kalpachidou T. Kress M. (2021). Role of IL-6 in the regulation of neuronal development, survival and function. Cytokine 144:155582. doi: 10.1016/j.cyto.2021.155582, PMID: 34058569

[ref125] Kunugi H. Ueki A. Otsuka M. Isse K. Hirasawa H. Kato N.. (2001). A novel polymorphism of the brain-derived neurotrophic factor (BDNF) gene associated with late-onset Alzheimer’s disease. Mol. Psychiatry 6, 83–86. doi: 10.1038/sj.mp.4000792, PMID: 11244490

[ref126] Kushima I. Nakamura Y. Aleksic B. Ikeda M. Ito Y. Shiino T.. (2012). Resequencing and association analysis of the KALRN and EPHB1 genes and their contribution to schizophrenia susceptibility. Schizophr. Bull. 38, 552–560. doi: 10.1093/schbul/sbq118, PMID: 21041834 PMC3329972

[ref127] Kwon Y. T. Gupta A. Zhou Y. Nikolic M. Tsai L. H. (2000). Regulation of N-cadherin-mediated adhesion by the p35-Cdk5 kinase. Curr. Biol. 10, 363–372. doi: 10.1016/S0960-9822(00)00411-5, PMID: 10753743

[ref128] Large T. H. Bodary S. C. Clegg D. O. Weskamp G. Otten U. Reichardt L. F. (1986). Nerve growth factor gene expression in the developing rat brain. Science 234, 352–355. doi: 10.1126/science.3764415, PMID: 3764415

[ref129] Lasky-Su J. Neale B. M. Franke B. Anney R. J. Zhou K. Maller J. B.. (2008). Genome-wide association scan of quantitative traits for attention deficit hyperactivity disorder identifies novel associations and confirms candidate gene associations. Am. J. Med. Genet. B 147B, 1345–1354. doi: 10.1002/ajmg.b.30867, PMID: 18821565

[ref130] Lathia J. D. Rao M. S. Mattson M. P. Ffrench-Constant C. (2007). The microenvironment of the embryonic neural stem cell: lessons from adult niches? Dev. Dyn. 236, 3267–3282. doi: 10.1002/dvdy.21319, PMID: 17937403

[ref131] Lavedan C. Volpi S. Polymeropoulos M. H. Wolfgang C. D. (2008). Effect of a ciliary neurotrophic factor polymorphism on schizophrenia symptom improvement in an iloperidone clinical trial. Pharmacogenomics 9, 289–301. doi: 10.2217/14622416.9.3.289, PMID: 18303965

[ref132] Law J. W. Lee A. Y. (2012). The role of semaphorins and their receptors in gliomas. J. Signal. 2012:902854. doi: 10.1155/2012/902854, PMID: 23050142 PMC3461631

[ref133] Lee K. Kunugi H. Nanko S. (2001). Glial cell line-derived neurotrophic factor (GDNF) gene and schizophrenia: polymorphism screening and association analysis. Psychiatry Res. 104, 11–17. doi: 10.1016/S0165-1781(01)00294-3, PMID: 11600185

[ref134] Léger C. Dupré N. Aligny C. Bénard M. Lebon A. Henry V.. (2020). Glutamate controls vessel-associated migration of GABA interneurons from the pial migratory route via NMDA receptors and endothelial protease activation. Cell. Mol. Life Sci. 77, 1959–1986. doi: 10.1007/s00018-019-03248-5, PMID: 31392351 PMC7229000

[ref135] Levi-Montalcini R. (1964). The nerve growth factor. Ann. N. Y. Acad. Sci. 18, 149–170. doi: 10.1111/j.1749-6632.1964.tb33978.x 14258355

[ref136] Levi-Montalcini R. (1987). The nerve growth factor: thirty-five years later. Biosci. Rep. 7, 681–699. doi: 10.1007/BF01116861, PMID: 3322422

[ref137] Lewis D. A. Curley A. A. Glausier J. R. Volk D. W. (2012). Cortical parvalbumin interneurons and cognitive dysfunction in schizophrenia. Trends Neurosci. 35, 57–67. doi: 10.1016/j.tins.2011.10.004, PMID: 22154068 PMC3253230

[ref138] Li H. Lu Q. Xiao E. Li Q. He Z. Mei X. (2014). Methamphetamine enhances the development of schizophrenia in first-degree relatives of patients with schizophrenia. Can. J. Psychiatr. 59, 107–113. doi: 10.1177/070674371405900206, PMID: 24881129 PMC4079234

[ref140] Liao H. M. Chao Y. L. Huang A. L. Cheng M. C. Chen Y. J. Lee K. F.. (2012). Identification and characterization of three inherited genomic copy number variations associated with familial schizophrenia. Schizophr. Res. 139, 229–236. doi: 10.1016/j.schres.2012.05.015, PMID: 22682706

[ref141] Licinio J. Dong C. Wong M. L. (2009). Novel sequence variations in the brain-derived neurotrophic factor gene and association with major depression and antidepressant treatment response. Arch. Gen. Psychiatry 66, 488–497. doi: 10.1001/archgenpsychiatry.2009.38, PMID: 19414708 PMC4272010

[ref142] Lin L. F. Doherty D. H. Lile J. D. Bektesh S. Collins F. (1993). GDNF: a glial cell line-derived neurotrophic factor for midbrain dopaminergic neurons. Science 260, 1130–1132. doi: 10.1126/science.8493557, PMID: 8493557

[ref143] Lin Z. Su Y. Zhang C. Xing M. Ding W. Liao L.. (2013). The interaction of BDNF and NTRK2 gene increases the susceptibility of paranoid schizophrenia. PLoS One 8:e74264. doi: 10.1371/journal.pone.0074264, PMID: 24069289 PMC3775790

[ref144] Llorca A. Deogracias R. (2022). Origin, development, and synaptogenesis of cortical interneurons. Front. Neurosci. 16:929469. doi: 10.3389/fnins.2022.929469, PMID: 35833090 PMC9272671

[ref145] Loch A. A. Zanetti M. V. de Sousa R. T. Chaim T. M. Serpa M. H. Gattaz W. F.. (2015). Elevated neurotrophin-3 and neurotrophin 4/5 levels in unmedicated bipolar depression and the effects of lithium. Prog. Neuro-Psychopharmacol. Biol. Psychiatry 56, 243–246. doi: 10.1016/j.pnpbp.2014.09.014 25290636

[ref146] Lu C. L. Wang Y. C. Chen J. Y. Lai I. C. Liou Y. J. (2010). Support for the involvement of the ERBB4 gene in schizophrenia: a genetic association analysis. Neurosci. Lett. 481, 120–125. doi: 10.1016/j.neulet.2010.06.067, PMID: 20600594

[ref147] Lu D. Wang M. Yang T. Wang J. Lin B. Liu G.. (2023). Association of Interleukin-6 polymorphisms with schizophrenia and depression: a case-control study. Lab. Med. 54, 250–255. doi: 10.1093/labmed/lmac099, PMID: 36239635

[ref148] Lu A. T. Yoon J. Geschwind D. H. Cantor R. M. (2013). QTL replication and targeted association highlight the nerve growth factor gene for nonverbal communication deficits in autism spectrum disorders. Mol. Psychiatry 18, 226–235. doi: 10.1038/mp.2011.155, PMID: 22105621 PMC3586745

[ref149] Luhmann H. J. Fukuda A. Kilb W. (2015). Control of cortical neuronal migration by glutamate and GABA. Front. Cell. Neurosci. 9:4. doi: 10.3389/fncel.2015.00004, PMID: 25688185 PMC4311642

[ref150] Lukito S. Norman L. Carlisi C. Radua J. Hart H. Simonoff E.. (2020). Comparative meta-analyses of brain structural and functional abnormalities during cognitive control in attention-deficit/hyperactivity disorder and autism spectrum disorder. Psychol. Med. 50, 894–919. doi: 10.1017/S0033291720000574, PMID: 32216846 PMC7212063

[ref151] Ma X. C. Chen C. Zhu F. Jia W. Gao C. G. (2013). Association of the GDNF gene with depression and heroin dependence, but not schizophrenia, in a Chinese population. Psychiatry Res. 210, 1296–1298. doi: 10.1016/j.psychres.2013.08.025, PMID: 24022000

[ref152] Maisonpierre P. C. Belluscio L. Friedman B. Alderson R. F. Wiegand S. J. Furth M. E.. (1990a). NT-3, BDNF, and NGF in the developing rat nervous system: parallel as well as reciprocal patterns of expression. Neuron 5, 501–509. doi: 10.1016/0896-6273(90)90089-x, PMID: 1688327

[ref153] Maisonpierre P. C. Belluscio L. Squinto S. Ip N. Y. Furth M. E. Lindsay R. M.. (1990b). Neurotrophin-3: a neurotrophic factor related to NGF and BDNF. Science 247, 1446–1451. doi: 10.1126/science.247.4949.1446 2321006

[ref154] Marín O. (2013). Cellular and molecular mechanisms controlling the migration of neocortical interneurons. Eur. J. Neurosci. 38, 2019–2029. doi: 10.1111/ejn.12225, PMID: 23651101

[ref155] Materna-Kiryluk A. Pollak A. Gawalski K. Szczawinska-Poplonyk A. Rydzynska Z. Sosnowska A.. (2021). Mosaic IL6ST variant inducing constitutive GP130 cytokine receptor signaling as a cause of neonatal onset immunodeficiency with autoinflammation and dysmorphy. Hum. Mol. Genet. 30, 226–233. doi: 10.1093/hmg/ddab035 33517393

[ref156] Mercader J. M. Saus E. Agüera Z. Bayés M. Boni C. Carreras A.. (2008). Association of NTRK3 and its interaction with NGF suggest an altered cross-regulation of the neurotrophin signaling pathway in eating disorders. Hum. Mol. Genet. 17, 1234–1244. doi: 10.1093/hmg/ddn013, PMID: 18203754

[ref157] Meyerink B. L. Tiwari N. K. Pilaz L. J. (2020). Ariadne’s thread in the developing cerebral cortex: mechanisms enabling the guiding role of the radial glia basal process during neuron migration. Cells 10:3. doi: 10.3390/cells10010003 33375033 PMC7822038

[ref158] Michelato A. Bonvicini C. Ventriglia M. Scassellati C. Randazzo R. Bignotti S.. (2004). 3′ UTR (AGG)_n_ repeat of glial cell line-derived neurotrophic factor (GDNF) gene polymorphism in schizophrenia. Neurosci. Lett. 357, 235–237. doi: 10.1016/j.neulet.2003.12.089, PMID: 15003293

[ref159] Miguel P. M. Pereira L. O. Silveira P. P. Meaney M. J. (2019). Early environmental influences on the development of children’s brain structure and function. Dev. Med. Child Neurol. 61, 1127–1133. doi: 10.1111/dmcn.14182, PMID: 30740660

[ref160] Mitre M. Mariga A. Chao M. V. (2017). Neurotrophin signalling: novel insights into mechanisms and pathophysiology. Clin. Sci. 131, 13–23. doi: 10.1042/CS20160044, PMID: 27908981 PMC5295469

[ref161] Mobley W. C. Rutkowsky J. L. Tennekoon G. I. Gemsky J. Buchanan K. Johnston M. V. (1986). Nerve growth factor increases choline acetyltransferase activity in developing basal forebrain neurons. Mol. Brain Res. 1, 53–62. doi: 10.1016/0169-328X(86)90020-3, PMID: 3742234

[ref162] Moises H. W. Zoega T. Gottesman I. I. (2002). The glial growth factors deficiency and synaptic destabilization hypothesis of schizophrenia. BMC Psychiatry 2:8. doi: 10.1186/1471-244x-2-8 12095426 PMC117774

[ref163] Moradkhani A. Turki Jalil A. Mahmood Saleh M. Vanaki E. Daghagh H. Daghighazar B.. (2023). Correlation of rs35753505 polymorphism in neuregulin 1 gene with psychopathology and intelligence of people with schizophrenia. Gene 867:147285. doi: 10.1016/j.gene.2023.147285, PMID: 36905948

[ref164] Morrow B. A. Roth R. H. Redmond D. E. Elsworth J. D. (2011). Impact of methamphetamine on dopamine neurons in primates is dependent on age: implications for development of Parkinson’s disease. Neuroscience 189, 277–285. doi: 10.1016/j.neuroscience.2011.05.046, PMID: 21640165 PMC3150352

[ref165] Mulherkar S. Tolias K. F. (2020). RhoA-ROCK signaling as a therapeutic target in traumatic brain injury. Cells 9:245. doi: 10.3390/cells9010245, PMID: 31963704 PMC7016605

[ref166] Naka A. Veit J. Shababo B. Chance R. K. Risso D. Stafford D.. (2019). Complementary networks of cortical somatostatin interneurons enforce layer specific control. eLife 8:e43696. doi: 10.7554/eLife.43696, PMID: 30883329 PMC6422636

[ref167] Nanko S. Hattori M. Kuwata S. Sasaki T. Fukuda R. Dai X. Y.. (1994). Neurotrophin-3 gene polymorphism associated with schizophrenia. Acta Psychiatr. Scand. 89, 390–392. doi: 10.1111/j.1600-0447.1994.tb01534.x, PMID: 8085468

[ref168] Nanni L. Ming J. E. Bocian M. Steinhaus K. Bianchi D. W. Die-Smulders C.. (1999). The mutational spectrum of the sonic hedgehog gene in holoprosencephaly: SHH mutations cause a significant proportion of autosomal dominant holoprosencephaly. Hum. Mol. Genet. 8, 2479–2488. doi: 10.1093/hmg/8.13.2479, PMID: 10556296

[ref169] National Cancer Institute (2024). NCI dictionary of cancer terms (microenvironment). Available at: https://www.cancer.gov/publications/dictionaries/cancer-terms/def/microenvironment (Accessed February 18, 2024).

[ref170] Nibuya M. Morinobu S. Duman R. S. (1995). Regulation of BDNF and trkB mRNA in rat brain by chronic electroconvulsive seizure and antidepressant drug treatments. J. Neurosci. 15, 7539–7547. doi: 10.1523/JNEUROSCI.15-11-07539.1995, PMID: 7472505 PMC6578063

[ref171] Nicodemus K. K. Luna A. Vakkalanka R. Goldberg T. Egan M. Straub R. E.. (2006). Further evidence for association between ErbB4 and schizophrenia and influence on cognitive intermediate phenotypes in healthy controls. Mol. Psychiatry 11, 1062–1065. doi: 10.1038/sj.mp.4001878, PMID: 17130882

[ref172] Nicolas G. Sanchez-Contreras M. Ramos E. M. Lemos R. R. Ferreira J. Moura D.. (2017). Brain calcifications and PCDH12 variants. Neurol. Genet. 3:e166. doi: 10.1212/NXG.0000000000000166, PMID: 28804758 PMC5530423

[ref173] Niederkofler V. Baeriswyl T. Ott R. Stoeckli E. T. (2010). Nectin-like molecules/SynCAMs are required for post-crossing commissural axon guidance. Development 137, 427–435. doi: 10.1242/dev.042515, PMID: 20056680

[ref174] Nimgaonkar V. L. Zhang X. R. Brar J. S. DeLeo M. Ganguli R. (1995). Lack of association of schizophrenia with the neurotrophin-3 gene locus. Acta Psychiatr. Scand. 92, 464–466. doi: 10.1111/j.1600-0447.1995.tb09614.x, PMID: 8837975

[ref175] Okahisa Y. Ujike H. Kunugi H. Ishihara T. Kodama M. Takaki M.. (2010). Leukemia inhibitory factor gene is associated with schizophrenia and working memory function. Prog. Neuro-Psychopharmacol. Biol. Psychiatry 34, 172–176. doi: 10.1016/j.pnpbp.2009.10.020 19879916

[ref176] Oppenheim J. S. Skerry J. E. Tramo M. J. Gazzaniga M. S. (1989). Magnetic resonance imaging morphology of the corpus callosum in monozygotic twins. Ann. Neurol. 26, 100–104. doi: 10.1002/ana.410260117, PMID: 2774498

[ref177] Pardiñas A. F. Holmans P. Pocklington A. J. Escott-Price V. Ripke S. Carrera N.. (2018). Common schizophrenia alleles are enriched in mutation-intolerant genes and in regions under strong background selection. Nat. Genet. 50, 381–389. doi: 10.1038/s41588-018-0059-2, PMID: 29483656 PMC5918692

[ref178] Park J. K. Lee S. M. Kang W. S. Kim S. K. Cho A. R. (2011). NGF polymorphisms and haplotypes are associated with schizophrenia in Korean population. Mol. Cell Toxicol. 7, 375–380. doi: 10.1007/s13273-011-0047-4

[ref179] Pasquin S. Sharma M. Gauchat J. F. (2015). Ciliary neurotrophic factor (CNTF): new facets of an old molecule for treating neurodegenerative and metabolic syndrome pathologies. Cytokine Growth Factor Rev. 26, 507–515. doi: 10.1016/j.cytogfr.2015.07.007, PMID: 26187860

[ref180] Paxinos G. Marín O. (2015). “Chapter 3—tangential migration in the telencephalon” in The rat nervous system. 4th ed (USA: Academic Press), 45–58.

[ref181] Penisson M. Ladewig J. Belvindrah R. Francis F. (2019). Genes and mechanisms involved in the generation and amplification of basal radial glial cells. Front. Cell. Neurosci. 13:381. doi: 10.3389/fncel.2019.00381 31481878 PMC6710321

[ref182] Pérez Y. Bonet R. Corredor M. Domingo C. Moure A. Messeguer À.. (2021). Semaphorin 3A-glycosaminoglycans interaction as therapeutic target for axonal regeneration. Pharmaceuticals 14:906. doi: 10.3390/ph14090906, PMID: 34577606 PMC8465649

[ref183] Perez-Branguli F. Zagar Y. Shanley D. K. Graef I. A. Chédotal A. Mitchell K. J. (2016). Reverse signaling by semaphorin-6A regulates cellular aggregation and neuronal morphology. PLoS One 11:e0158686. doi: 10.1371/journal.pone.0158686, PMID: 27392094 PMC4938514

[ref184] Pfeffer C. K. Xue M. He M. Huang Z. J. Scanziani M. (2013). Inhibition of inhibition in visual cortex: the logic of connections between molecularly distinct interneurons. Nat. Neurosci. 16, 1068–1076. doi: 10.1038/nn.3446, PMID: 23817549 PMC3729586

[ref185] Ping J. Zhang J. Wan J. Huang C. Luo J. Du B.. (2022). Polymorphism in the BDNF gene (rs11030101) is associated with negative symptoms in Chinese Han patients with schizophrenia. Front. Genet. 13:849227. doi: 10.3389/fgene.2022.849227, PMID: 35368680 PMC8974295

[ref186] Powell E. M. Campbell D. B. Stanwood G. D. Davis C. Noebels J. L. Levitt P. (2003). Genetic disruption of cortical interneuron development causes region-and GABA cell type-specific deficits, epilepsy, and behavioral dysfunction. J. Neurosci. 23, 622–631. doi: 10.1523/JNEUROSCI.23-02-00622.2003, PMID: 12533622 PMC6741866

[ref187] Pöyhönen S. Er S. Domanskyi A. Airavaara M. (2019). Effects of neurotrophic factors in glial cells in the central nervous system: expression and properties in neurodegeneration and injury. Front. Physiol. 10:486. doi: 10.3389/fphys.2019.00486 31105589 PMC6499070

[ref188] Prakash N. Wurst W. (2006). Development of dopaminergic neurons in the mammalian brain. Cell. Mol. Life Sci. 63, 187–206. doi: 10.1007/s00018-005-5387-6, PMID: 16389456 PMC11136411

[ref189] Prats C. Fatjó-Vilas M. Penzol M. J. Kebir O. Pina-Camacho L. Demontis D.. (2022). Association and epistatic analysis of white matter related genes across the continuum schizophrenia and autism spectrum disorders: the joint effect of NRG1-ErbB genes. World J. Biol. Psychiatry 23, 208–218. doi: 10.1080/15622975.2021.1939155, PMID: 34338147

[ref190] Rajasekaran A. Shivakumar V. Kalmady S. V. Parlikar R. Chhabra H. Prabhu A.. (2020). Impact of NRG1 HapICE gene variants on digit ratio and dermatoglyphic measures in schizophrenia. Asian J. Psychiatr. 54:102363. doi: 10.1016/j.ajp.2020.102363, PMID: 33271685

[ref191] Rakic S. Zecevic N. (2000). Programmed cell death in the developing human telencephalon. Eur. J. Neurosci. 12, 2721–2734. doi: 10.1046/j.1460-9568.2000.00153.x 10971615

[ref192] Rashid M. Olson E. C. (2023). Delayed cortical development in mice with a neural specific deletion of β1 integrin. Front. Neurosci. 17:1158419. doi: 10.3389/fnins.2023.1158419 37250402 PMC10213249

[ref193] Ray M. T. Weickert S. C. Webster M. J. (2014). Decreased BDNF and TrkB mRNA expression in multiple cortical areas of patients with schizophrenia and mood disorders. Transl. Psychiatry 4:e389. doi: 10.1038/tp.2014.26, PMID: 24802307 PMC4035720

[ref194] Ray M. T. Weickert S. C. Wyatt E. Webster M. J. (2011). Decreased BDNF, trkB-TK+ and GAD67 mRNA expression in the hippocampus of individuals with schizophrenia and mood disorders. J. Psychiatry Neurosci. 36, 195–203. doi: 10.1503/jpn.100048, PMID: 21223646 PMC3080515

[ref195] Reis L. M. Houssin N. S. Zamora C. Abdul-Rahman O. Kalish J. M. Zackai E. H.. (2020). Novel variants in CDH2 are associated with a new syndrome including Peters anomaly. Clin. Genet. 97, 502–508. doi: 10.1111/cge.13660, PMID: 31650526 PMC7028510

[ref196] Ribasés M. Hervás A. Ramos-Quiroga J. A. Bosch R. Bielsa A. Gastaminza X.. (2008). Association study of 10 genes encoding neurotrophic factors and their receptors in adult and child attention-deficit/hyperactivity disorder. Biol. Psychiatry 63, 935–945. doi: 10.1016/j.biopsych.2007.11.004, PMID: 18179783

[ref197] Rice D. S. Curran T. (2001). Role of the reelin signaling pathway in central nervous system development. Ann. Rev. Neurosci. 24, 1005–1039. doi: 10.1146/annurev.neuro.24.1.1005, PMID: 11520926

[ref198] Roessler E. El-Jaick K. B. Dubourg C. Vélez J. I. Solomon B. D. Pineda-Alvarez D. E.. (2009). The mutational spectrum of holoprosencephaly-associated changes within the SHH gene in humans predicts loss-of-function through either key structural alterations of the ligand or its altered synthesis. Hum. Mutat. 30, E921–E935. doi: 10.1002/humu.21090, PMID: 19603532 PMC2772877

[ref199] Ross O. A. Braithwaite A. T. Skipper L. M. Kachergus J. Hulihan M. M. Middleton F. A.. (2008). Genomic investigation of alpha-synuclein multiplication and parkinsonism. Ann. Neurol. 63, 743–750. doi: 10.1002/ana.21380, PMID: 18571778 PMC3850281

[ref200] Rubenstein J. Rakic P. Chen B. Kenneth Y. Kwan H. T. (2020). “Chapter 22—Cajal–Retzius and subplate cells: transient cortical neurons and circuits with long-term impact” in Synapse development and maturation. ed. Cline J. C. (USA: Academic Press), 485–505.

[ref201] Sakai T. Sasaki T. Tatsumi M. Kunugi H. Hattori M. Nanko S. (1997). Schizophrenia and the ciliary neurotrophic factor (CNTF) gene: no evidence for association. Psychiatry Res. 71, 7–10. doi: 10.1016/S0165-1781(97)00039-5, PMID: 9247976

[ref202] Salatino-Oliveira A. Genro J. P. Polanczyk G. Zeni C. Schmitz M. Kieling C.. (2015). Cadherin-13 gene is associated with hyperactive/impulsive symptoms in attention/deficit hyperactivity disorder. Am. J. Med. Genet. B 168B, 162–169. doi: 10.1002/ajmg.b.32293, PMID: 25739828

[ref203] Sanders S. J. Ercan-Sencicek A. G. Hus V. Luo R. Murtha M. T. Moreno-De-Luca D.. (2011). Multiple recurrent de novo CNVs, including duplications of the 7q11.23 Williams syndrome region, are strongly associated with autism. Neuron 70, 863–885. doi: 10.1016/j.neuron.2011.05.002, PMID: 21658581 PMC3939065

[ref204] Sanders S. J. He X. Willsey A. J. Ercan-Sencicek A. G. Samocha K. E. Cicek A. E.. (2015). Insights into autism spectrum disorder genomic architecture and biology from 71 risk loci. Neuron 87, 1215–1233. doi: 10.1016/j.neuron.2015.09.016, PMID: 26402605 PMC4624267

[ref205] Sasi M. Vignoli B. Canossa M. Blum R. (2017). Neurobiology of local and intercellular BDNF signaling. Pflugers Arch. 469, 593–610. doi: 10.1007/s00424-017-1964-4, PMID: 28280960 PMC5438432

[ref206] Schwerd T. Twigg S. R. F. Aschenbrenner D. Manrique S. Miller K. A. Taylor I. B.. (2017). A biallelic mutation in IL6ST encoding the GP130 co-receptor causes immunodeficiency and craniosynostosis. J. Exp. Med. 214, 2547–2562. doi: 10.1084/jem.20161810, PMID: 28747427 PMC5584118

[ref207] Sears K. E. Gullapalli K. Trivedi D. Mihas A. Bukys M. A. Jensen J. (2022). Controlling neural territory patterning from pluripotency using a systems developmental biology approach. iScience 25:104133. doi: 10.1016/j.isci.2022.104133, PMID: 35434550 PMC9010746

[ref208] Segal R. A. Takahashi H. McKay R. D. (1992). Changes in neurotrophin responsiveness during the development of cerebellar granule neurons. Neuron 9, 1041–1052. doi: 10.1016/0896-6273(92)90064-K, PMID: 1463606

[ref209] Semba J. Akanuma N. Wakuta M. Tanaka N. Suhara T. (2004). Alterations in the expressions of mRNA for GDNF and its receptors in the ventral midbrain of rats exposed to subchronic phencyclidine. Brain Res. Mol. Brain Res. 124, 88–95. doi: 10.1016/j.molbrainres.2004.02.011, PMID: 15093689

[ref210] Semina E. Rubina K. Stepanova V. Tkachuk V. (2017). Involvement of the urokinase receptor and its endogenous ligands in the development of the brain and the formation of cognitive functions. Neurosci. Behav. Physiol. 48, 16–27. doi: 10.1007/s11055-017-0525-9

[ref211] Semina E. Rubina K. Sysoeva V. Rysenkova K. Klimovich P. Plekhanova O.. (2016). Urokinase and urokinase receptor participate in regulation of neuronal migration, axon growth and branching. Eur. J. Cell Biol. 95, 295–310. doi: 10.1016/j.ejcb.2016.05.003, PMID: 27324124

[ref212] Shahin T. Aschenbrenner D. Cagdas D. Bal S. K. Conde C. D. Garncarz W.. (2019). Selective loss of function variants in IL6ST cause hyper-IgE syndrome with distinct impairments of T-cell phenotype and function. Haematologica 104, 609–621. doi: 10.3324/haematol.2018.194233, PMID: 30309848 PMC6395342

[ref213] Shimazu K. Zhao M. Sakata K. Akbarian S. Bates B. Jaenisch R.. (2006). NT-3 facilitates hippocampal plasticity and learning and memory by regulating neurogenesis. Learn. Mem. 13, 307–315. doi: 10.1101/lm.76006, PMID: 16705139 PMC1475811

[ref214] Shmakova A. A. Balatskiy A. V. Kulebyakina M. A. Schaub T. Karagyaur M. N. Kulebyakin K. Y.. (2021). Urokinase receptor uPAR overexpression in mouse brain stimulates the migration of neurons into the cortex during embryogenesis. Russ. J. Dev. Biol. 52, 53–63. doi: 10.1134/S1062360421010069

[ref215] Silberberg G. Darvasi A. Pinkas-Kramarski R. Navon R. (2006). The involvement of ErbB4 with schizophrenia: association and expression studies. Am. J. Med. Genet. B 141B, 142–148. doi: 10.1002/ajmg.b.30275, PMID: 16402353

[ref216] Skibinska M. Kapelski P. Rajewska-Rager A. Szczepankiewicz A. Narozna B. Duda J.. (2019). Correlation of metabolic parameters, neurotrophin-3, and neurotrophin-4 serum levels in women with schizophrenia and first-onset depression. Nord. J. Psychiatry 73, 96–103. doi: 10.1080/08039488.2018.1563213, PMID: 30654674

[ref217] Smith T. F. Anastopoulos A. D. Garrett M. E. Arias-Vasquez A. Franke B. Oades R. D.. (2014). Angiogenic, neurotrophic, and inflammatory system SNPs moderate the association between birth weight and ADHD symptom severity. Am. J. Med. Genet. B 165B, 691–704. doi: 10.1002/ajmg.b.32275, PMID: 25346392

[ref218] Sokolov B. P. (1998). Expression of NMDAR1, GluR1, GluR7, and KA1 glutamate receptor mRNAs is decreased in frontal cortex of “neuroleptic-free” schizophrenics: evidence on reversible up-regulation by typical neuroleptics. J. Neurochem. 71, 2454–2464. doi: 10.1046/j.1471-4159.1998.71062454.x, PMID: 9832144

[ref219] Solé-Morata N. Baenas I. Etxandi M. Granero R. Forcales S. Gene M.. (2022). The role of neurotrophin genes involved in the vulnerability to gambling disorder. Sci. Rep. 12:6925. doi: 10.1038/s41598-022-10391-w 35484167 PMC9051155

[ref220] Souza R. P. Romano-Silva M. A. Lieberman J. A. Meltzer H. Y. MacNeil L. T. Culotti J. G.. (2010). Genetic association of the GDNF alpha-receptor genes with schizophrenia and clozapine response. J. Psychiatr. Res. 44, 700–706. doi: 10.1016/j.jpsychires.2010.01.002, PMID: 20116071

[ref221] Stiles J. Jernigan T. L. (2010). The basics of brain development. Neuropsychol. Rev. 20, 327–348. doi: 10.1007/s11065-010-9148-4, PMID: 21042938 PMC2989000

[ref222] Su L. Ling W. Jiang J. Hu J. Fan J. Guo X.. (2016). Association of EPHB1 rs11918092 and EFNB2 rs9520087 with psychopathological symptoms of schizophrenia in Chinese Zhuang and Han populations. Asia Pac. Psychiatry 8, 306–308. doi: 10.1111/appy.12241, PMID: 27028544

[ref223] Su Y. Yang L. Li Z. Wang W. Xing M. Fang Y.. (2021). The interaction of ASAH1 and NGF gene involving in neurotrophin signaling pathway contributes to schizophrenia susceptibility and psychopathology. Prog. Neuro-Psychopharmacol. Biol. Psychiatry 104:110015. doi: 10.1016/j.pnpbp.2020.110015, PMID: 32569620

[ref224] Suchanek-Raif R. Raif P. Kowalczyk M. Paul-Samojedny M. Zielińska A. Kucia K.. (2020). An analysis of five TrkB Gene polymorphisms in schizophrenia and the interaction of its haplotype with rs6265 BDNF Gene polymorphism. Dis. Markers 2020:4789806. doi: 10.1155/2020/4789806, PMID: 32351633 PMC7174942

[ref225] Tabata H. Nakajima K. (2003). Multipolar migration: the third mode of radial neuronal migration in the developing cerebral cortex. J. Neurosci. 23, 9996–10001. doi: 10.1523/JNEUROSCI.23-31-09996.2003, PMID: 14602813 PMC6740853

[ref226] Tamminga C. A. Thaker G. K. Buchanan R. Kirkpatrick B. Alphs L. D. Chase T. N.. (1992). Limbic system abnormalities identified in schizophrenia using positron emission tomography with fluorodeoxyglucose and neocortical alterations with deficit syndrome. Arch. Gen. Psychiatry 49, 522–530. doi: 10.1001/archpsyc.1992.01820070016003, PMID: 1627043

[ref227] Tanaka Y. Ujike H. Fujiwara Y. Takeda T. Takehisa Y. Kodama M.. (1998). Schizophrenic psychoses and the CNTF null mutation. Neuroreport 9, 981–983. doi: 10.1097/00001756-199804200-00005, PMID: 9601653

[ref228] Tartter M. Hammen C. Bower J. E. Brennan P. A. Cole S. (2015). Effects of chronic interpersonal stress exposure on depressive symptoms are moderated by genetic variation at IL6 and IL1β in youth. Brain Behav. Immun. 46, 104–111. doi: 10.1016/j.bbi.2015.01.003, PMID: 25596176 PMC4515110

[ref229] Tate D. F. Shenton M. E. Bigler E. D. (2012). Introduction to the brain imaging and behavior special issue on neuroimaging findings in mild traumatic brain injury. Brain Imaging Behav. 6, 103–107. doi: 10.1007/s11682-012-9185-0 22706729

[ref230] Tate D. F. Wilde E. A. Bouix S. McCauley S. R. (2015). Introduction to the brain imaging and behavior special issue: mild traumatic brain injury among active duty service members and veterans. Brain Imaging Behav. 9, 355–357. doi: 10.1007/s11682-015-9445-x 26319533

[ref231] Taylor H. Campbell J. Nobes C. D. (2017). Ephs and ephrins. Curr. Biol. 27, R90–R95. doi: 10.1016/j.cub.2017.01.003, PMID: 28171762

[ref232] Terry A. V. Jr. Gearhart D. A. Pillai A. Zhang G. Bartlett M. G. (2010). Chronic antipsychotic treatment: protracted decreases in phospho-TrkA levels in the rat hippocampus. Int. J. Neuropsychopharmacol. 13, 799–805. doi: 10.1017/S1461145709991040, PMID: 20059802 PMC4400726

[ref233] The UniProt Consortium (2023a). UniProt: the Universal Protein Knowledgebase. Sonic hedgehog protein. Available at: https://www.UniProt.org/UniProtkb/Q15465/variant-viewer (Accessed October 25, 2023).

[ref234] The UniProt Consortium (2023b). UniProt: the Universal Protein Knowledgebase. Chordin. Available at: https://www.UniProt.org/UniProtkb/Q9H2X0/variant-viewer (Accessed October 25, 2023).

[ref235] The UniProt Consortium (2023c). UniProt: the Universal Protein Knowledgebase. Interleukin-6 receptor subunit beta. Available at: https://www.UniProt.org/UniProtkb/P40189/variant-viewer (Accessed October 25, 2023).

[ref236] The UniProt Consortium (2023d). UniProt: the Universal Protein Knowledgebase. Receptor tyrosine-protein kinase erbB-2. Available at: https://www.UniProt.org/UniProtkb/P04626/variant-viewer (Accessed October 25, 2023).

[ref237] The UniProt Consortium (2023e). UniProt: the Universal Protein Knowledgebase. Receptor tyrosine-protein kinase erbB-3. Available at: https://www.UniProt.org/UniProtkb/P21860/variant-viewer (Accessed October 25, 2023).

[ref238] The UniProt Consortium (2023f). UniProt: the Universal Protein Knowledgebase. Receptor tyrosine-protein kinase erbB-4. Available at: https://www.UniProt.org/UniProtkb/Q15303/variant-viewer (Accessed October 25, 2023).

[ref239] The UniProt Consortium (2023g). UniProt: the Universal Protein Knowledgebase. Ephrin-B1. Available at: https://www.UniProt.org/UniProtkb/P98172/variant-viewer (Accessed October 25, 2023). 10.1093/nar/gky092 PMC5861450 29425356

[ref240] The UniProt Consortium (2023h). UniProt: the Universal Protein Knowledgebase. Ephrin type-A receptor 4. Available at: https://www.UniProt.org/UniProtkb/P54764/variant-viewer (Accessed October 25, 2023).

[ref241] The UniProt Consortium (2023i). UniProt: the Universal Protein Knowledgebase. Ephrin type-A receptor 5. Available at: https://www.UniProt.org/UniProtkb/P54756/variant-viewer (Accessed October 25, 2023).

[ref242] The UniProt Consortium (2023j). UniProt: the Universal Protein Knowledgebase. Ephrin type-B receptor 4. Available at: https://www.UniProt.org/UniProtkb/P54760/variant-viewer (Accessed October 25, 2023).

[ref243] The UniProt Consortium (2023k). UniProt: the Universal Protein Knowledgebase. Semaphorin-6B. Available at: https://www.UniProt.org/UniProtkb/Q9H3T3/variant-viewer (Accessed October 25, 2023).

[ref244] The UniProt Consortium (2023l). UniProt: the Universal Protein Knowledgebase. Plexin-A1. Available at: https://www.UniProt.org/UniProtkb/Q9UIW2/variant-viewer (Accessed October 25, 2023).

[ref245] The UniProt Consortium (2023m). UniProt: the Universal Protein Knowledgebase. Plexin-A3. Available at: https://www.UniProt.org/UniProtkb/P51805/variant-viewer (Accessed October 25, 2023).

[ref246] The UniProt Consortium (2023n). UniProt: the Universal Protein Knowledgebase. Netrin-1. Available at: https://www.UniProt.org/UniProtkb/O95631/variant-viewer (Accessed October 25, 2023).

[ref247] The UniProt Consortium (2023o). UniProt: the Universal Protein Knowledgebase. Netrin-G2. Available at: https://www.UniProt.org/UniProtkb/Q96CW9/variant-viewer (Accessed October 25, 2023).

[ref248] The UniProt Consortium (2023p). UniProt: the Universal Protein Knowledgebase. Netrin receptor DCC. Available at: https://www.UniProt.org/UniProtkb/P43146/variant-viewer (Accessed October 25, 2023).

[ref249] The UniProt Consortium (2023q). UniProt: the Universal Protein Knowledgebase. Roundabout homolog 1. Available at: https://www.UniProt.org/UniProtkb/Q9Y6N7/variant-viewer (Accessed October 25, 2023).

[ref250] The UniProt Consortium (2023s). UniProt: the Universal Protein Knowledgebase. Protocadherin-12. Available at: https://www.UniProt.org/UniProtkb/Q9NPG4/variant-viewer (Accessed October 25, 2023).

[ref251] Thoenen H. (1995). Neurotrophins and neuronal plasticity. Science 270, 593–598. doi: 10.1126/science.270.5236.593, PMID: 7570017

[ref252] Thoenen H. Bandtlow C. Heumann R. (1987). The physiological function of nerve growth factor in the central nervous system: comparison with the periphery. Rev. Physiol. Biochem. Pharmacol. 109, 145–178. doi: 10.1007/BFb0031026, PMID: 3317757

[ref253] Thompson C. L. Ng L. Menon V. Martinez S. Lee C. K. Glattfelder K.. (2014). A high-resolution spatiotemporal atlas of gene expression of the developing mouse brain. Neuron 83, 309–323. doi: 10.1016/j.neuron.2014.05.033, PMID: 24952961 PMC4319559

[ref254] Ting E. Y. Yang A. C. Tsai S. J. (2020). Role of interleukin-6 in depressive disorder. Int. J. Mol. Sci. 21:2194. doi: 10.3390/ijms21062194, PMID: 32235786 PMC7139933

[ref255] Torun Y. T. Güney E. Iseri E. (2015). “Structural and functional brain imaging in autism spectrum disorders” in Autism spectrum disorder. ed. Fitzgerald M. (United Kingdom: IntechOpen), 378.

[ref256] Toudji I. Toumi A. Chamberland É. Rossignol E. (2023). Interneuron odyssey: molecular mechanisms of tangential migration. Front. Neural Circuits 17:1256455. doi: 10.3389/fncir.2023.1256455, PMID: 37779671 PMC10538647

[ref257] Troilo H. Zuk A. V. Tunnicliffe R. B. Wohl A. P. Berry R. Collins R. F.. (2014). Nanoscale structure of the BMP antagonist chordin supports cooperative BMP binding. Proc. Natl. Acad. Sci. U.S.A. 111, 13063–13068. doi: 10.1073/pnas.1404166111, PMID: 25157165 PMC4246984

[ref258] Tsai S. J. (2004). Down-regulation of the Trk-B signal pathway: the possible pathogenesis of major depression. Med. Hypotheses 62, 215–218. doi: 10.1016/S0306-9877(03)00299-8, PMID: 14962629

[ref259] Tsai S. J. (2007). The P11, tPA/plasminogen system and brain-derived neurotrophic factor: implications for the pathogenesis of major depression and the therapeutic mechanism of antidepressants. Med. Hypotheses 68, 180–183. doi: 10.1016/j.mehy.2006.06.005, PMID: 16890384

[ref260] Tsai S. J. (2018). Critical issues in BDNF Val66Met genetic studies of neuropsychiatric disorders. Front. Mol. Neurosci. 11:156. doi: 10.3389/fnmol.2018.00156, PMID: 29867348 PMC5962780

[ref261] Turner J. R. Ray R. Lee B. Everett L. Xiang J. Jepson C.. (2014). Evidence from mouse and man for a role of neuregulin 3 in nicotine dependence. Mol. Psychiatry 19, 801–810. doi: 10.1038/mp.2013.104, PMID: 23999525 PMC3877725

[ref262] Udina M. Moreno-España J. Navinés R. Giménez D. Langohr K. Gratacòs M.. (2013). Serotonin and interleukin-6: the role of genetic polymorphisms in IFN-induced neuropsychiatric symptoms. Psychoneuroendocrinology 38, 1803–1813. doi: 10.1016/j.psyneuen.2013.03.007, PMID: 23571152

[ref263] Uesaka N. Uchigashima M. Mikuni T. Nakazawa T. Nakao H. Hirai H.. (2014). Retrograde semaphorin signaling regulates synapse elimination in the developing mouse brain. Science 344, 1020–1023. doi: 10.1126/science.1252514, PMID: 24831527

[ref264] Valian N. Ahmadiani A. Dargahi L. (2017). Escalating methamphetamine regimen induces compensatory mechanisms, mitochondrial biogenesis, and GDNF expression, in substantia nigra. J. Cell. Biochem. 118, 1369–1378. doi: 10.1002/jcb.25795, PMID: 27862224

[ref265] van Schijndel J. E. van Loo K. M. van Zweeden M. Djurovic S. Andreassen O. A. Hansen T.. (2009). Three-cohort targeted gene screening reveals a non-synonymous TRKA polymorphism associated with schizophrenia. J. Psychiatr. Res. 43, 1195–1199. doi: 10.1016/j.jpsychires.2009.04.006, PMID: 19435634

[ref266] van Schijndel J. E. van Zweeden M. van Loo K. M. J. Djurovic S. Andreassen O. A. Hansen T.. (2011). Dual association of a TRKA polymorphism with schizophrenia. Psychiatr. Genet. 21, 125–131. doi: 10.1097/YPG.0b013e3283437194 21317683

[ref267] Virgos C. Martorell L. Valero J. Figuera L. Civeira F. Joven J.. (2001). Association study of schizophrenia with polymorphisms at six candidate genes. Schizophr. Res. 49, 65–71. doi: 10.1016/S0920-9964(00)00106-7, PMID: 11343865

[ref268] Walsh T. McClellan J. M. McCarthy S. E. Addington A. M. Pierce S. B. Cooper G. M.. (2008). Rare structural variants disrupt multiple genes in neurodevelopmental pathways in schizophrenia. Science 320, 539–543. doi: 10.1126/science.1155174, PMID: 18369103

[ref269] Wang Y. C. Chen J. Y. Chen M. L. Chen C. H. Lai I. C. Chen T. T.. (2008). Neuregulin 3 genetic variations and susceptibility to schizophrenia in a Chinese population. Biol. Psychiatry 64, 1093–1096. doi: 10.1016/j.biopsych.2008.07.012, PMID: 18708184

[ref270] Wannemacher K. M. Wang L. Zhu L. Brass L. F. (2011). The role of semaphorins and their receptors in platelets: lessons learned from neuronal and immune synapses. Platelets 22, 461–465. doi: 10.3109/09537104.2011.561891, PMID: 21668292 PMC3151319

[ref271] Wearne T. A. Cornish J. L. (2018). A comparison of methamphetamine-induced psychosis and schizophrenia: a review of positive, negative, and cognitive symptomatology. Front. Psychiatry 9:491. doi: 10.3389/fpsyt.2018.00491 30364176 PMC6191498

[ref272] Weickert C. S. Ligons D. L. Romanczyk T. Ungaro G. Hyde T. M. Herman M. M.. (2005). Reductions in neurotrophin receptor mRNAs in the prefrontal cortex of patients with schizophrenia. Mol. Psychiatry 10, 637–650. doi: 10.1038/sj.mp.4001678, PMID: 15940304

[ref273] Weng Q. Wang J. Wang J. He D. Cheng Z. Zhang F.. (2019). Single-cell transcriptomics uncovers glial progenitor diversity and cell fate determinants during development and gliomagenesis. Cell Stem Cell 24, 707–723. doi: 10.1016/j.stem.2019.03.006, PMID: 30982771 PMC6669001

[ref274] Whittemore S. R. Seiger Å. (1987). The expression, localization and functional significance of β-nerve growth factor in the central nervous system. Brain Res. Rev. 12, 439–464. doi: 10.1016/0165-0173(87)90008-7, PMID: 2825921

[ref275] Wodarz A. Huttner W. B. (2003). Neurogenic radial glial cells in reptile, rodent and human: from mitosis to migration. Cereb. Cortex 13, 550–559. doi: 10.1093/cercor/13.6.550, PMID: 12764028

[ref276] World Health Organization (2022). World mental health report: transforming mental health for all. Available at: https://www.who.int/publications/i/item/9789240049338 (Accessed October 25, 2023).

[ref277] Xu M. Q. St Clair D. Feng G. Y. Lin Z. G. He G. Li X.. (2008). BDNF gene is a genetic risk factor for schizophrenia and is related to the chlorpromazine-induced extrapyramidal syndrome in the Chinese population. Pharmacogenet. Genomics 18, 449–457. doi: 10.1097/FPC.0b013e3282f85e26 18408624

[ref278] Yamagishi S. Bando Y. Sato K. (2021). Involvement of netrins and their receptors in neuronal migration in the cerebral cortex. Front. Cell Dev. Biol. 8:590009. doi: 10.3389/fcell.2020.590009, PMID: 33520982 PMC7843923

[ref279] Yan Q. Rosenfeld R. D. Matheson C. R. Hawkins N. Lopez O. T. Bennett L.. (1997). Expression of brain-derived neurotrophic factor protein in the adult rat central nervous system. Neuroscience 78, 431–448. doi: 10.1016/S0306-4522(96)00613-6, PMID: 9145800

[ref280] Yang J. S. Wei H. X. Chen P. P. Wu G. (2018). Roles of Eph/ephrin bidirectional signaling in central nervous system injury and recovery. Exp. Ther. Med. 15, 2219–2227. doi: 10.4103/1673-5374.235217 29456630 PMC5795627

[ref281] Yeh Y. W. Kuo S. C. Chen C. Y. Liang C. S. Ho P. S. Yen C. H.. (2015). Harm avoidance involved in mediating the association between nerve growth factor (NGF) gene polymorphisms and antidepressant efficacy in patients with major depressive disorder. J. Affect. Disord. 183, 187–194. doi: 10.1016/j.jad.2015.05.012, PMID: 26021968

[ref282] Yokota Y. Gashghaei H. T. Han C. Watson H. Campbell K. J. Anton E. S. (2007). Radial glial dependent and independent dynamics of interneuronal migration in the developing cerebral cortex. PLoS One 2:e794. doi: 10.1371/journal.pone.0000794, PMID: 17726524 PMC1950908

[ref283] Zakharyan R. Atshemyan S. Gevorgyan A. Boyajyan A. (2014). Nerve growth factor and its receptor in schizophrenia. BBA Clin. 1, 24–29. doi: 10.1016/j.bbacli.2014.05.001, PMID: 26675984 PMC4633968

[ref284] Zambon A. A. Cecchetti G. Caso F. Santangelo R. Baldoli C. Natali Sora M. G.. (2017). Primary progressive multiple sclerosis presenting with severe predominant cognitive impairment and psychiatric symptoms: a challenging case. Mult. Scler. 23, 1558–1561. doi: 10.1177/1352458517702550, PMID: 28401768

[ref285] Zhang L. Qi Z. Li J. Li M. Du X. Wang S.. (2021). Roles and mechanisms of axon-guidance molecules in Alzheimer’s disease. Mol. Neurobiol. 58, 3290–3307. doi: 10.1007/s12035-021-02311-2, PMID: 33675023

[ref286] Zhang R. Zhong N. N. Liu X. G. Yan H. Qiu C. Han Y.. (2010). Is the EFNB2 locus associated with schizophrenia? Single nucleotide polymorphisms and haplotypes analysis. Psychiatry Res. 180, 5–9. doi: 10.1016/j.psychres.2010.04.037, PMID: 20483485

[ref287] Zhao L. Hou B. Ji L. Ren D. Yuan F. Liu L.. (2022). NGFR gene and single nucleotide polymorphisms, rs2072446 and rs11466162, playing roles in psychiatric disorders. Brain Sci. 12:1372. doi: 10.3390/brainsci12101372, PMID: 36291307 PMC9599857

[ref288] Zheng Z. H. Lin X. C. Lu Y. Cao S. R. Liu X. K. Lin D.. (2023). Harmine exerts anxiolytic effects by regulating neuroinflammation and neuronal plasticity in the basolateral amygdala. Int. Immunopharmacol. 119:110208. doi: 10.1016/j.intimp.2023.110208, PMID: 37150016

[ref289] Zhou X. Xiao Q. Xie L. Yang F. Wang L. Tu J. (2019). Astrocyte, a promising target for mood disorder interventions. Front. Mol. Neurosci. 12:136. doi: 10.3389/fnmol.2019.00136, PMID: 31231189 PMC6560156

