# Supplementary material for: Morphogenetic theory of mental and cognitive disorders: the role of neurotrophic and guidance molecules
Source: Front Mol Neurosci. 2024 Apr 3;17:1361764. doi: 10.3389/fnmol.2024.1361764 (PMC11027769; doi:10.3389/fnmol.2024.1361764)
Supplement: Supplementary file 1 [file Data_Sheet_1.docx]

Supplementary Material

**Morphogenetic theory of mental and cognitive disorders: the role of neurotrophiс and guidance molecules**

**Alexandra Primak^1^**^,^***, Kirill Bozov^1^**^,^***, Kseniya Rubina^1^, Stalik Dzhauari^1^, Elena Neyfeld^1,2^, Maria Illarionova^1^, Ekaterina Semina^1^, Dmitriy Sheleg^1,2^, Vsevolod Tkachuk^1,3^, Maxim Karagyaur^1,3,×^**

^1^Faculty of Medicine, Lomonosov Moscow State University, 27/1, Lomonosovsky Ave., 119192,

Moscow, Russia; info@fbm.msu.ru

^2^Federal State Budgetary Educational Institution of the Higher Education “A.I. Yevdokimov Moscow State University of Medicine and Dentistry” of the Ministry of Healthcare of the Russian Federation, 4, Dolgorukovskaya St., 127006 Moscow, Russia; mail@msmsu.ru

^3^Institute for Regenerative Medicine, Medical Research and Education Center, Lomonosov

Moscow State University, 27/10, Lomonosovsky Ave., 119192, Moscow, Russia;

info@irm.msu.ru

* - These authors contributed equally to this work and share first authorship

**^×^ - Correspondence:**Maxim Karagyaur
[m.karagyaur@mail.ru](mailto:m.karagyaur@mail.ru)

# Supplementary Figure


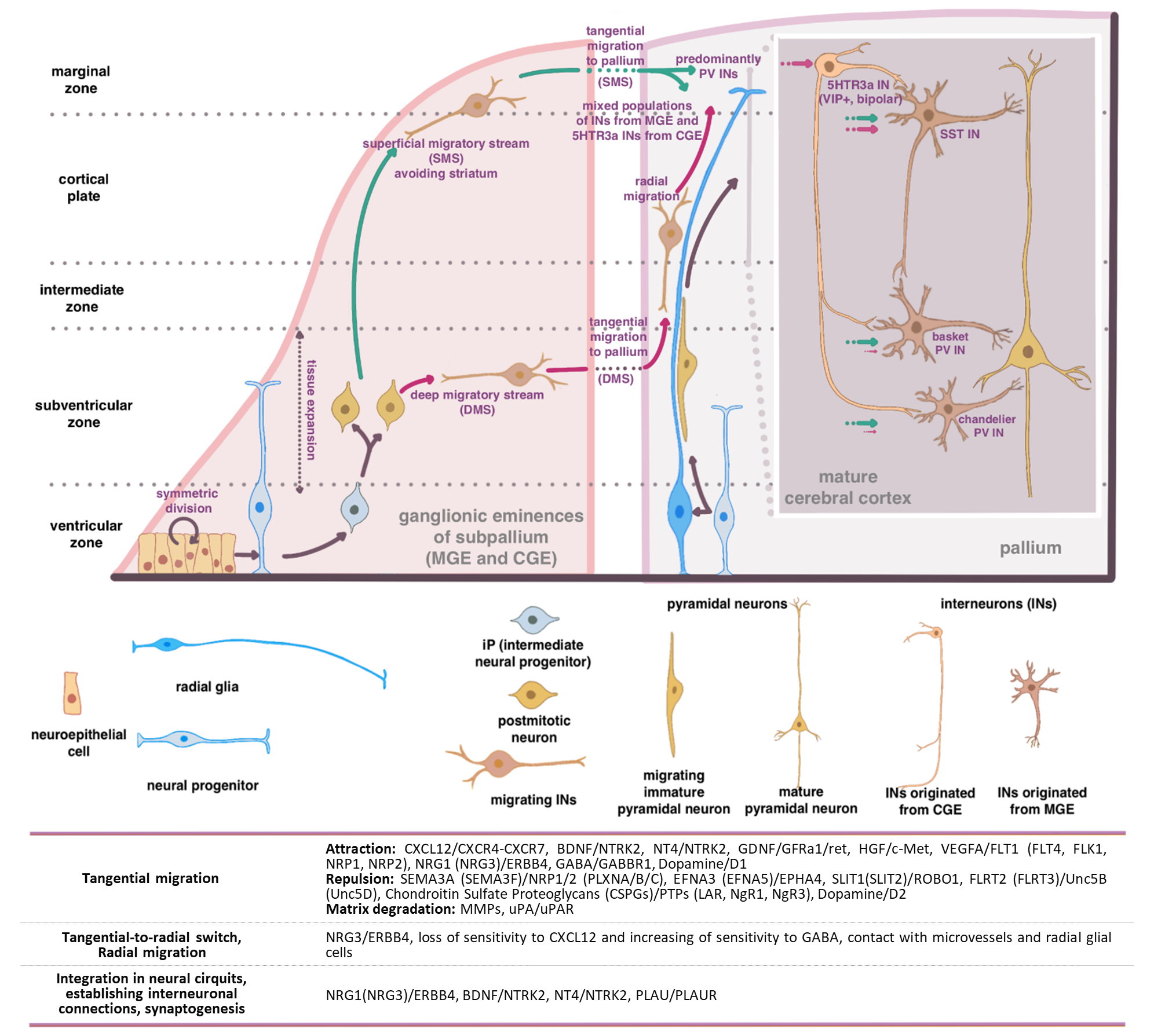


**Figure S1.** The crucial stages of interneuron (**IN**) origin and development and the main classes of molecules involved in these processes. **CGE** - caudal ganglionic eminence, **MGE** - medial ganglionic eminence, **PV** - parvalbumin-positive INs, **SST** - somatostatin-positive INs.

# Supplementary Table

**Supplementary Table S1. Previously described genomic variants of the brain morphogenesis genes associated with the predisposition to psychiatric and cognitive disorders.**

| **Gene** | **Description** | **Function** | **Variant** | **Domain** | **Condition** | **Sampled population** | **Association (yes/no)** | **Reference** |
| --- | --- | --- | --- | --- | --- | --- | --- | --- |
| *ВDNF* | Brain-Derived Neurotrophic Factor | stimulates proliferation, survival, migration of neural progenitors and mature neurons, stimulates neurogenesis, synaptogenesis, stabilizes the interneuron connections in the brain | rs1103010101-A (intr) |  | schizophrenia  (negative symptoms) | Chinese | yes | (Ping et al., 2022) |
|  |  |  | AAC haplotype:  rs11030101-A (intr),  rs2030324-A (intr) and  rs6265-C (V64V) |  | schizophrenia | Chinese | yes | (Ping et al., 2022) |
|  |  |  | rs6265-C (V64V) or  rs2030324-A (intr) |  | schizophrenia | Chinese | no | (Ping et al., 2022) |
|  |  |  | rs6265-C (V64V) |  | ADHD | Chinese | yes | (Ping et al., 2022) |
|  |  |  | rs769727156-T (G198D) |  | schizophrenia-related psychosis | American | yes | (Kranz et al., 2015) |
|  |  |  | rs6265-T (V66M),  rs12273539-T (intr),  rs11030103-G (intr),  rs28722151-G (intr),  rs41282918-T (3’UTR) or  rs11030101-T (intr) |  | depressive disorder | Mexican Americans | yes | (Licinio et al., 2009) |
|  |  |  | rs56164415 (C270T) |  | Alzheimer's disease | Japan | yes | (Kunugi et al., 2001) |
| *CDH11* | Cadherin 11 | mediates calcium-dependent inter cellular adhesion; participates in the navigation of neurites and migrating cells | VAR_086894 (W55S) or VAR_086895 (E140K) | Extracellular domain | Teebi Hypertelorism Syndrome 2 (TBHS2), developmental delay, mental retardation |  | yes | (The UniProt Consortium, 2023t; Li D. et al., 2021) |
| *CDH13* | Cadherin 13 | mediates calcium-dependent inter cellular adhesion; participates in the navigation of neurites and migrating cells; a receptor for low-density lipoprotein (LDL)? | rs11647188-G (intr),  rs6565113-C (intr) or  rs11150556-C (intr) |  | Attention deficit hyperactivity disorder (ADHD) and autism spectrum disorders (ASD) |  | yes | (Lasky-Su et al., 2008) |
|  |  |  | rs6565113-C (intr) |  | ADHD |  | yes | (Lasky-Su et al., 2008) |
| *CDH15* | Cadherin 15 | mediates calcium-dependent inter cellular adhesion | rs567903921-C (V8L),  rs121434539-T (R60C) or  rs121434540-T (R92W) | Extracellular domain,  EC1 | Mental retardation, autosomal dominant 3 (MRD3) | Indian | yes | (The UniProt Consortium, 2023u; Bhalla et al., 2008) |
| *CDH2* | Cadherin 2 | mediates calcium-dependent inter cellular adhesion; is expressed on the surface of radial glial cells - participates in radial migration of neural progenitors | VAR_087507 (H150Y) or  VAR_087507 (H150Y) |  | Attention deficit-hyperactivity disorder-8 (ADHD8) |  | yes | (The UniProt Consortium, 2023r;Halperin et al., 2021) |
|  |  |  | rs2013111940 -T (V162D),  rs1599017933-T (D353N) or  rs1599011050-T (D597N) | Extracellular domain 1  Extracellular domain 3 | Agenesis of corpus callosum, cardiac, ocular, and genital syndrome (ACOGS) |  | yes | (The UniProt Consortium, 2023r; Reis et al., 2020) |
| *CDH5* | Cadherin 5 | mediates calcium-dependent inter cellular adhesion | rs1596937199-A (V89M) | Extracellular domain 1 | Marfanoid habitus and intellectual disability |  | yes | (The UniProt Consortium, 2023v) |
| *CDH7* | Cadherin 7 | mediates calcium-dependent inter cellular adhesion; mediates synapse formation between PN neurons and granule neurons | rs12605720-G (intr) or  rs1444067-T (promotor) |  | depression |  | yes | (Li X. et al., 2014) |
|  |  |  | rs1444067-T (promotor) |  | bipolar disorder |  | yes | (Li X. et al., 2014) |
| *CNTF* | Ciliary neurotrophic factor | supports the survival and proliferation of hippocampal neurons, medial septal neurons | rs1800169 (FS63Stop) |  | schizoaffective disorder and better susceptibility to iloperidone (antipsychotic) therapy |  | yes | (Lavedan et al., 2008; Okahisa et al., 2010) |
|  |  |  | rs7036351-T (promoter) |  | drug addiction | Spanish | yes | (Okahisa et al., 2010) |
|  |  |  | rs3763614-G (intr) |  | gambling addiction |  | yes | (Solé-Morata et al., 2022) |
|  |  |  | rs7036351-C (promoter) or rs3763613-G (intr) |  | ADHD | Spanish | yes | (Ribasés et al., 2008) |
|  |  |  | rs10758268-T (intr) or  rs7044318-T (intr) |  | ADHD | populations from Europe and Israel | yes | (Smith et al., 2014) |
| *DCC* | Deleted In Colorectal Carcinoma (Netrin receptor) | [axon guidance](https://en.wikipedia.org/wiki/Axon_guidance) receptor that responds to [netrin-1](https://en.wikipedia.org/wiki/Netrin-1) | rs754914260-T (R275Stop),  rs7970445519-A (S126Stop) or  rs1180126622-T (R215Stop) | Ig-like C2-type 3 domain  Ig-like C2-type 1 domain  Ig-like C2-type 2 domain | Mirror movements 1 condition (MRMV1) |  | yes | (The UniProt Consortium, 2023p) |
|  |  |  | rs1555682265 (V263missing) | Ig-like C2-type 3 domain | Gaze palsy |  | yes | (The UniProt Consortium, 2023p) |
| *DCHS1* | Dachsous Cadherin-Related 1 | cell–cell adhesion molecule | rs483352917-A (G835Stop) or  rs483352919-A (N2370I) |  | Van Maldergem syndrome (VMLDS1) |  | yes | (Cappello et al., 2013) |
| *DCHS2* | Dachsous Cadherin-Related 2 | cell–cell adhesion molecule | rs1466662-A (intr) |  | mild cognitive impairment | Brazilian | yes | (Vieira et al., 2016) |
| *EFNB1* | Ephrin B1 | guidance molecules,  involved in axon growth | VAR_023127 (P27R),  rs104894803-A (W37Stop) or  rs104894801-T (P54L) |  | craniofrontonasal syndrome |  | yes | (The UniProt Consortium, 2023g) |
| *EFNB2* | Ephrin B2 |  | rs9520087-T (3'UTR),  TTC haplotype:  rs9520087-T (3’UTR),  rs11069646-T (intr) and  rs8000078-C (intr) |  | schizophrenia | Chinese (Han) | yes | (Zhang et al., 2010) |
| *EPHA4* | Ephrin type-A receptor 4 | regulate the radial and tangential migration, axonal outgrowth and pathfinding, topographic mapping, axon fasciculation, synapse maturation and vascular formation in the developing nervous system | rs155356868456-T (A748T) | Protein kinase domain | atypical cerebral palsy |  | yes | (The UniProt Consortium, 2023h) |
| *EPHA5* | Ephrin type-A receptor 5 |  | rs200932017-C (D348G) | Extracellular domain | astrocytoma |  | yes | (The UniProt Consortium, 2023i) |
| *EPHA6* | Ephrin type-A receptor 6 |  | rs727229-G (intr) |  | schizophrenia | Japanese | no | (Ikeda et al., 2010) |
| *EPHB1* | Ephrin type-B receptor 1 | Ephrin receptors, topographic mapping, axon fasciculation, synapse maturation and vascular formation in the developing nervous system | rs11918092 (promoter) |  | schizophrenia  (positive symptoms) | Chinese (Han) | yes | (Su et al., 2016) |
|  |  |  | rs140725416-C (F151S),  rs150028142-A (D375N),  rs145366861-A (D577N),  rs149160192-T (R637C),  rs143309901-T (R905C) or  rs561862 70-Т (Т981М) | Eph LBD  Fibronectin type-III 1 domain  Protein kinase domain | schizophrenia | Japanese | no | (Kushima et al., 2012) |
| *EPHB2* | Ephrin type-B receptor 2 |  | rs9520087-T (3'UTR) |  | schizophrenia  (negative symptoms) | Chinese  (Zhuang) | yes | (Su et al., 2016) |
| *EPHB4* | Ephrin type-B receptor 4 |  | rs1584666961-T (W130Stop),  rs1562973541-CA (V211Missing) or  rs1484547615-A (G375Stop) | Eph LBD  Fibronectin type-III 1 domain | capillary malformation-arteriovenous malformation 2 syndrome |  | yes | (The UniProt Consortium, 2023j) |
|  |  |  | rs707284-G (intr),  rs839523-G (intr) or  rs7598440-A (intr) |  | schizophrenia | Jewish | yes | (Silberberg et al., 2006) |
|  |  |  | rs2289086-T (intr) |  | schizophrenia | Chinese (Han) | yes | (Lu et al., 2010) |
|  |  |  | rs3748962-G (V1065V) |  | schizophrenia | African American | yes | (Nicodemus et al., 2006) |
| *GDNF* | Glial cell line-derived neurotrophic factor | stimulates survival and functioning of dopaminergic and motor neurons | (AGG)n |  | psychiatric disorders | Japanese | no | (Ma et al., 2013) |
|  |  |  | 15 or more AGG repeats |  | psychiatric disorders (reduced risk) | Italian | yes | (Michelato et al., 2004) |
|  |  |  | (AGG)n,  rs2910709-C/G/T (3’downstream),  rs2973050-C/G/T (intr),  rs884344-C (intr),  rs2910702-A/T (intr),  rs2216710-T (intr) or  rs3812047-G/T (intr) |  | psychiatric disorders | Hungarian | no | (Kotyuk et al., 2013) |
| *GFRA1* | GDNF Family Receptor Alpha 1 | bind glial neurotrophic factor GDNF and Artemine, respectively; form the signal-transducing complexes with IL6ST | rs11197557-T (intr) |  | schizophrenia |  | yes | (Souza et al., 2010) |
| *GFRA3* | GDNF Family Receptor Alpha 3 |  | rs11242417-G (intr) |  | schizophrenia |  | yes | (Souza et al., 2010) |
| *IL6* | Interleukin 6 | maintains the survival of neural progenitors and controls neuron differentiation in the central and peripheral nervous system | rs1800795-C (promoter) |  | depression | Australian and Spanish | yes | (Tartter et al., 2015, Udina et al., 2013) |
|  |  |  | rs1800795-C (promoter) |  | increased sensitivity to pain and stress perception | Hungarian (depressed patients) | yes | (Kovacs et al., 2016) |
|  |  |  | rs1800796-C (promoter) |  | depression and chronic schizophrenia | Chinese | yes | (Lu et al., 2023) |
| *IL6R* | Interleukin 6 receptor | binds Interleukin 6 and forms the signal-transducing complex with IL6ST | rs2228145-C (D358A) or  rs4537545-T (intr) |  | schizophrenia psychosis  severe depression | European | yes | (Khandaker et al., 2018; Kapelski et al., 2015) |
|  |  |  | rs57569414 (intr) |  | associated with the severity of depression and its resistance to therapy | Spanish | yes | (Draganov et al., 2019) |
| *IL6ST* | Interleukin 6 signal transducer | provides signaling upon binding of the cytokines CNTF, LIF, OSM and IL-6 to the corresponding receptor subunit; supports proliferation of neural progenitors of the hippocampus, forebrain and spinal cord and prevents their premature differentiation | rs1580809257-A (N404Y),  rs1381682599-G (A517P),  rs1580801731-A (P498L),  VAR_086953,  VAR_086954,  VAR_086955 or  VAR_086950 | Fibronectin type-III 3  Fibronectin type-III 4  Fibronectin type-III 4 | immunodeficiencies and impaired brain tissue development, causing autism and mental retardation |  | yes | (Schwerd et al., 2017; Shahin et al., 2019; Materna-Kiryluk et al., 2021) |
| *KIDINS220* | Kinase D Interacting Substrate 220 | promotes a prolonged MAP-kinase signaling by neurotrophins | rs748531715-C (H1085R) | TOPO_DOM 707-1771  Cytoplasmic domain | schizophrenia (increased risk of severe paranoid hallucinations) |  | yes | (Encinas et al., 2000; Lasky-Su et al., 2008) |
| *NGF* | Nerve Growth Factor | stimulates proliferation, survival, migration of neural progenitors and mature neurons, stimulates neurogenesis, synaptogenesis, stabilizes the interneuron connections in the brain | rs6330-A (A35V),  haplotype:  rs12760036-C (intr) and rs4839435-A (intr) |  | schizophrenia | Korean | yes | (Licinio et al., 2009; Zakharyan et al., 2014; Park et al., 2011; Gareeva et al., 2015 ) |
|  |  |  | rs4565713-G (intr) |  | schizophrenia | Russian and Tatar | yes | (Gareeva et al., 2015) |
|  |  |  | rs4565713-G (intr) |  | autism | American | yes | (Lu et al., 2013; Chen et al., 2006) |
|  |  |  | rs2856813 (intr), rs4332358 (intr), rs4529705 (intr),  rs6330 (exon), rs6537860 (intr) |  | schizophrenia | Chinese Han | no | (Terry et al., 2010) |
|  |  |  | rs6330-T |  | schizophrenia | Armenian | yes | (Park et al., 2011) |
|  |  |  | rs4839435-A |  | schizophrenia (reduced risk) | Armenian | yes | (Park et al., 2011) |
|  |  |  | rs7523086,  rs12145726 |  | schizophrenia | Russian and Tatar | no | (Lu et al., 2013) |
|  |  |  | rs6330 (A35V) |  | schizophrenia |  | yes | (Kranz et al., 2015) |
|  |  |  | rs2856813-G (intr),  rs6678788-T (intr),  rs4529705-A (intr),  rs6537860-A (intr),  rs4332358-T (intr) or  rs3811014-G,  GG-TC diplotype: rs2856813 (intr) and rs6678788 (intr) |  | Primary affective disorders (PAFDs) | American (woman) | yes | (Cui et al., 2011) |
|  |  |  | CCA haplotype:  rs2254527-C (intr),  rs667878788-C (intr) and  rs12760036-A (intr) |  | major depressive disorder  (lower susceptibility for antidepressant therapy) | Chinese | yes | (Yeh et al., 2015) |
| *NGFR* | Nerve Growth Factor Receptor | predominantly binds the proforms of neurotrophins; triggers apoptotic cascades in neural cells | rs2072446-T (S205L) or  rs11466162-A (3'UTR) |  | schizophrenia |  | yes | (Zhao et al., 2022) |
|  |  |  | rs11466155-T (G265G) or  rs2072446-T (S205L) | Transmembrane domain | schizophrenia | Armenian | yes | (Park et al., 2011) |
|  |  |  | rs734194-G (3'UTR) |  | schizophrenia (reduced risk) | Armenian | yes | (Park et al., 2011) |
|  |  |  | rs2072446-T (S205L) | Extracellular domain | Alzheimer's disease and β-amyloid deposit | Spanish | yes | (He et al., 2022) |
|  |  |  | rs534561-G (intr) |  | addiction formation | Spanish | yes | (Okahisa et al., 2010) |
| *NOTCH4* | Neurogenic Locus Notch Homolog Protein 4 | a receptor for Jagged1/2 and Delta1; regulates differentiation, proliferation and apoptotic programs | rs367398-G (5’UTR) |  | schizophrenia | Indian (women) | yes | (Kumar et al., 2021) |
|  |  |  | rs387071-C (promoter) |  | schizophrenia | Indian (men) | yes | (Kumar et al., 2021) |
| *NRG1* | Neuregulin 1 | mediates the interaction of neurons with glial cells, regulates migration of GABA-producing neurons, synapse formation and myelination | rs35753505-C (promoter) |  | schizophrenia | Iranian and Indian | yes | (Moradkhani et al., 2023) |
|  |  |  | rs6994992-T (promoter) |  | psychosis |  | yes | (Kéri et al., 2009) |
|  |  |  | rs1937970-A (intr) or  rs677221-G (intr) |  | schizophrenia | Chinese (Han) | yes | (Wang et al., 2008) |
|  |  |  | rs10883934-C (intr) or  rs1896506-A (intr) |  | nicotine addiction | American | yes | (Turner et al., 2014) |
| *NRP2* | Neuropilin 2 | a receptor for Sema 3C/3F, VEGFs and PLGF-2; mediates cell guidance and axon pathfinding | rs114144673-T (R334C) | F5/8 type C 1 | Early infantile epileptic encephalopathy (EIEE 13) |  | yes | (The UniProt Consortium, 2023w) |
| *NTF3* | Neurotrophin 3 | brain maturation, ensures the survival of dopaminergic neurons, sensitive neurons of the neural crest | rs1805149-A (G76E) |  | schizophrenia |  | yes | (Hattori and Nanko, 1995) |
|  |  |  | rs6489630-T (3' downstream) |  | lower intelligence scores in children with ADHD |  | yes | (Cho et al., 2010) |
|  |  |  | haplotype:  rs6489630-T (3' downstream) and rs7956189-G (3' downstream) |  | gambling disorder |  | yes | (Solé-Morata et al., 2022) |
| *NTN1* | Netrin 1 | control guidance of CNS commissural axons: attraction upon binding the DCC- and repulsion upon binding the UNC5-receptors; stimulate proliferation, migration, differentiation, and survival of glial cells | rs1567750186-C (C601R) or  rs1567749982 (I518missing) |  | mirror movements 4 condition (MRMV4) |  | yes | (The UniProt Consortium, 2023n) |
| *NTNG2* | Netrin G2 |  | rs1589440982-C (C81Y) or  rs1589441229-G (W107G) | Laminin N-terminal  Laminin N-terminal | Neurodevelopmental disorder with behavioral abnormalities, absent speech, and hypotonia (NEDBBASH) |  | yes | (The UniProt Consortium, 2023o) |
| *NTRK1* | Neurotrophic Receptor Tyrosine Kinase 1 | receptors for NGF and BDNF, respectively | rs556840308-A (G4S) |  | schizophrenia | American | yes | (Kranz et al., 2015) |
|  |  |  | rs6336-T (H568Y) or  rs4661063-A (intr) |  | impaired myelination | rats | yes | (Su et al., 2021) |
|  |  |  | rs6336-T (H568Y) | Protein kinase domain | schizophrenia | American and European | yes | (van Schijndel et al., 2009; van Schijndel et al., 2011) |
| *NTRK2* | Neurotrophic Receptor Tyrosine Kinase 2 |  | rs10868235-T (intr) or  rs1387923-G (3’UTR), |  | schizophrenia | Polish (men) | yes | (Suchanek-Raif et al., 2022) |
|  |  |  | rs1387923-A (3’UTR) or  ATAAT haplotype  (5 *NTRK2*) |  | schizophrenia (reduced risk) | Polish (men) | yes | (Suchanek-Raif et al., 2022) |
|  |  |  | rs1565445-A (intr) |  | schizophrenia (increased risk of suicide) | Polish | yes | (Suchanek-Raif et al., 2022) |
|  |  |  | rs1147193-C (3’downstream) |  | drug addiction | Spanish | yes | (Okahisa et al., 2010) |
|  |  |  | rs11140783-C (intr) or  rs3739570-C (3’UTR) |  | gambling disorder (GD) | Spanish | yes | (Solé-Morata et al., 2022) |
| *NTRK2,*  *ВDNF* | Neurotrophic Receptor Tyrosine Kinase 2, Brain-Derived Neurotrophic Factor |  | GTAGCC haplotype:  5 SNPs of *NTRK2* and rs6265-C (V64V) *BDNF* |  | schizophrenia (reduced risk) | Polish (women) | yes | (Suchanek-Raif et al., 2022) |
|  |  |  | haplotype:  rs1387923-G (3’UTR) and rs2769605-T (3' downstream) for *NTRK2* and rs6265-T (V66M) for *ВDNF* |  | schizophrenia  (increased risk of paranoid) | Chinese | yes | (Tsai, 2004; Tsai, 2007; Hwang et al., 2006) |
| *NTRK3* | Neurotrophic Receptor Tyrosine Kinase 3 | a receptor for NT3,  stimulates survival of dopaminergic neurons, sensitive neurons; promotes multipolar-to-radial migration transition | rs12595249-C (intr),  rs744994-T (promoter) or  rs998636-G (promoter) |  | drug addiction | Spanish | yes | (Okahisa et al., 2010) |
|  |  |  | rs7180942 (intr) |  | eating disorders |  | yes | (Mercader et al., 2008) |
|  |  |  | rs8037291-G (intr) |  | ADHD |  | yes | (Smith et al., 2014) |
|  |  |  | rs1946698-C (intr) |  | schizophrenia | Russian | yes | (Lu et al., 2013) |
| *NXPH1* | Neurexophilin-1 | promotes adhesion between dendrites and axons | rs7801099-G (intr) |  | schizophrenia | Russian | yes | (Lu et al., 2013) |
|  |  |  | rs7801099-A (intr) |  | schizophrenia  (reduced risk) | Russian | yes | (Lu et al., 2013) |
|  |  |  | rs7801099-A (intr) |  | schizophrenia | Tatar | no | (Lu et al., 2013) |
| *PCDH12* | Protocadherin-12 | promote homophilic calcium-dependent aggregation and adhesion at intercellular junctions; regulate cell migration | rs1753211537-A (E39Stop),  rs759794990-A (S147I) or  rs375346212-A (R839Stop) | Cadherin 1 domain  Cadherin 1 domain  Cytoplasmic domain | diencephalic-mesencephalic junction dysplasia syndrome 1 (DMJDS1) |  | yes | ( Nicolas et al., 2017; The UniProt Consortium, 2023s) |
| *PCDH17* | Protocadherin-17 |  | rs9537793-G (3'downstream) |  | depression  bipolar disorder |  | yes | (Chang et al., 2018) |
| *PCDH9* | Protocadherin-9 |  | rs9540720-G (intr) |  | major depressive disorder |  | yes | (Ding et al., 2015) |
| *PLAUR* | Plasminogen Activator, Urokinase Receptor | mediates cell guidance and axon pathfinding;  synapse maturation; synaptic pruning | rs344781 |  | autism |  | yes | (Semina et al., 2016; Semina et al., 2017) |
| *PLXNA1* | Plexin-A1 | coreceptors for SEMA3A/3C/3F/5A/6A/6D; are involved in axon guidance, invasive growth, cell migration and cell shape change | rs576960383-A (P2Q) |  | intellectual disability |  | yes | (UniProt,2023l) |
|  |  |  | rs1204647586-T (Q517Stop), rs2079109807-C (C816R), rs762983679-T (R881W) or  rs576960383-A (P2Q) | Extracellular domain | Dworschak-Punetha neurodevelopmental syndrome (DWOPNED) (impaired intellectual development, speech delay, and behavioral abnormalities) |  | yes | (The UniProt Consortium, 2023l) |
| *PLXNA2* | Plexin-A2 |  | rs752016-T (intr) or  rs1327175-C (intr) |  | schizophrenia | American (European descent) | yes | (The UniProt Consortium, 2023x) |
|  |  |  | rs1553277591-T (G736S) | Extracellular domain | atypical cerebral palsy |  | yes | (The UniProt Consortium, 2023x) |
|  |  |  | rs2782948-T (R5Q),  rs11119014-C (Q57R),  rs3748735-T (A267T),  rs2498028-G (intr),  rs1327175-C (intr),  rs752016-T (intr) or  rs841865-A (intr) | Extracellular domain | schizophrenia | Japanese | no | (Fujii et al., 2007) |
| *PLXNA3* | Plexin-A3 |  | rs149367480-A (E72K) | Extracellular domain | psychiatric and cognitive disorders, including autism |  | yes | (The UniProt Consortium, 2023m) |
|  |  |  | rs200042650-A (R616Q) | Extracellular domain | childhood-onset schizophrenia |  | yes | (The UniProt Consortium, 2023m) |
|  |  |  | rs782528486-G (H668) | Extracellular domain | Neurodevelopmental disorder |  | yes | (The UniProt Consortium, 2023m) |
| *PLXNB3* | Plexin-B3 |  | rs2266879-A (V598I),  rs6643791-C (E1156D) or  rs146832392-A (V1596E) | Extracellular domain  IPT/TIG 3 domain | schizophrenia | German | yes | (The UniProt Consortium, 2023y) |
|  |  |  | rs2091974044-A (T1425N) |  | Neurodevelopmental disorder |  | yes | (The UniProt Consortium, 2023y) |
| *RELN* | Reelin | regulates [neuronal migration](https://en.wikipedia.org/wiki/Neuronal_migration) and layering in the developing brain by controlling [cell–cell interactions](https://en.wikipedia.org/wiki/Cell%E2%80%93cell_interactions); extracellular matrix serine protease | rs7341475-A (intr) |  | schizophrenia (reduced risk) | Caucasian | yes | (Marzan et al., 2021; Li W. et al., 2015) |
|  |  |  | rs262355-A (intr) |  | schizophrenia | Caucasian | yes | (Marzan et al., 2021; Li W. et al., 2015) |
| *ROBO1* | Roundabout, Axon Guidance Receptor, Homolog 1 | receptors for chemorepulsive ligands SLIT1 and SLIT2; participate in cellular migration, axon guidance | rs1017845770-A (R119Stop) | Ig-like C2-type 1 domain | tetralogy of Fallot  (increased risk of anxiety, depressive, bipolar, and sleep disorders) |  | yes | (The UniProt Consortium, 2023q; Hsu et al., 2021) |
|  |  |  | rs765740846-A (D1042V),  rs1704874919-T (P1267Q) or  rs778040289-A (R1420L) | Cytoplasmic domain | intellectual disability |  | yes | (The UniProt Consortium, 2023q) |
|  |  |  | rs199958211-A (S1522L) |  | Autosomal recessive congenital nystagmus-8 (NYS8) |  | yes | (Huang et al., 2022) |
| *ROBO3* | Roundabout, Axon Guidance Receptor, Homolog 3 |  | rs121918275-C (L5P) or  rs121918276-C (I66L) | Extracellular domain | horizontal gaze palsy with progressive scoliosis (HGPPS) |  | yes | (Jen et al., 2004; The UniProt Consortium, 2023z) |
| *SEMA3B* | Semaphorin-3B | guidance molecules; regulate neurite guidance, pruning, synapse formation; regulate the formation and stability of the perineuronal network | rs1701032174-C (E150D) | Sema domain | Neurodevelopmental disorder |  | yes | (The UniProt Consortium, 2023α) |
| *SEMA3D* | Semaphorin-3D |  | rs2190208-C (promoter),  rs1029564-G (intr),  rs17159614-T (intr),  rs12176601-T (intr),  rs6966472-C (intr),  rs17559978-A (intr),  rs17159577-A (intr),  rs17159556-A (intr) or  rs7800072-G (intr) |  | schizophrenia | Japanese | no | (Fujii et al., 2011) |
| *SEMA3E* | Semaphorin-3E |  | rs370543242-C (P52R) | Sema domain | Coloboma, heart defects, choanal atresia, growth retardation, genital abnormalities, ear abnormalities, mental retardation (CHARGE) |  | yes | (The UniProt Consortium, 2023β) |
| *SEMA6B* | Semaphorin-6B |  | rs1977288029-G (Y385H) | Extracellular domain | psychiatric and cognitive disorders, including autism |  | yes | (The UniProt Consortium, 2023k) |
| *UNC5C* | Unc-5 Netrin Receptor C | receptor for netrin; mediates axon repulsion in the developing nervous system | rs137875858-A (T835M) | Cytoplasmic domain | AD, associated with susceptibility to late-onset disease, increased susceptibility to neuronal cell death |  | yes | (The UniProt Consortium, 2023γ; The UniProt Consortium, 2023δ) |

# Supplementary References

1. Bhalla, K., Luo, Y., Buchan, T., Beachem, M. A., Guzauskas, G. F., Ladd, S., et al. (2008). Alterations in CDH15 and KIRREL3 in patients with mild to severe intellectual disability. *Am J Hum Genet*. 83, 703-713. doi: 10.1016/j.ajhg.2008.10.020.

2. Cappello, S., Gray, M. J., Badouel, C., Lange, S., Einsiedler, M., Srour, M., et al. (2013). Mutations in genes encoding the cadherin receptor-ligand pair DCHS1 and FAT4 disrupt cerebral cortical development. *Nat Genet*. 45, 1300-1308. doi: 10.1038/ng.2765.

3. Chang, H., Hoshina, N., Zhang, C., Ma, Y., Cao, H., Wang, Y., et al. (2018). The protocadherin 17 gene affects cognition, personality, amygdala structure and function, synapse development and risk of major mood disorders. *Mol Psychiatry*. 23, 400-412. doi: 10.1038/mp.2016.231.

4. Ding, Y., Chang, L. C., Wang, X., Guilloux, J. P., Parrish, J., Oh, H., et al. (2015). Molecular and Genetic Characterization of Depression: Overlap with other Psychiatric Disorders and Aging. *Mol Neuropsychiatry*. 1, 1-12. doi: 10.1159/000369974.

5. Fujii, T., Iijima, Y., Kondo, H., Shizuno, T., Hori, H., Nakabayashi, T., et al. (2007). Failure to confirm an association between the PLXNA2 gene and schizophrenia in a Japanese population. *Prog Neuropsychopharmacol Biol Psychiatry*. 31, 873-877. doi: 10.1016/j.pnpbp.2007.01.027.

6. Fujii, T., Uchiyama, H., Yamamoto, N., Hori, H., Tatsumi, M., Ishikawa, M., et al. (2011). Possible association of the semaphorin 3D gene (SEMA3D) with schizophrenia. *J Psychiatr Res*. 45, 47-53. doi: 10.1016/j.jpsychires.2010.05.004.

7. Huang, Y., Ma, M., Mao, X., Pehlivan, D., Kanca, O., Un-Candan, F., et al. (2022). Novel dominant and recessive variants in human ROBO1 cause distinct neurodevelopmental defects through different mechanisms. *Hum Mol Genet*. 31, 2751-2765. doi: 10.1093/hmg/ddac070.

8. Jen, J.C.; Chan, W.M.; Bosley, T.M. (2004). Mutations in a human ROBO gene disrupt hindbrain axon pathway crossing and morphogenesis. *Science*. 304, 1509-1513. doi: 10.1126/science.1096437.

9. Kumar, Pvsn Kiran, Mitra, Prasenjit, Ghosh, Raghumoy, Saikiran, Gangam, Chambial, Shailja, Nebhinani, Naresh; Sharma, Praveen. (2021). Association of the NOTCH4 gene polymorphism with schizophrenia in the Indian population. *Meta Gene*. 29. doi: 10.1016/j.mgene.2021.100903.

10. Li, D., March, M. E., Fortugno, P., Cox, L. L., Matsuoka, L. S., Monetta, R., Bhoj, E. J.,et al. (2021). Pathogenic variants in CDH11 impair cell adhesion and cause Teebi hypertelorism syndrome*. Hum Genet*. 140, 1061-1076. doi: 10.1007/s00439-021-02274-3.

11. Li, W., Guo, X., Xiao, S. (2015). Evaluating the relationship between reelin gene variants (rs7341475 and rs262355) and schizophrenia: A meta-analysis. *Neurosci Lett*. x, 42-47. doi: 10.1016/j.neulet.2015.10.014.

12. Li, X., Wang, Q., He, K., Li, Z., Chen, J., Li, W., et al. (2014). Common variants in the CDH7 gene are associated with major depressive disorder in the Han Chinese population. Behav. Genet. 44, 97–101. doi: 10.1007/s10519-014-9645-y

13. Marzan, S., Aziz, M.A., Islam, M.S. (2021). Association Between REELIN Gene Polymorphisms (rs7341475 and rs262355) and Risk of Schizophrenia: an Updated Meta-analysis. *J Mol Neurosci*. 71, 675-690. doi: 10.1007/s12031-020-01696-4.

14. The UniProt Consortium. (2023r). UniProt: the Universal Protein Knowledgebase. Cadherin-2. https://www.UniProt.org UniProtkb/P19022/variant-viewer [Accessed October 25, 2023].

15. The UniProt Consortium. (2023t). UniProt: the Universal Protein Knowledgebase. Cadherin-11. https://www.UniProt.org/UniProtkb/P55287/variant-viewer [Accessed October 25, 2023].

16. The UniProt Consortium. (2023u). UniProt: the Universal Protein Knowledgebase. Cadherin-15. https://www.UniProt.org/UniProtkb/P55291/variant-viewer [Accessed October 25, 2023].

17. The UniProt Consortium. (2023v). UniProt: the Universal Protein Knowledgebase. Cadherin-5. https://www.UniProt.org/UniProtkb/P33151/variant-viewer [Accessed October 25, 2023].

18. The UniProt Consortium. (2023w). UniProt: the Universal Protein Knowledgebase. Neuropilin-2. https://www.UniProt.org/UniProtkb/O60462/variant-viewer [Accessed October 25, 2023].

19. The UniProt Consortium. (2023x). UniProt: the Universal Protein Knowledgebase. Plexin-A2. https://www.UniProt.org/UniProtkb/O75051/variant-viewer [Accessed October 25, 2023].

20. The UniProt Consortium. (2023y). UniProt: the Universal Protein Knowledgebase. Plexin-B3. https://www.UniProt.org/UniProtkb/Q9ULL4/variant-viewer [Accessed October 25, 2023].

21. The UniProt Consortium. (2023z). UniProt: the Universal Protein Knowledgebase. Roundabout homolog 3. https://www.UniProt.org/UniProtkb/Q96MS0/variant-viewer [Accessed October 25, 2023].

22. The UniProt Consortium. (2023α). UniProt: the Universal Protein Knowledgebase. Semaphorin-3B. https://www.UniProt.org/UniProtkb/Q13214/variant-viewer [Accessed October 25, 2023].

23. The UniProt Consortium. (2023β). UniProt: the Universal Protein Knowledgebase. Semaphorin-3E. https://www.UniProt.org/UniProtkb/O15041/variant-viewer [Accessed October 25, 2023].

24. The UniProt Consortium. (2023γ). UniProt: the Universal Protein Knowledgebase. Netrin receptor UNC5C. https://www.UniProt.org/UniProtkb/O95185/variant-viewer [Accessed October 25, 2023].

25. Vieira, R. N., Ávila, R., de Paula, J. J., Cintra, M. T., de Souza, R. P., Nicolato, R., et al. (2016). Association between DCHS2 gene and mild cognitive impairment and Alzheimer's disease in an elderly Brazilian sample. *Int J Geriatr Psychiatry*. 31, 1337-1344. doi: 10.1002/gps.4440.
